# *C*_S_-Symmetric Pyridine(diimine) Iron Methyl Complexes for Catalytic [2+2] Cycloaddition and Hydrovinylation: Metallacycle Geometry Determines Selectivity

**DOI:** 10.1021/jacsau.3c00229

**Published:** 2023-07-12

**Authors:** Coralie Duchemin, Junho Kim, Paul J. Chirik

**Affiliations:** Department of Chemistry, Princeton University, Princeton, New Jersey 08544, United States

**Keywords:** iron, metallacycle, ethylene, cycloaddition, hydrovinylation

## Abstract

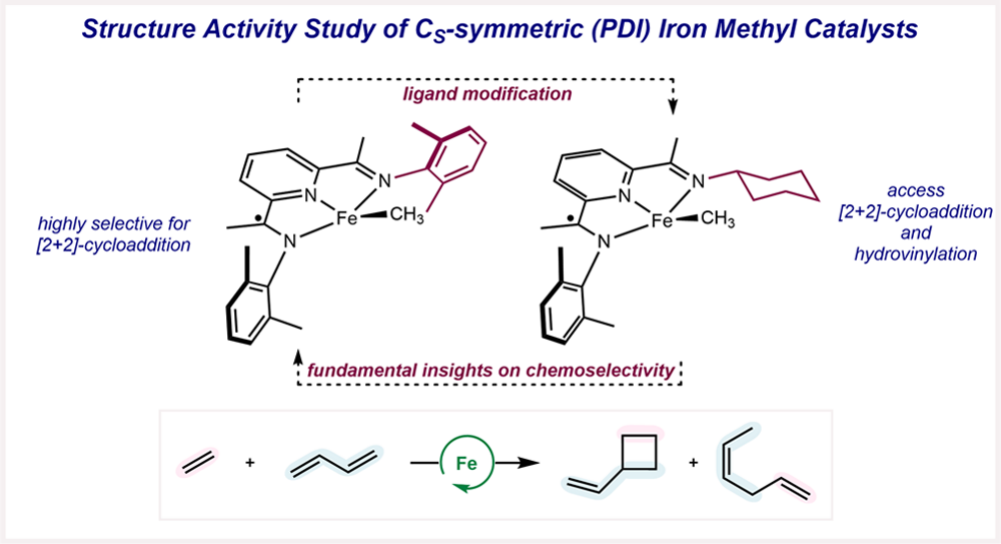

A series of *C*_S_-symmetric (aryl,alkyl)-substituted pyridine(dimine) iron methyl (^Cy^A^R^PDI)FeCH_3_ complexes have been prepared, characterized, and evaluated as precatalysts for the [2+2]-cycloaddition of butadiene and ethylene. Mixtures of vinylcyclobutane and (*Z*)-hexa-1,4-diene were observed in each case. By comparison, *C*_2v_-symmetric, arylated (PDI) iron catalysts are exclusively selective for reversible [2+2]-cycloaddition to yield vinylcyclobutane. The alteration in the chemoselectivity of the catalytic reaction was investigated through a combination of precatalyst stability studies, identification of catalytic resting state(s), and ^2^H and ^13^C isotopic labeling experiments. While replacement of an aryl-imine substituent with an N-alkyl group decreases the stability of the formally iron(0) dinitrogen and butadiene complexes, two diamagnetic metallacycles were identified as catalyst resting states. Deuterium labeling and NOESY/EXSY NMR experiments support 1,4-hexadiene arising from catalytic hydrovinylation involving reversible oxidative cyclization leading to accessible *cis*-metallacycle. Cyclobutane formation proceeds by irreversible C(sp^3^)–C(sp^3^) bond-forming reductive elimination from a *trans*-metallacycle. These studies provide key mechanistic understanding into the high selectivity of bis(arylated) pyridine(diimine) iron catalysts for [2+2]-cycloaddition, unique, thus far, to this class of iron catalysts.

The catalytic transformation of feedstock olefins and dienes to value-added chemicals is of long-standing interest for the synthesis of small molecules and polymers.^1^ Molecular catalysts offer the distinct advantage of rational ligand modification to alter and optimize catalyst performance.^2−4^ The independent discoveries by Brookhart and Gibson that activation of high-spin pyridine(diimine) (PDI) iron(II) and cobalt(II) complexes with methylaluminoxane (MAO) generates highly active catalysts for the polymerization of ethylene and selected α-olefins, demonstrated the rich C–C bond-forming chemistry available from open-shell, first-row transition-metal catalysts.^5,6^ Since these discoveries and those with bidentate α-diimine ligands by tom Dieck^7^ and co-workers, a host of iron and cobalt complexes have been explored for catalytic C–C bond-forming chemistry.^8−17^ Examples of iron-catalyzed [2+2]-,^18^ [4+2]-,^19^ and [4+4]-cycloaddition^20,21^ processes using commodity olefins and dienes have been reported, often with high chemo- and stereoselectivities.

Reduced *C*_2v_-symmetric, aryl-substituted pyridine(diimine) complexes such as (^Me^PDI)Fe(N_2_)_2_ (^Me^PDI = 2,6-(2,6-Me-C_6_H_3_N = CMe)_2_C_5_H_3_N) promote the reversible, catalytic [2+2] cycloaddition of ethylene and butadiene to form vinylcyclobutane,^22^ with near-exclusive chemoselectivity for the cyclized product.^23^ Experimental mechanistic studies^24,25^ from our laboratory combined with computational insights^26^ have established catalyst design principles for this unique reactivity: (i) a planar chelate that enforces butadiene to coordinate in an s-*trans*-geometry at the metal and (ii) access to triplet ground states. With iron catalysts bearing redox-active (PDI) ligands, these are thermally accessible. This method was applied to the [2+2] cyclooligomerization of butadiene to furnish divinyl(oligocyclobutane), a chemically recyclable microstructure of polybutadiene (Scheme 1A).^27^ While *in situ*-activated (PDI) iron dihalide complexes do not provide selective cycloaddition, the isolation of (PDI) iron dinitrogen or butadiene was, until recently, crucial for the success of the transformation.

**Scheme 1:**
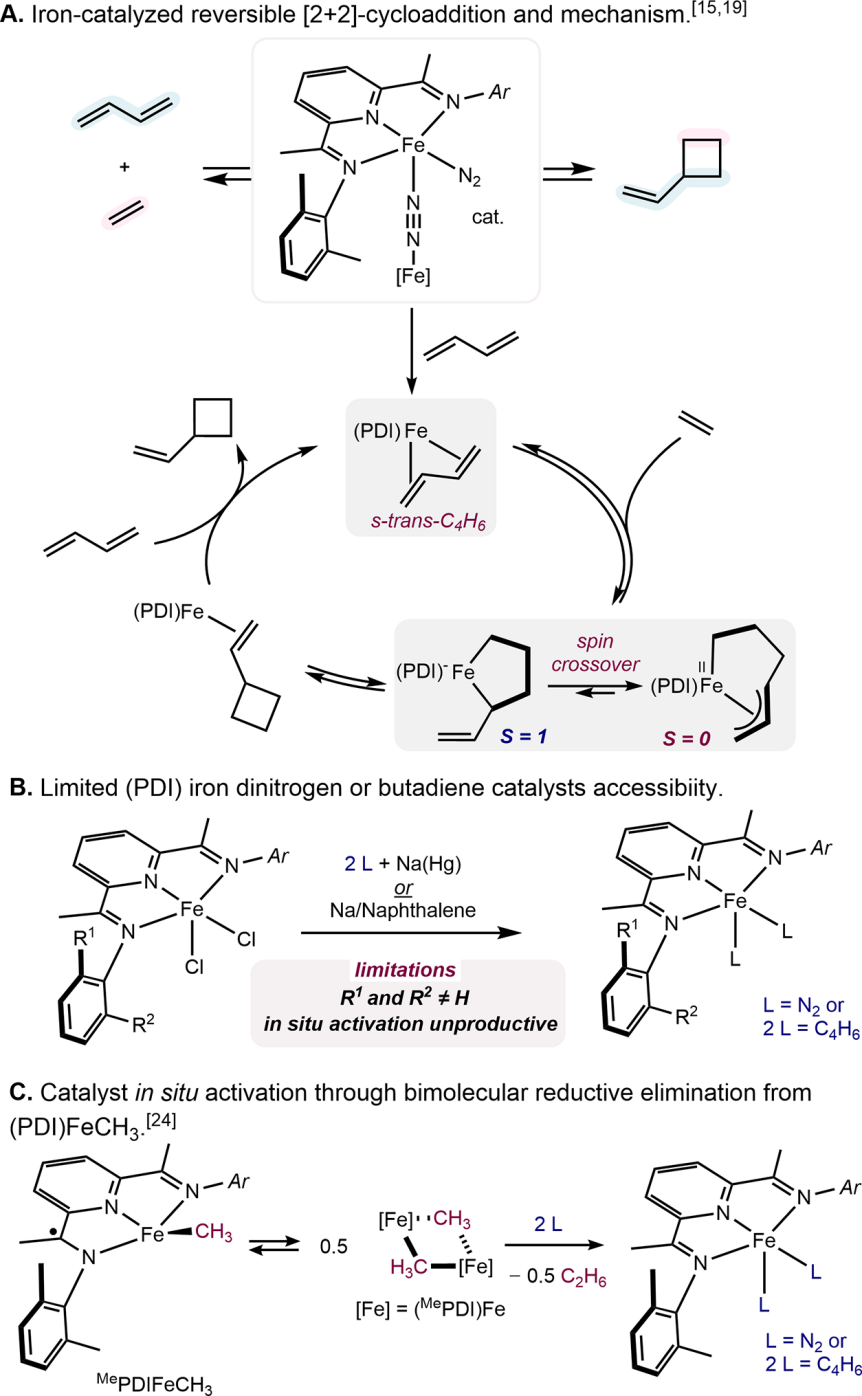
Pyridine(diimine) Iron Catalysts for the Selective [2+2]-Cycloaddition of Feedstock Olefins and Dienes

Pyridine(diimine) ligands are readily synthesized and electronically and sterically modular.^28^ As such, (PDI) iron and cobalt dihalide complexes are widely available as precatalysts for the polymerization of ethylene and α-olefins.^29^ By comparison, few pyridine(diimine) iron dinitrogen or butadiene complexes have been prepared (Scheme 1B),^30^ owing to the lack of general synthetic methods and the propensity to form catalytically inactive bis(chelate) iron derivatives.^31^ A new synthetic route to [(^Me^PDI)Fe(N_2_)]_2_(μ-N_2_) and (^Me^PDI)Fe(*trans*- η^4^-C_4_H_6_) was recently reported and relied on the bimolecular reductive elimination of ethane from the corresponding iron methyl derivative, (^Me^PDI)FeCH_3_ (Scheme 1C).^32^ This method enabled the direct entry into catalytic [2+2]-cycloaddition as (^Me^PDI)FeCH_3_ underwent *in situ* activation to form the cycloaddition catalyst. Avoiding the need for isolation of formally (PDI) iron(0) catalysts, therefore, offers the opportunity to explore the scope of iron catalysts capable of [2+2]-cycloaddition. To date, all known iron cycloaddition precatalysts are restricted to those bearing 2,6-disubstituted anilines, a consequence of the ability to support formally iron(0) dinitrogen complexes.^33−35^ More significant modification to the iron precatalyst structure is desired to broaden the scope of accessible catalyst space and to develop stereocontrolled cycloadditions and oligomerizations. Specifically, replacing one N-aryl-imine substituent by an aliphatic amine would enable introduction of modular groups onto the [2+2]-cycloaddition iron catalyst. It would allow exploration of the steric and electronic features of the metal–ligand combination that enables the unique and highly selective [2+2]-cycloaddition chemistry.

Here, we describe the exploration of the *in situ* iron precatalyst activation approach for the synthesis, stoichiometric reactivity, and catalytic performance study of a series of *C*_S_-symmetric aryl,alkyl-substituted (^Cy^A^R^PDI)FeCH_3_ complexes (Scheme 2). Catalytic conversion of butadiene and ethylene to a mixture of vinylcyclobutane (VCB) and (*Z*)-hexa-1,4-diene (1,4-HD) was observed arising from [2+2] cycloaddition and hydrovinylation, respectively. Studies into the reactivity of (^Cy^A^R^PDI)FeCH_3_ complexes established that formally iron(0) complexes are thermally unstable, highlighting the utility of the bimolecular reductive elimination catalyst activation mode. The origin of the two hydrocarbon products was investigated through identification of the catalyst resting state and isotopic labeling experiments. Comparisons to established *C*_2v_-symmetric bis(arylated) pyridine(diimine) iron cycloaddition catalysts provided fundamental insights into the origin of the unique [2+2] cycloaddition reactivity.

**Scheme 2:**
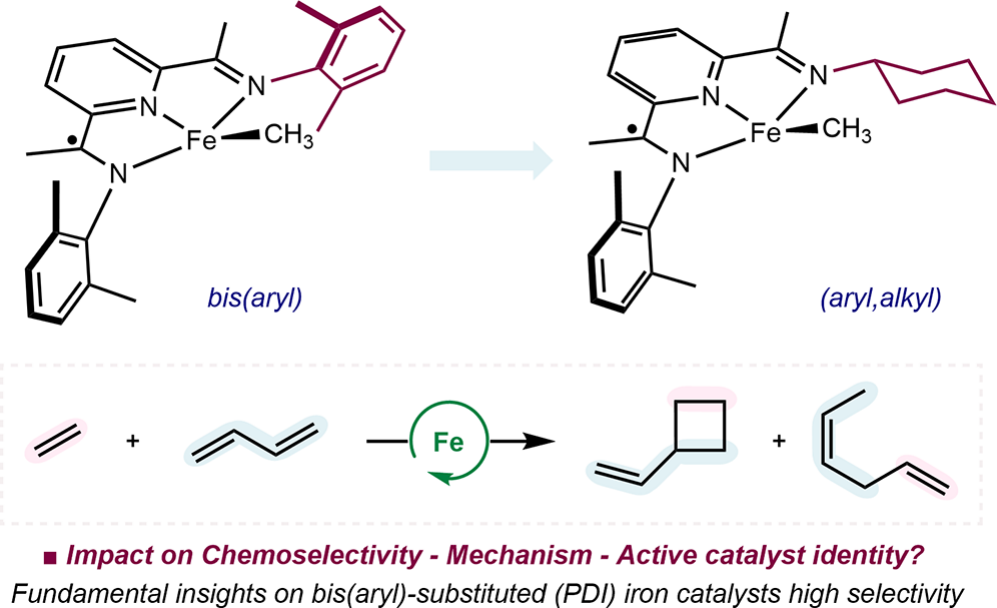
Pyridine(diimine) Modifications and Catalytic Reactivity with Butadiene and Ethylene

## Results and Discussion

### Synthesis and Characterization of (^Cy^A^R^PDI)FeCH_3_ Complexes

**Scheme 3:**
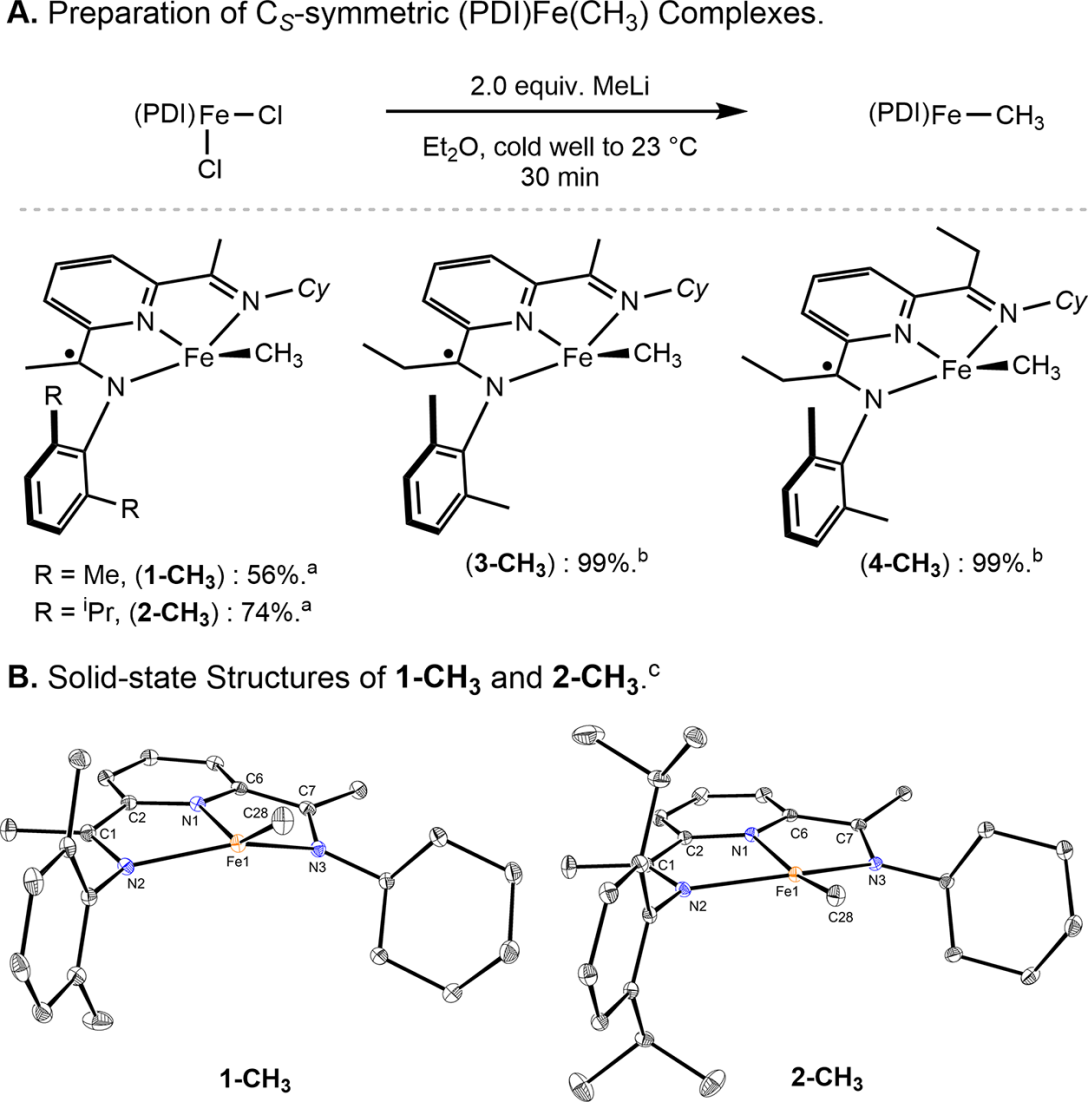
Preparation and Solid-State Structures of *C*_*S*_-Symmetric (PDI)FeCH_3_ Complexes,, Yield of the recrystallized material. Obtained as a crude product. Representation of the solid-state structure of **1-CH**_**3**_ and **2-CH**_**3**_ with thermal ellipsoids at a 30% probability. Hydrogen atoms are omitted for clarity.

Initial attempts to access *C*_S_-symmetric iron(0) catalysts through known reduction methods of the corresponding iron(II) dihalides with sodium amalgam, sodium naphthalene, or magnesium(butadiene) were unsuccessful. Attention was therefore directed to the synthesis of the *C*_S_-symmetric iron methyl complexes: (^Cy^A^Me^PDI)FeCH_3_ (**1-CH**_**3**_),^36^ (^Cy^A^iPr^PDI)FeCH_3_ (**2-CH**_**3**_), (^Cy^A^Me^(Me,Et)PDI)FeCH_3_ (**3-CH**_**3**_), and (^Cy^A^Me^(Et)PDI)FeCH_3_ (**4-CH**_**3**_) using the procedure reported for (^Me^PDI)FeCH_3_.^32^ Variations at the aniline and at the imine backbone substituents were selected to tune the solubility and steric environment of the resulting iron catalyst. Treatment of each of the corresponding iron(II) dihalide precursors, previously reported by Bianchini and co-workers,^37^ with two equivalents of methyllithium resulted in the formation of dark green solutions from which **1-CH**_**3**_ and **2-CH**_**3**_ were isolated as dark green solids following recrystallization at −35 °C in 56 and 74% yields, respectively (Scheme 3A). Examples with ethyl substituents in the 2,6-imine backbone positions, **3-CH**_**3**_ and **4-CH**_**3**_, were also obtained as clean products following methylation of the iron dihalide. The high solubility of **3-CH**_**3**_ and **4-CH**_**3**_ in common organic solvents rendered crystallization challenging. All four complexes exhibited paramagnetically broadened and shifted ^1^H NMR spectra in cyclohexane-*d*_12_ and benzene-*d*_6_. Accordingly, solution (cyclohexane-*d*_12_, 23 °C) magnetic moments of 4.0 μ_B_ were measured for **1-CH**_**3**_ and **2-CH**_**3**_ and are consistent with the spin-only value for three unpaired electrons and high-spin iron complexes.

**Figure 1:**
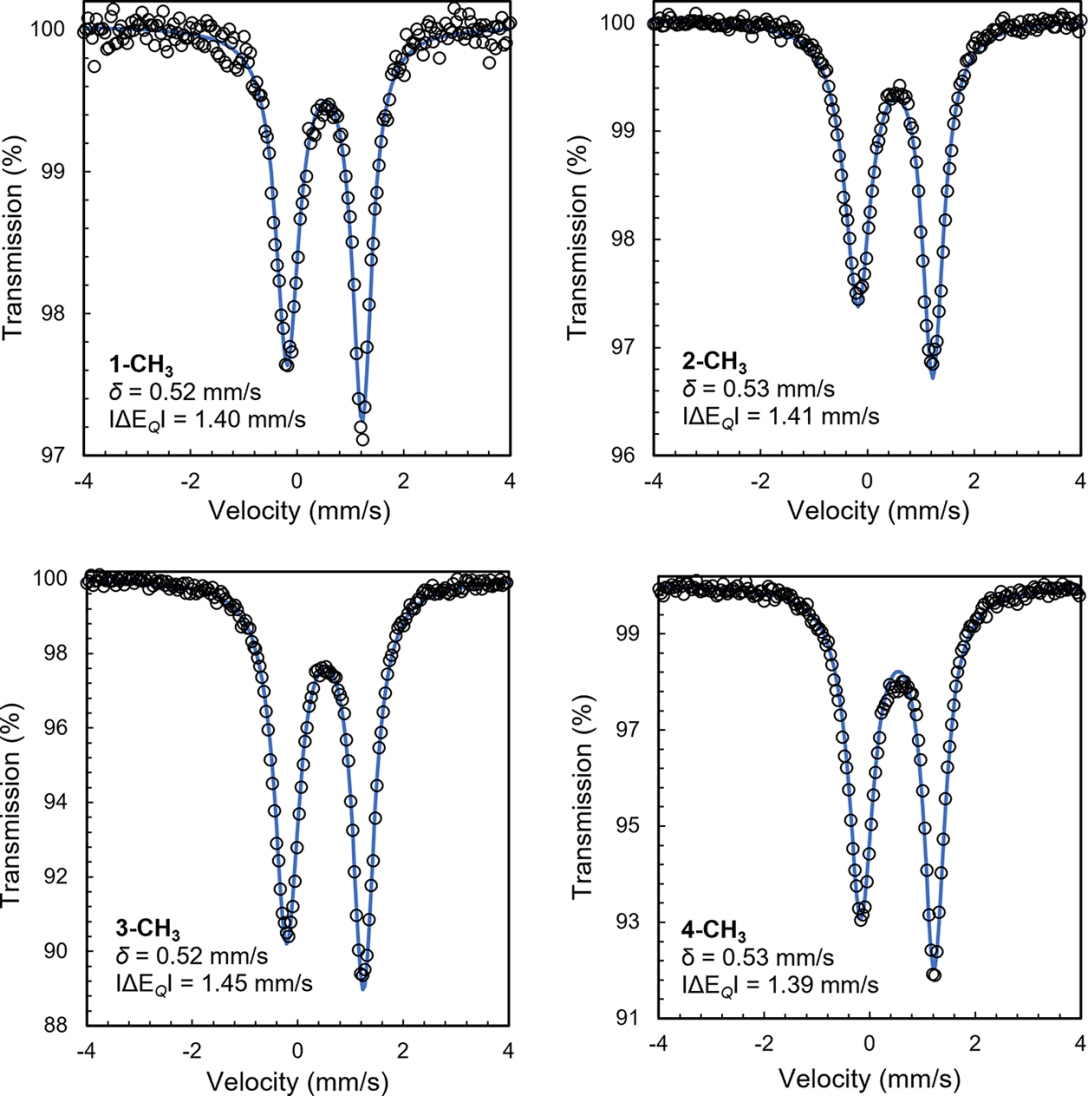
Zero-field ^57^Fe Mössbauer spectra of **1-CH**_**3**_, **2-CH**_**3**_, **3-CH**_**3**_, and **4-CH**_**3**_ recorded at 80 K.

Single crystals suitable for X-ray diffraction of **1-CH**_**3**_ and **2-CH**_**3**_ were obtained from diethyl ether and pentane solutions stored at −35 °C. The solid-state structures (Scheme 3B) confirm the identity of the molecules as four-coordinate iron methyl complexes with N_pyridine_–Fe–C angles of 145.95(7)° (**1-CH**_**3**_) and 164.94(6)° (**2-CH**_**3**_). Zero-field ^57^Fe Mössbauer spectra were recorded in the solid state at 80 K and exhibit quadrupole doublets with isomer shifts (δ) of 0.52–0.53 mm/s and quadrupole splittings (Δ*E*_Q_) between 1.39 and 1.45 mm/s (Figure 1). Geometries distorted from planarity where the methyl group is lifted from the iron-chelate plane and the Mössbauer values measured are consistent with high-spin iron(II) centers (S_Fe_ = 2) antiferromagnetically coupled to bis(imino)pyridine radical anions (S_PDI_ = 1/2).^32^

### Catalytic Performance of (^Cy^A^R^PDI)FeCH_3_ Complexes in C–C Bond-Forming Reactions with Butadiene and Ethylene

The performance of *C*_s_-symmetric pyridine(diimine) iron methyl complexes as precatalysts for alkene-diene [2+2] cycloaddition was evaluated (Table 1). The [2+2]-cycloaddition of ethylene and butadiene was selected to compare the series of *C*_S_-symmetric (PDI)FeCH_3_ complexes to the (bis)arylated (^Me^PDI)FeCH_3_ precatalysts. The latter provided VCB exclusively after 16 h (entry 1).^32^ The addition of an equimolar mixture of butadiene and ethylene to a benzene-*d*_6_ containing 5 mol % of **1-CH**_**3**_ resulted in a 48% conversion of butadiene after 6 h (entry 2) and complete consumption of the substrates over the course of 14 h. A 51:47 mixture of vinylcyclobutane to 1,4-hexadiene was obtained as judged by NMR spectroscopy. The balance of the material was 2,4-hexadiene, likely arising from isomerization of the latter product (entry 3). The ratio of the [2+2] cycloaddition product to [1,4]-hydrovinylation remained constant over the course of the reaction, and the 2,4-hexadiene formed only after the completion of the catalytic reaction, suggesting that it arises from iron-hydrides derived from catalyst decomposition.^12^

**Table 1:**
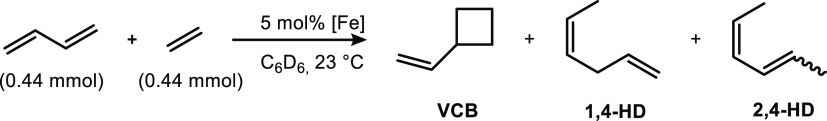
Catalytic Performance of *C*_*S*_-Symmetric (PDI)FeCH_3_ Precatalysts

				product ratio		
entry	[Fe]	*t* [h]	C_4_H_6_ conv. [%]	VCB	1,4-HD	2,4-HD
1^32^	**(**^**Me**^**PDI)FeCH**_**3**_	16	>99	>99		
2	**1-CH**_**3**_	6	48	51	49	0
3	**1-CH**_**3**_	14	>99	51	47	2
4	**2-CH**_**3**_	6	74	14	84	2
5	**2-CH**_**3**_	20	>99	11	68	21
6	**3-CH**_**3**_	6	68	51	49	0
7	**3-CH**_**3**_	11	>99	52	42	6
8	**4-CH**_**3**_	6	60	50	50	0
9	**4-CH**_**3**_	11	>99	48	48	6

Using **2-CH**_**3**_ where the 2,6-aniline substitutes are isopropyl groups, a 74% conversion was obtained after 6 h with detection of the 2,4-hexadiene isomer. Complete consumption of the substrates was observed after 20 h at ambient temperature, and a 11:68:21 ratio of products was obtained (entries 4 and 5). Introduction of ethyl substituents on the imine carbon as in the case of precatalysts **3-CH**_**3**_ and **4-CH**_**3**_ increased the rate as observed at 6 and 10 h of reaction with similar ratios of cycloaddition to hydrovinylation products (entries 6–9). The slight increase in the rate of C–C bond formation observed with **3-CH**_**3**_ and **4-CH**_**3**_ may be due to the improved solubility of these compounds as compared to that of **1-CH**_**3**_. The discrepancy in chemoselectivity resulting from substitution from one N-aryl-imine to a cyclohexyl amine motivated an in-depth study into the reaction mechanism with the goal of understanding the origins of the various C–C bond-forming processes.

### Exploring the Mechanism of Hydrovinylation

**Scheme 4:**
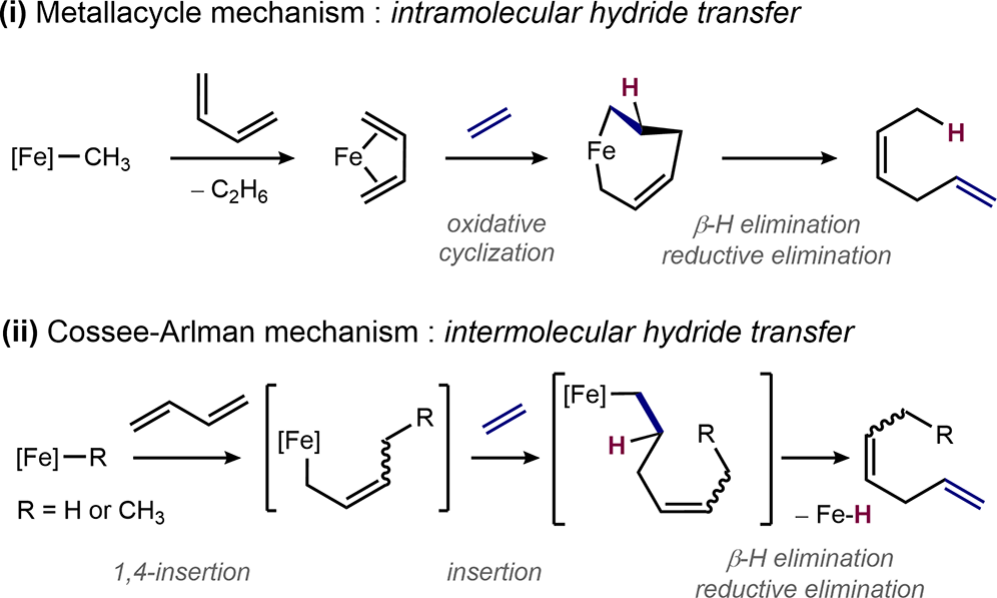
Proposed Mechanism for the Formation of (*Z*)-Hexa-1,4-diene

To understand the origin of the observed chemoselectivity, gaining insight into the pathway operative for catalytic hydrovinylation was of first interest and why the introduction of an N-cyclohexyl substituent affords acyclic products. Distinguishing between a Cossee–Arlman (coordination–insertion) mechanism^4^ involving an iron hydride active species versus an oxidative cyclization–reductive elimination pathway involving metallacycle intermediates was experimentally investigated (Scheme 4). Deuterium labeling studies on the selective, intermolecular hydrovinylation of dienes with α-olefins catalyzed by α-diimine iron catalysts were previously reported, which are consistent with a pathway involving the oxidative cycloaddition of the alkene with an iron diene complex.^12^

**Figure 2:**
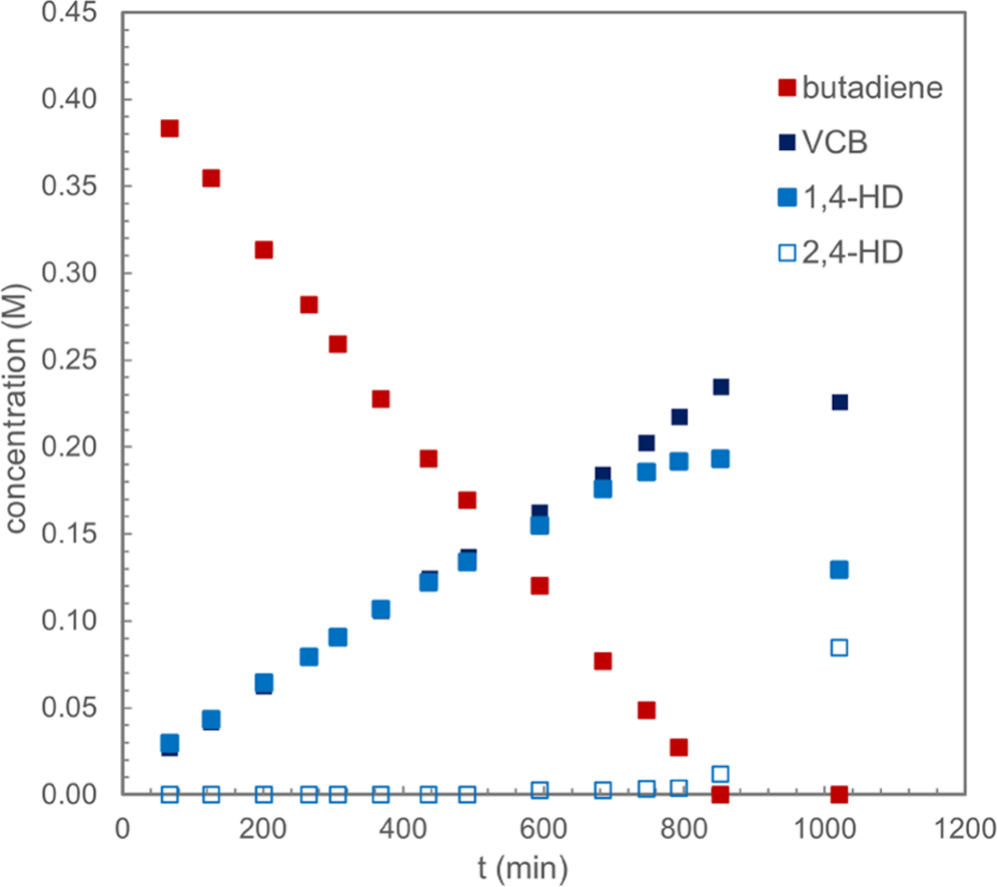
Time course of the reaction with 5 mol % of **1-CH**_**3**_.

The hydrovinylation pathway may arise from (i) precatalyst decomposition forming an iron hydride or (ii) the inherent features of the *C*_s_-symmetric (PDI) iron methyl complexes and their corresponding active species. A time course monitoring the consumption of substrates and formation of hydrocarbon products is presented in Figure 2. The data established that the isomerization of 1,4-hexadiene to 2,6-hexadiene occurred following an ∼80% conversion of butadiene, consistent with the conjugated diene forming in a secondary catalytic process arising from the decomposition of the active catalyst to an observed iron hydride.

**Scheme 5:**
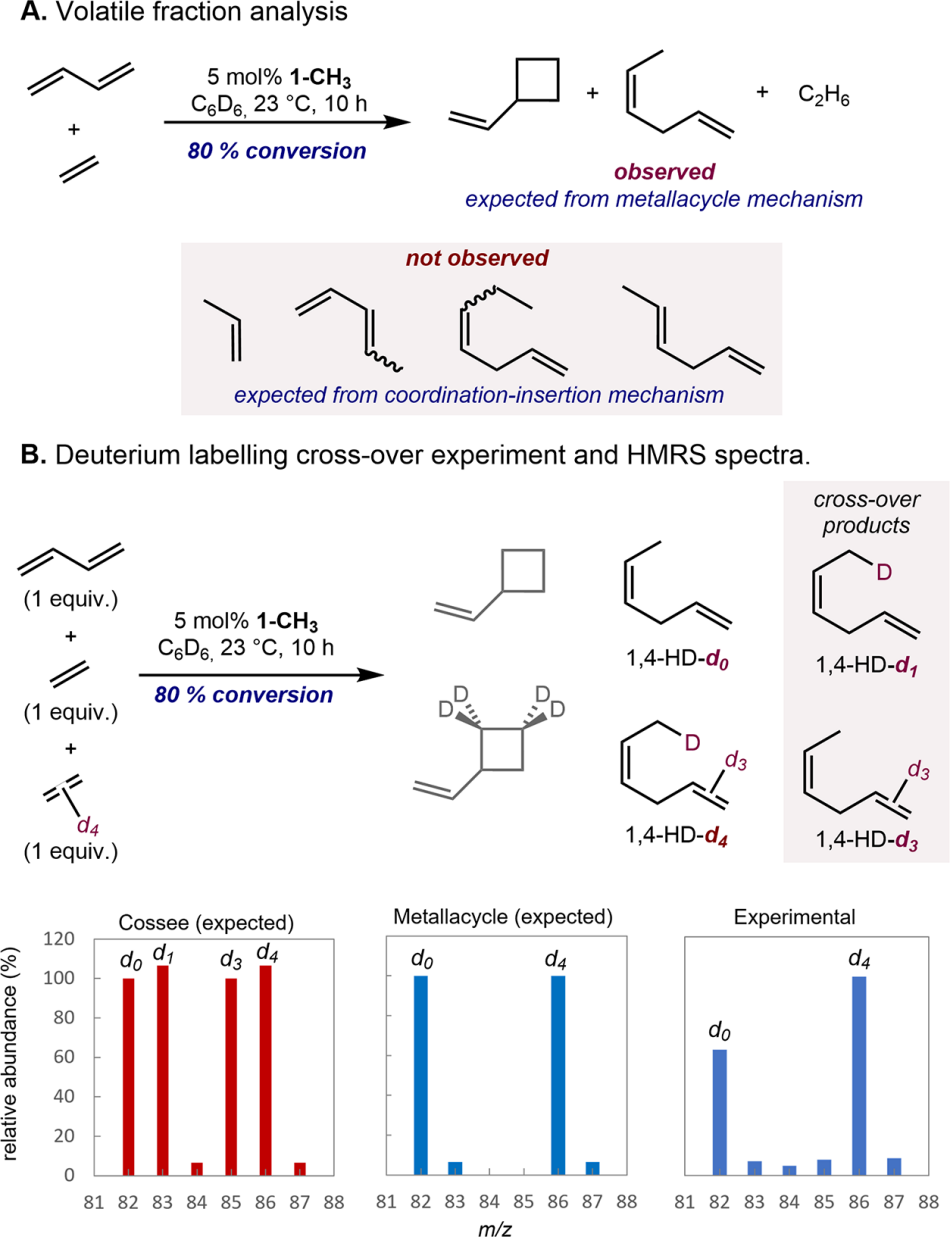
Evidence Supporting a Metallacycle Mechanism for the Formation of (*Z*)-Hexa-1,4-diene

To evaluate the mode of activation of the iron methyl precatalyst, analysis of the volatile products obtained following the activation of **1-CH**_**3**_ under catalytic conditions was examined (Scheme 5A).^38^ If a Cossee–Arlman mechanism were operative, the formation of 5 mol % of propylene, piperylene, or 1,4-heptadiene is expected from insertion into the iron methyl bond followed by β-H elimination. The metallacycle alternative is expected to initiate from bimolecular reductive elimination of ethane to generate a formally iron(0) active species. Analysis of the volatiles following catalyst activation with ethylene and butadiene by ^1^H NMR spectroscopy established the exclusive formation of ethane in addition to the expected C6 products arising from the catalytic reaction. In addition, a Cosse-type mechanism is expected to form an iron allyl that would rapidly isomerize prior to ethylene insertion, resulting in a mixture of hexadiene isomers. Taken together, these observations support a metallacycle pathway until decomposition of the iron catalyst becomes significant to form a presumed iron hydride that is responsible for diene isomerization.

Additional evidence for a metallacycle intermediate was provided by a cross-over experiment using the natural abundance and *d*_4_ isotopologues of ethylene. This approach was pioneered by Bercaw, Labinger, and co-workers and Belov et al. to establish the role of metallacycles in chromium-^39^ and titanium-catalyzed^40^ ethylene upgrading reactions, respectively. In the context of the [1,4]-hydrovinylation of butadiene with either natural abundance ethylene or ethylene-*d*_4_, the distribution of 1,4-hexadiene isotopologues distinguishes the two pathways. If a metallacycle mechanism is operative, a mixture of only *d*_0_ and *d*_4_ hydrovinylation products is expected, while a coordination–insertion mechanism involving an intermolecular hydride transfer would form a statistical mixture of *d*_0_, *d*_1_, *d*_3_, and *d*_4_ products. Stirring a benzene-*d*_6_ solution of butadiene with an equimolar 1:1 mixture of C_2_H_4_ and C_2_D_4_ in the presence of 5 mol % of **1-CH**_**3**_ produced the expected mixture of vinylcyclobutane and 1,4-hexadiene. The exclusive formation of the expected isotopomers was observed by ^13^C NMR spectroscopy, ruling out H/D scrambling due to iron hydride formation. The exclusive formation of the expected isotopomers was observed by ^13^C NMR spectroscopy, ruling out H/D scrambling due to iron hydride formation. However, a control experiment was performed with only ethylene-*d*_4_ and produced an isotopic distribution pattern among *d*_0_, *d*_1_, *d*_2_, *d*_3_, and *d*_4_ products (see the Supporting Information). Analysis of the remaining (PDI) ligand established that H/D scrambling events likely arise from ligand cyclometallation, a background reaction observed previously in [2+2]-cycloadditions.^49^ Analysis of the isotopologues from the cross-over experiment in combination with the control experiment, by GC-HRMS, established a majority of *d*_0_ and *d*_4_ products with a minor amount of the *d*_1_, *d*_2_, and *d*_3_ isotopologues, most consistent with a metallacycle mechanism (Scheme 5B).

### Precatalyst Activation: Stoichiometric Reactivity of (^Cy^A^R^PDI)FeCH_3_ Complexes

**Scheme 6:**
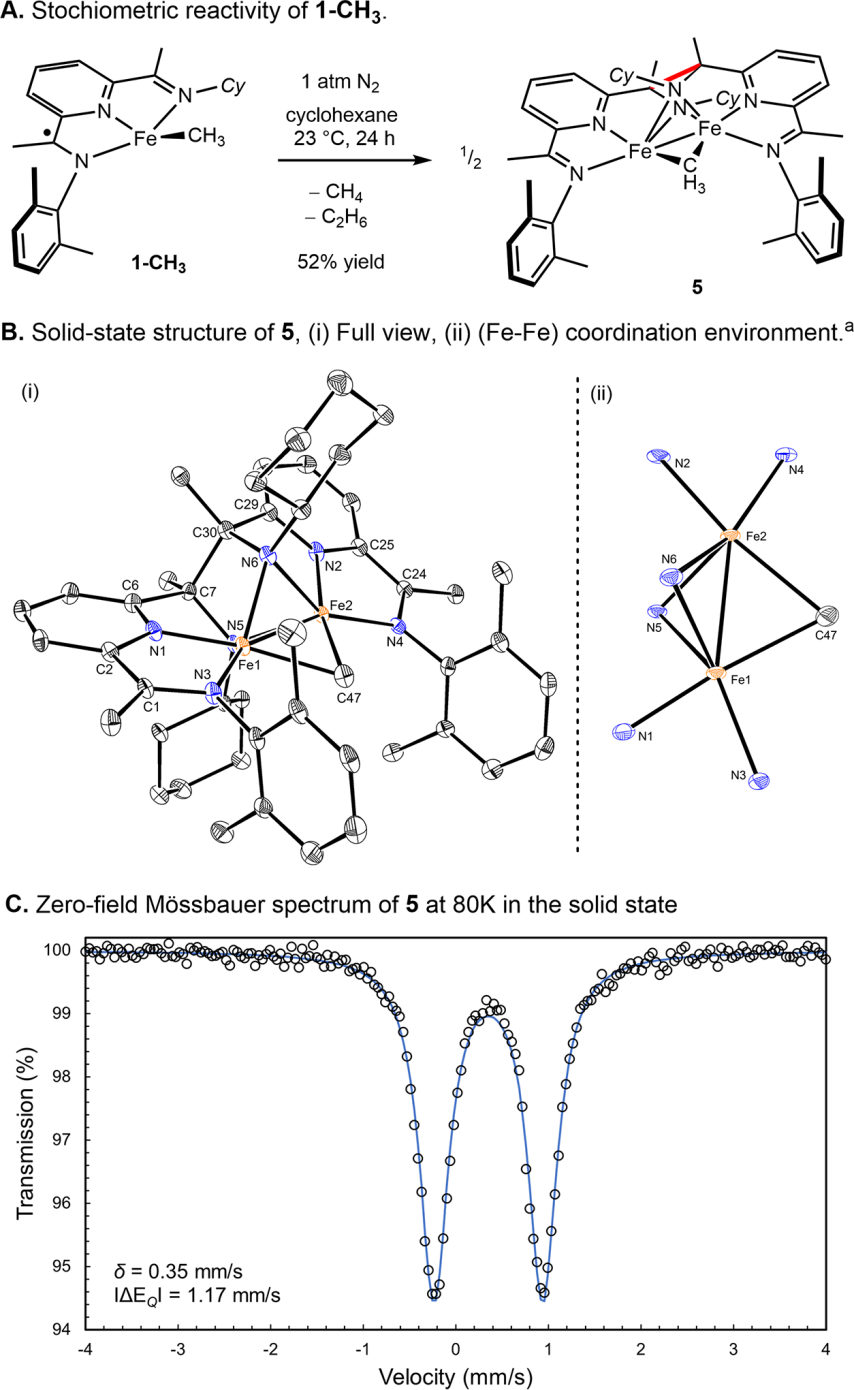
Reductive Elimination Reactivity of **1-CH**_**3**_ in Cyclohexane Representation of the solid-state structure of **5** with thermal ellipsoids at a 30% probability. Hydrogen atoms are omitted for clarity. Selected bond distances (Å) and angles (deg): Fe1–Fe2 2.4172(16); Fe1–N1 1.839 (4), Fe1-N3 1.950 (3), Fe1-N5 1.997 (3), Fe1-N6 2.018 (3), Fe1-C47 2.145 (5), N3-C1 1.336 (5), C1-C2 1.402(5), C7-C30 1.615(6), N1-Fe1-C47 173.31(16), N3-Fe1-N5 144.04(13), N3-Fe1-N6 137.08(14), and N5-Fe1-N6 74.84(13).

Having established a metallacycle mechanism being operative for competitive hydrovinylation, the nature of the *C*_s_-symmetric pyridine(diimine) iron complexes obtained upon precatalyst activation was investigated. The bimolecular reductive elimination chemistry of **(**^**Cy**^**A**^**Me**^**PDI)FeCH**_**3**_**1-CH**_**3**_ was initially explored. Monitoring a benzene-*d*_6_ solution of **1-CH**_**3**_ under a dinitrogen atmosphere by ^1^H NMR spectroscopy established the formation of ethane over the course of several days at 23 °C with no evidence for the formation of an iron dinitrogen derivative. Replacing the benzene-*d*_6_ solvent with cyclohexane-*d*_12_ accelerated the reaction as full conversion was observed after 24 h along with the formation of a paramagnetic iron product (Scheme 4A). Performing the reaction on a preparative scale and recrystallization from diethyl ether at −35 °C resulted in the isolation of the paramagnetic product in a 52% yield (Scheme 6A). The solid-state structure was determined by X-ray diffraction and established the identity of the product as the dinuclear, mixed-valent iron complex **5** formed from the C–C bond formation between the imine carbons on two pyridine(diimine) ligands (Scheme 6B). A bridging methyl group was located between the two iron atoms, the identity of which was confirmed by the addition of CD_3_OD to liberate CH_3_D with no traces of CH_2_D_2_ (see Figure S25).

The newly formed C(sp^3^)–C(sp^3^) bond has a distance of 1.615(6) Å, longer than the value of 1.54 Å expected for a typical C–C bond^41^ but within the range for a long covalent bond.^42^ The distance between the two iron atoms (2.4172(16) Å) is shorter than that found in metallic iron (2.48 Å) and suggests the presence of an Fe–Fe interaction.^43^ The bond distortion of the pyridine(imine) (PI) portion of the ligand is consistent with a one-electron reduction, as compared to neutral and radical anion forms of (PI) ligands.^20^ The C_imine_–N_imine_ distances of 1.336(5) Å and C_ipso_–C_imine_ values of 1.402(5) Å are, respectively, shortened and elongated, compared to the values of (PI) ligand in the neutral form. The zero-field ^57^Fe Mössbauer spectrum of **5** (Scheme 6C) was recorded in the solid state at 80 K and exhibits an isomer shift of 0.35 mm/s and a quadrupole splitting of 1.17 mm/s. These parameters are consistent with a single intermediate-spin ferrous environment for both iron atoms, corroborating the *C*_2V_-symmetry observed in the solid-state structure. A plausible explanation for the formation of **5** consists of a bimolecular reductive elimination forming a putative (PDI) iron dinitrogen complex that in turn reacts with one equivalent of the iron methyl complex. The observation of methane during the formation of **5** is likely due to decomposition of the iron product, consistent with the modest yield of the transformation.

**Scheme 7:**
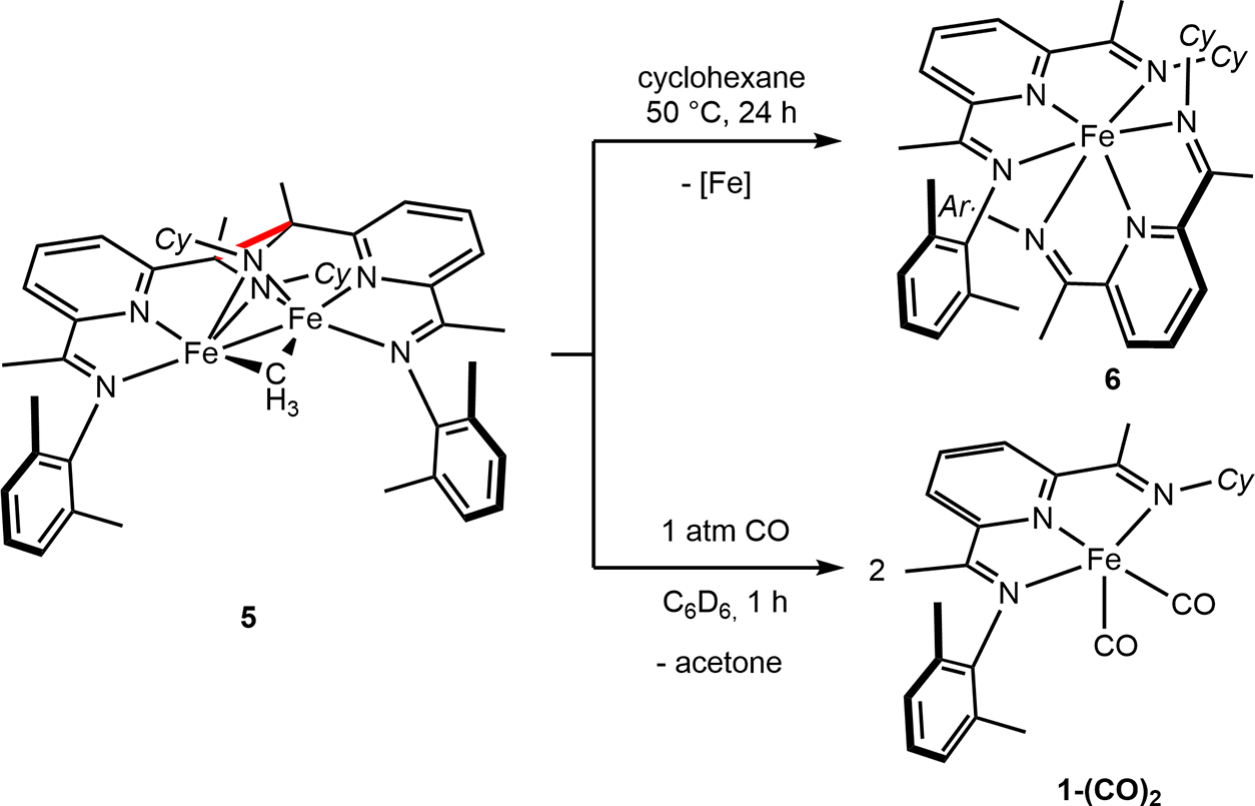
Stoichiometric Reactivity of **5**

The reversibility of the C–C bond-forming ligand dimerization was studied. Heating a cyclohexane solution of **5** at 50 °C resulted in the quantitative conversion to the bis(chelate)iron compound **6** along with a precipitate that is likely metallic iron or iron oxides (Scheme 7). Exposure of a benzene-*d*_6_ solution of **5** to one atmosphere of CO resulted in the rapid and quantitative conversion to (PDI)Fe(CO)_2_, demonstrating the reversibility of the ligand dimerization. The iron dicarbonyl complex was independently synthesized by sodium amalgam reduction of (PDI)FeCl_2_ in the presence of carbon monoxide (see the Supporting Information). Acetone was observed as a byproduct of the reaction, accounting for the methyl group and is likely formed from the initial CO insertion into the iron methyl bond, followed by a cross insertion into a second iron methyl bond.^44^ These results support a weak C–C bond between the chelates, a behavior that has been observed previously with metal complexes bearing redox-active ligands.^45−47^ However, the dinuclear iron complex **5** was unreactive toward olefins and was not involved in the transformation of butadiene and ethylene.

**Scheme 8:**
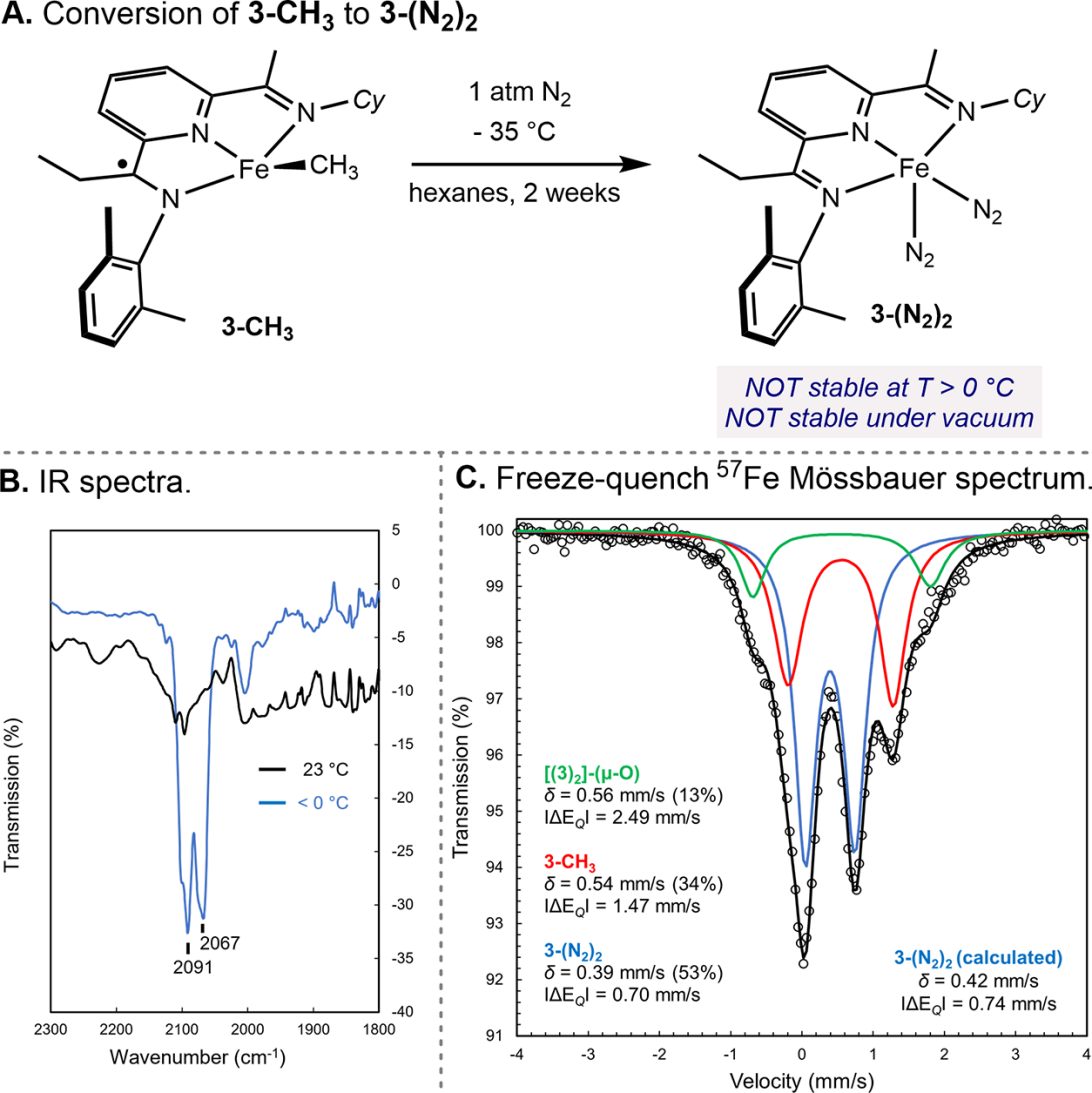
Formation, Characterization, and Thermal Stability of **3-(N**_**2**_**)**_**2**_

Changing one imine backbone substituent from methyl (**1-CH**_**3**_) to ethyl (**3-CH**_**3**_) significantly enhanced the solubility of the iron methyl complex. A solution of **3-CH**_**3**_ in hexane stored at −35 °C changed color from green to red over the course of 2 weeks (Scheme 8A). The resulting iron product was assigned as the dinitrogen complex, (^Cy^A^Me^(Me,Et)PDI)Fe(N_2_)_2_ (**3-(N**_**2**_**)**_**2**_), based on the observation of two diagnostic N–N stretches observed at 2091 and 2067 cm^–1^ in the hexane solution infrared spectrum recorded below 0 °C. Upon warming to 23 °C, these bands disappeared, signaling decomposition (Scheme 8B). Attempts to record ^1^H NMR spectra and obtain X-ray quality crystals have been unsuccessful due to thermal instability of the complex.

Freeze-quench ^57^Fe Mössbauer (80 K) spectroscopy was used to further support the identity of **3-(N**_**2**_**)**_**2**_ (Scheme 8C). Three overlapping quadrupole doublets were observed in a 13:34:53 ratio, with parameters δ = 0.56 mm/s and |Δ*E*_Q_| = 2.49 mm/s, δ = 0.54 mm/s and |Δ*E*_Q_| = 1.47 mm/s, and δ = 0.39 mm/s and |Δ*E*_Q_| = 0.70 mm/s. The minor signal is attributed to the decomposition and formation of the μ-oxo iron compound, **[3]**_**2**_**(μ-O)**, arising from the interaction of **3-CH**_**3**_ with trace amounts of water or oxygen. The solid-state structure of this compound was confirmed by X-ray diffraction (see Figure S19). The two major signals correspond to **3-CH**_**3**_ (δ = 0.54 mm/s) and **3-(N**_**2**_**)**_**2**_ (δ = 0.39 mm/s), with the latter exhibiting indistinguishable parameters from [(^Me^PDI)Fe(N_2_)]_2_(μ-N_2_) (δ = 0.37 mm/s; |Δ*E*_Q_| = 0.49 mm/s).^23^ The slightly larger quadrupole splitting of **3-(N**_**2**_**)**_**2**_ is attributed to the lower symmetry of the *C*_*S*_-symmetric ligand. The DFT-calculated ^57^Fe Mössbauer parameters δ = 0.42 mm/s and |Δ*E*_Q_| = 0.74 mm/s for **3-(N**_**2**_**)**_**2**_ are in good agreement with the experimental data and within the generally accepted error range.^48^ This result constitutes the first observation of a (PDI)Fe(N_2_) that does not bear two protective 2,6-disubstituted anilines on the tridentate chelate. However, this compound is thermally unstable as a result of the more electron-donating alkylated imine and provides important insights into the design of reduced iron precatalysts.

**Scheme 9:**
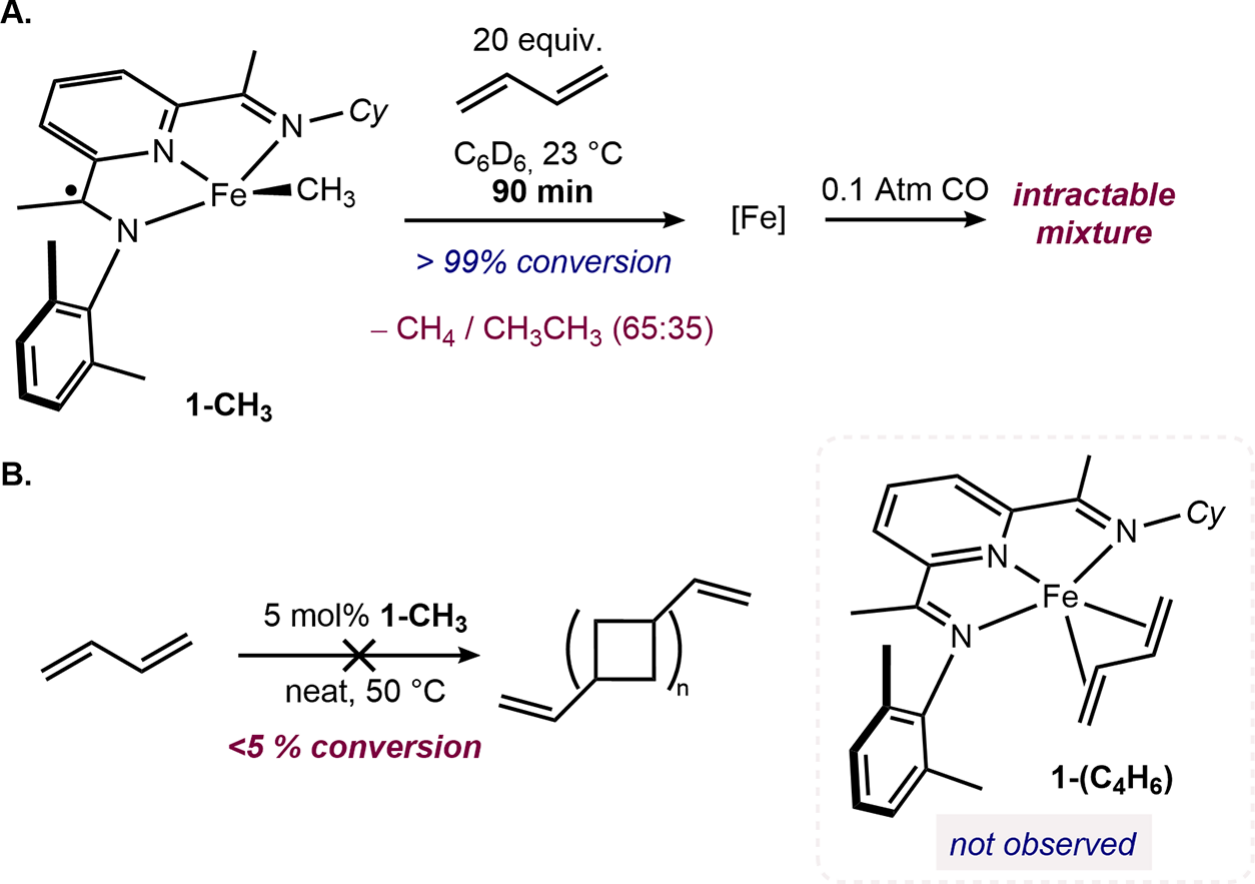
Stoichiometric Reactivity and Catalytic Activity of **1-CH**_**3**_ in the Presence of Butadiene

To further investigate the stoichiometric reactivity of **1-CH**_**3**_ and to explore the synthesis and stability of (PDI)Fe(s-*trans*-η^4^-C_4_H_6_), 20 equivalents of butadiene were added to a benzene-*d*_6_ solution of **1-CH**_**3**_ (Scheme 9). Full conversion of **1-CH**_**3**_ was observed after 90 min. Both ethane and methane were detected along with an intractable mixture of iron compounds. Adding 0.1 atmospheres of CO to the reaction mixture also produced an intractable mixture of iron compounds, supporting catalyst decomposition. No catalytic [2+2]-cycloaddition to yield divinyl(oligocyclobutane) was observed using 5 mol % of **1-CH**_**3**_ in neat butadiene at 50 °C. This result, in combination with the inability to prepare or observe **1-(C**_**4**_**H**_**6**_**)** from the reduction of **1-Cl**_**2**_ in the presence of butadiene, suggests that the iron butadiene compound is kinetically unstable, again highlighting the impact of imine alkylation on iron precatalyst stability.

**Figure 3:**
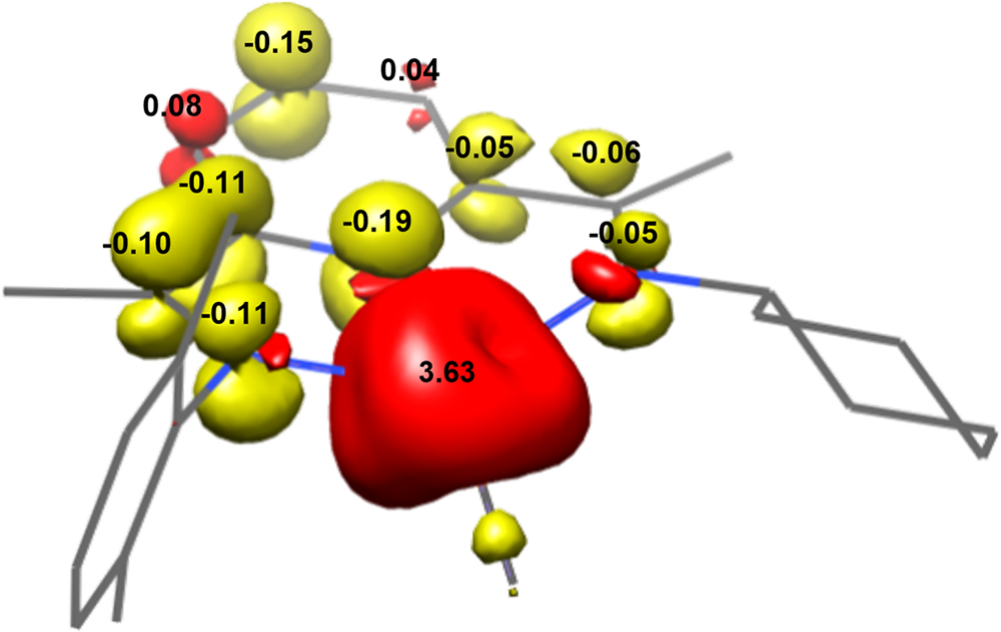
Calculated spin-density plot of **1-CH**_**3**_.

The stoichiometric reactivity observed with the C_*S*_-symmetric (PDI) iron methyl complex demonstrates the influence of changing one aniline to a cyclohexyl imine substituent of the (PDI) ligand as the corresponding formally iron(0) complexes are kinetically unstable. Understanding the impact and origin of these effects is critical for catalyst design and optimization. To gain additional insights into the relative thermal instability of the *C*_S_-symmetric iron(0) derivatives, DFT calculations (using the B3LYP functional with def2-SVP basis) were performed. The spin-density plot for **1-CH**_**3**_ exhibits delocalization of the antiferromagnetically coupled ligand radical preferred on the imine arm bearing the aniline (Figure 3). As a consequence, the introduction of a more σ-donating substituent on the (PDI) ligand results in increased reactivity at the (PDI) ligand at the imine bearing the cyclohexyl amine. A pathway involving deactivation of (PDI) iron catalysts by hydrogen transfer is also plausible.^49^ The cyclohexyl amine-substituted C_*S*_-symmetric (PDI) iron complexes presents an overall less protected and more electron-rich iron center compared to aryl(substituted) (PDI) iron complexes bearing 2,6-disubstituted anilines, decreasing the stability of formally iron(0) neutral derivatives.

### Precatalyst Activation under Catalytic Conditions and Identification of the Catalyst Resting States

**Scheme 10:**
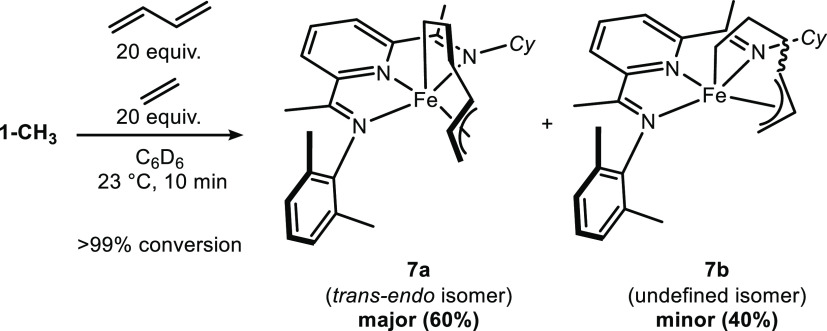
Precatalyst Activation and Identification of Catalyst Resting States

Significant insights were obtained on the relative instability of formally iron(0) complexes and for a metallacycle pathway to account for the observed hydrovinylation products. The mode of precatalyst activation and the identity of the iron resting state during the C–C bond formation were then of interest. Upon the addition of butadiene and ethylene to a benzene-*d*_6_ solution containing 5 mol % of **1-CH**_**3**_, a color change from deep green to orange was observed immediately upon mixing at ambient temperature (Scheme 10). Analysis of the products from precatalyst activation by ^1^H NMR spectroscopy revealed the formation of ethane and two diamagnetic iron compounds in a 60:40 ratio, consistent with a bimolecular reductive elimination of **1-CH**_**3**_.

Efforts were devoted to the identification of the two diamagnetic iron compounds **7a** and **7b** formed following the addition of substrates. Both compounds proved to be unstable in the absence of ethylene and butadiene as attempted isolation resulted in decomposition. Despite the diamagnetism, assignment of the ^1^H NMR spectra was challenging due to overlapping resonances arising from the low symmetry of the iron compounds. As a result, ^13^C and deuterium labeling were used to provide additional insights (see the SI). Use of ^13^C_2_H_4_ in the catalytic reaction produced four signals in the ^13^C NMR spectrum, corresponding to two ethylene subunits coordinated to two different iron compounds. In a second set of experiments, the ^13^C NMR spectra of two catalytic reactions performed with butadiene-*d*_0_ or butadiene-*d*_6_ were compared. Subtraction of the two spectra resulted in the assignment of eight signals for butadiene subunits coordinated to two different iron compounds, establishing the presence of butadiene in each compound. The ^1^H and ^13^C chemical shifts for major component **7a** are consistent with a *trans*-metallacycle as previously reported for (^Me^PDI)Fe(η^1^,η^3^-*trans*-C_6_H_10_). This compound was identified as the resting state in the catalytic [2+2]-cycloaddition of butadiene and ethylene.^23^

To gain additional information about the C–C connectivity between the ethylene and the butadiene subunits in **7b**, 2D NMR experiments were performed. The *in situ*^1^H–^13^C HMBC spectrum of the reaction with 99% ^13^C-enriched ethylene in toluene-*d*_8_ after 10 min at 23 °C was successfully obtained at 0 °C, avoiding signal quality loss due to butadiene and ethylene conversion over the course of the data acquisition. Signals for correlations between protons of the butadiene fragments and carbons of the ethylene fragments were observed for both the major (**7a**) and the minor (**7b**) iron(II) compounds. This result is consistent with **7a** and **7b** being two stereoisomeric metallacycles formed upon oxidative cyclization of butadiene and ethylene.

**Figure 4:**
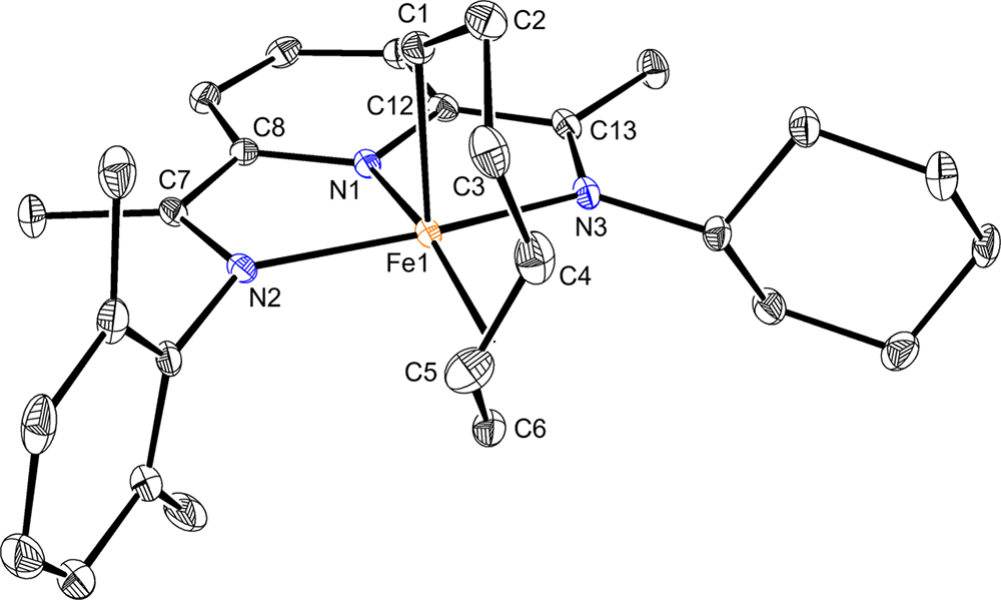
Representation of the solid-state structure of **7a** at 30% probability ellipsoids. Hydrogen atoms are omitted for clarity.

Crystals of **7a** suitable for X-ray diffraction analysis were obtained from a solution of **1-CH**_**3**_ in toluene-*d*_8_ briefly exposed to excess of butadiene and ethylene and then layered with hexanes at −35 °C. Similar to (^Me^PDI)Fe(η^1^,η^3^-*trans*-C_6_H_10_), **7a** is best described as a six-coordinate pyridine(dimine) iron(II) complex (Figure 4) Two features are noteworthy. First, the geometry of the metallacycle allyl fragment consists of a trans allyl with the central carbon pointing toward the aryl-imine “arm” of the (PDI) ligand and is designated as the *trans-endo* isomer. Second, the cyclohexyl imine substituent is positioned away from the coordination sphere of the iron in contrast to the orientation observed in the solid-state structure of **1-CH**_**3**_. This second feature illustrates the flexibility of the pyridine(diimine) in response to the steric environment of the hydrocarbon ligand coordinated to iron.

**Figure 5:**
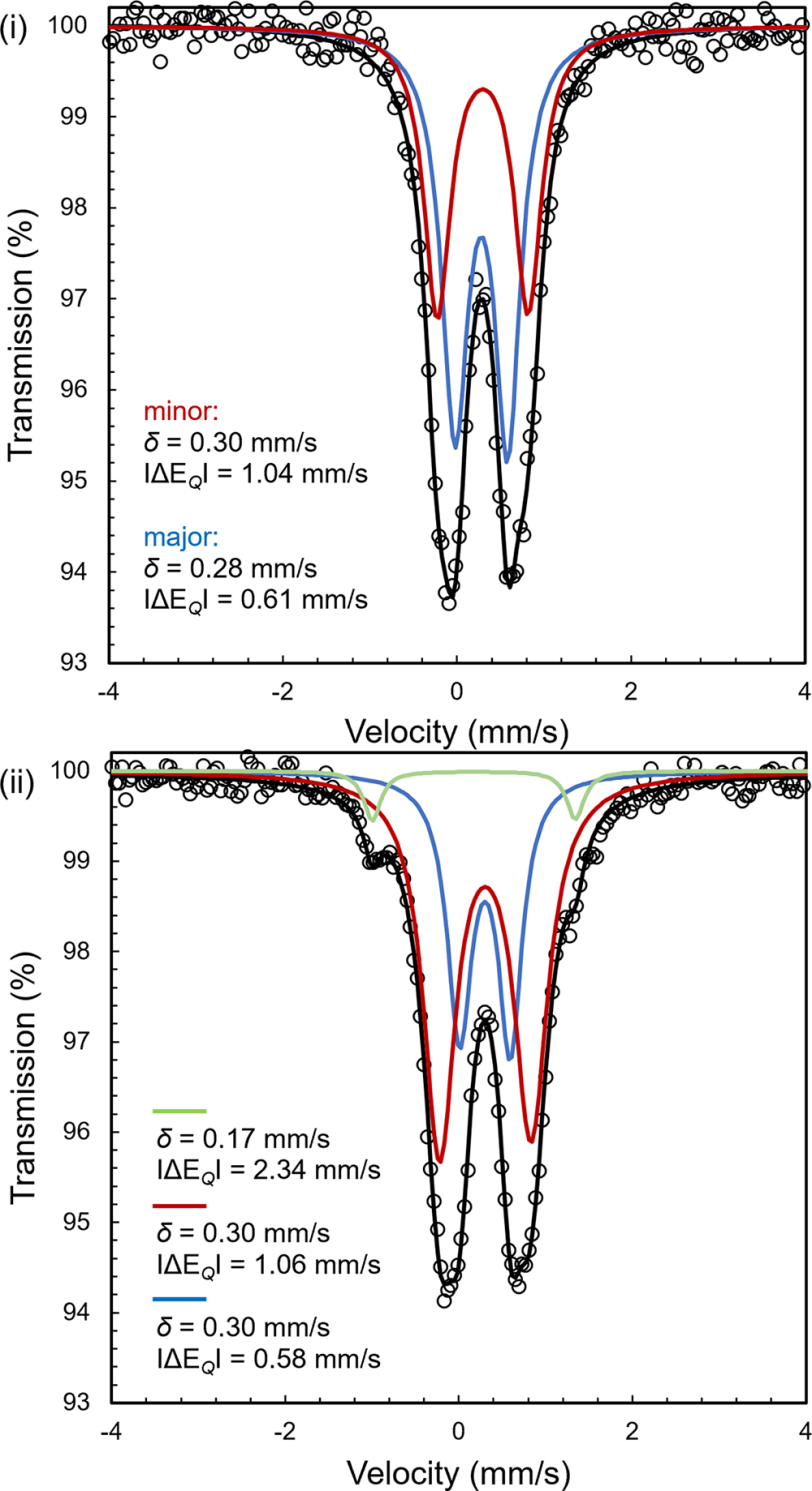
Freeze-quench ^57^Fe Mössbauer spectra (80 K) of the reaction of butadiene and ethylene with 25 mol % of **1-CH**_**3**_ after (i) 10 min and (ii) 60 min.

Freeze-quench ^57^Fe Mössbauer spectroscopy was also used to corroborate the identities of **7a** and **7b** and to provide additional insights into their electronic structures (Figure 5). In a typical experiment, a benzene solution containing 25 mol % of **1-CH**_**3**_ was treated with an equimolar amount of butadiene and ethylene. After approximately 10 min, the reaction mixture was frozen in liquid nitrogen and transferred to the instrument and the spectrum was recorded. Two overlapping doublets were observed in an approximately 60:40 ratio. The major component has parameters δ = 0.28 mm/s and |Δ*E*_Q_| = 0.61 mm/s, consistent with the values reported for the isolable metallacycle (^Me^PDI)Fe(C_6_H_10_) (δ = 0.27 mm/s and |Δ*E*_Q_| = 0.63 mm/s). This compound was assigned as the *trans-*metallacycle **7a**. The minor component **7b** exhibited parameters at δ = 0.30 mm/s and |Δ*E*_Q_| = 1.04 mm/s consistent with an iron(II) metallacycle and a stereoisomer of the major compound. After 60 min at 23 °C, the catalytic reaction performed with 25 mol % of **1-CH**_**3**_ resulted in catalyst decomposition as judged by ^1^H NMR spectroscopy. The freeze-quench ^57^Fe Mössbauer spectrum of this sample revealed 5% of a new signal with parameters at δ = 0.17 mm/s and |Δ*E*_Q_| = 2.34 mm/s, tentatively assigned as an iron hydride that is responsible for isomerization of (*Z*)-hexa-1,4-diene.

**Figure 6:**
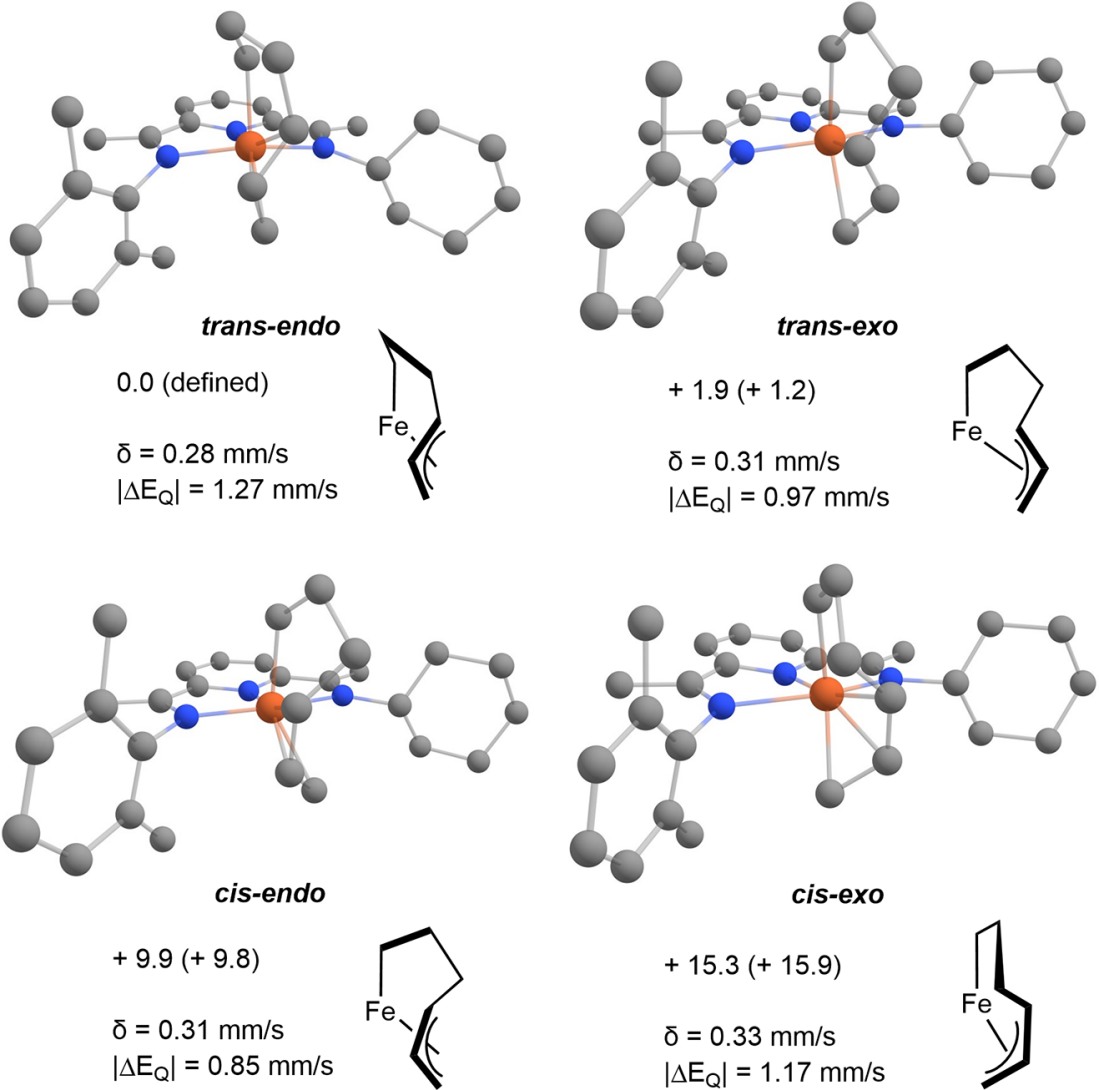
Optimized structures and calculated Mössbauer parameters for *trans* and *cis* isomers of (^Cy^A^Me^PDI)Fe(η^1^,η^3^-C_6_H_10_). Enantiomers are omitted for clarity. Relative free energy listed in kcal/mol calculated at 298 K. Relative electronic energies are listed in parentheses. Hydrogens are omitted for clarity.

Full-molecule DFT calculations were conducted to understand the different isomers of the metallacycle as well as to corroborate their Mössbauer parameters (Figure 6). Consistent with the observed major isomer and the X-ray diffraction analysis, the *trans-endo* isomer is the lowest in energy compared to the *trans-exo* (ΔG = 1.9 kcal/mol). By comparison, the *cis-endo* and *cis-exo*, involved in the hydrovinylation process, are higher in energy by ΔG = 9.9 and 15.3 kcal/mol, respectively, but are nevertheless thermally accessible. All of the calculated Mössbauer parameters for *trans*- and *cis*-metallacycles isomers span between δ = 0.28–0.33 mm/s and |Δ*E*_Q_| = 0.85–1.27 mm/s and are in good agreement with the experimental data obtained for **7a** and **7b**.^48^ The calculated Mössbauer parameters support the minor isomer **7b** as (PDI)Fe(η^1^,η^3^-C_6_H_10_) and an isomer of **7a**; however, the specific geometry assignment cannot be reliably assigned based on these data alone.

**Figure 7:**
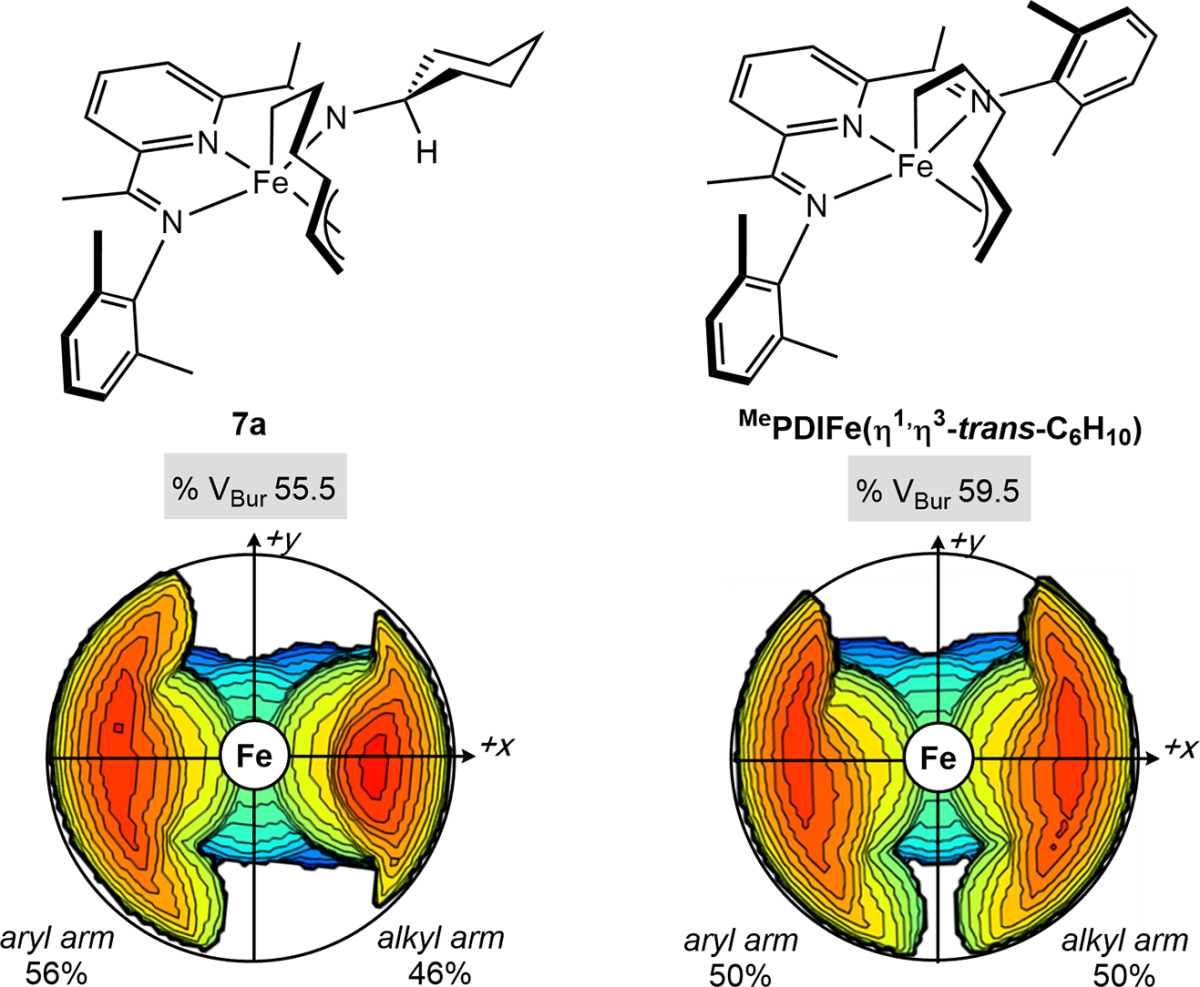
Steric maps and buried volume comparison between **7a** and (^Me^PDI)Fe(η^1^,η^3^-C_6_H_10_).

The orientation of the cyclohexyl group on the chelate was also established by ^1^H–^1^H NOESY NMR spectroscopy and found to be oriented away from the active site of the iron in both **7a** and **7b**. The calculated lowest-energy optimized structures for the *trans-endo* metallacycle isomer reproduced this experimental observation with a preference for the cyclohexyl orientation pointing away from the metallacycle with energy differences of ΔΔ*G* = 5.1 kcal/mol^–1^ (see the Supporting Information). Based on this finding, a comparison of the steric maps and the buried volumes^50−52^ of **7a** and (^Me^PDI)Fe(η^1^,η^3^-*trans*-C_6_H_10_) illustrate a more accessible iron center for the *C*_*S*_-symmetric (aryl,alkyl)-substituted (PDI) ligand (Figure 7).

Having established that the formations of both hydrocarbon products occur through metallacycle intermediates, mechanisms for their formation were considered based on experimental observations. It is likely that both products arise from a common intermediate, either an iron metallocycle or the corresponding ethylene butadiene complex. A Curtin–Hammett scenario is likely operative, where isomers of the metallocycle arising from the symmetry of the ligand interconvert and undergo subsequent reductive elimination or β-H elimination processes to account for the observed products. We note that the ratio of these products is temperature-dependent (see the Supporting Information).

### Metallacycle Solution-State Dynamics

**Scheme 11:**
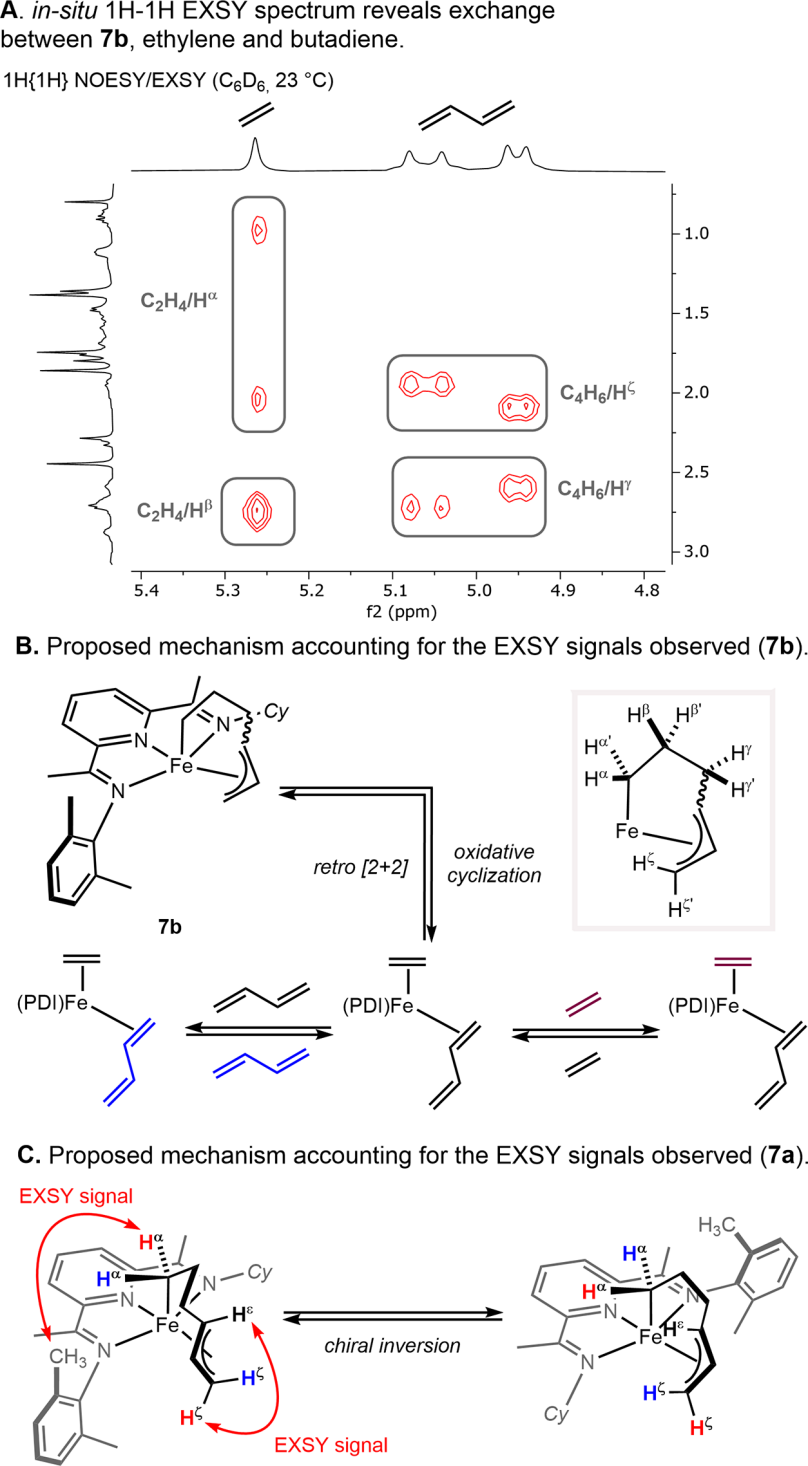
^1^H–^1^H EXSY NMR Spectrum of **7a** and **7b** and Proposed Mechanisms Accounting for the Observed Cross Peaks

The solution dynamics of both **7a** and **7b** were evaluated with variable-temperature NMR spectroscopy. The NMR studies were conducted following the addition of excess ethylene and butadiene to a toluene-*d*_8_ solution of **1-CH**_**3**_. The reaction was initially maintained at 23 °C for 5 min to allow the bimolecular reductive elimination from **1-CH**_**3**_ before being cooled to −80 °C in the NMR probe. Upon warming to −30 °C and measuring the ^1^H NMR spectrum every 10 °C, no detectable change in the ratio of the two isomers of the metallacycles was observed (see Figure S48). The complementary, *in situ* EXSY NMR spectrum of the resting state was recorded at 0 °C in toluene-*d*_8_ and at 23 °C in benzene-*d*_6_ (Scheme 11A). At both temperatures, no cross peaks between the two complexes were observed, establishing the lack of interconversion between **7a** and **7b** on the NMR time scale. Cross peaks were observed between the metallacycle protons of **7b** and butadiene and ethylene at 23 °C, establishing reversible oxidative cyclization (Scheme 11B). For metallacycle **7a**, at 0 and 23 °C, cross peaks between the aryl methyl substituent of the (PDI) ligand and H^α′^ and between H^δ^ and H^ζ′^ are consistent with inversion of the chirality at the iron. Plausible explanations accounting for an equilibrium between enantiomers are either reversibility of the oxidative addition such as for **7b** or reversibility of the reductive coupling (Scheme 11C).

### Determination of Deuterium Kinetic Isotopic Effects

**Scheme 12:**
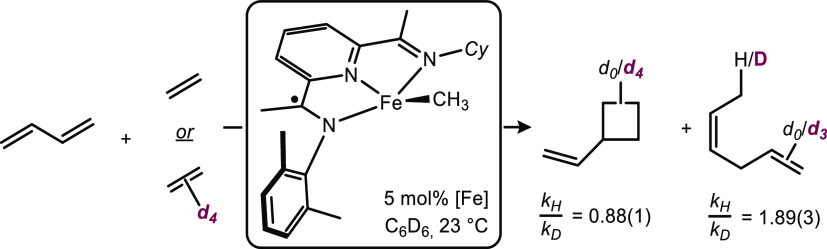
Determination of Deuterium Kinetic Isotope Effects for the [2+2]-Cycloaddition and the Hydrovinylation Pathway with **1-CH**_**3**_ as the Precatalyst

To gain information on the effect of the *C*_*S*_-symmetric ligand on the mechanism of the catalytic transformation of butadiene and ethylene, a series of isotopic labeling experiments were conducted (Scheme 12). Addition of a mixture of butadiene and ethylene to 5 mol % of **1-CH**_**3**_ in benzene-*d*_6_ produced a 51:49 mixture of vinylcyclobutane and (*Z*)-hexa-1,4-diene. Using ethylene-*d*_4_ in place of natural abundance ethylene, the ratio of products changed to 69:31 favoring vinylcyclobutane. This value supports a kinetic isotope effect of 2.0, with the C–H bond cleavage being rate-determining in the [1,4]-hydrovinylation reaction. More precisely, the rate of the two parallel reactions was measured at 25 °C and provided *k*_H_/*k*_D_ values of 0.88(1) and 1.89(3) for vinylcyclobutane and (*Z*)-hexa-1,4-diene, respectively. The KIE value for (*Z*)-hexa-1,4-diene is in the range corresponding to β-H elimination being the rate-determining step for the hydrovinylation process.^53^ The inverse KIE observed for vinylcyclobutane formation is proposed to arise either from oxidative cyclization being a rate-determining step or from an interdependence between the [2+2]-cycloaddition and the hydrovinylation catalytic cycles.

### Irreversibility of the Cyclobutane-Forming Reductive Elimination

**Scheme 13:**
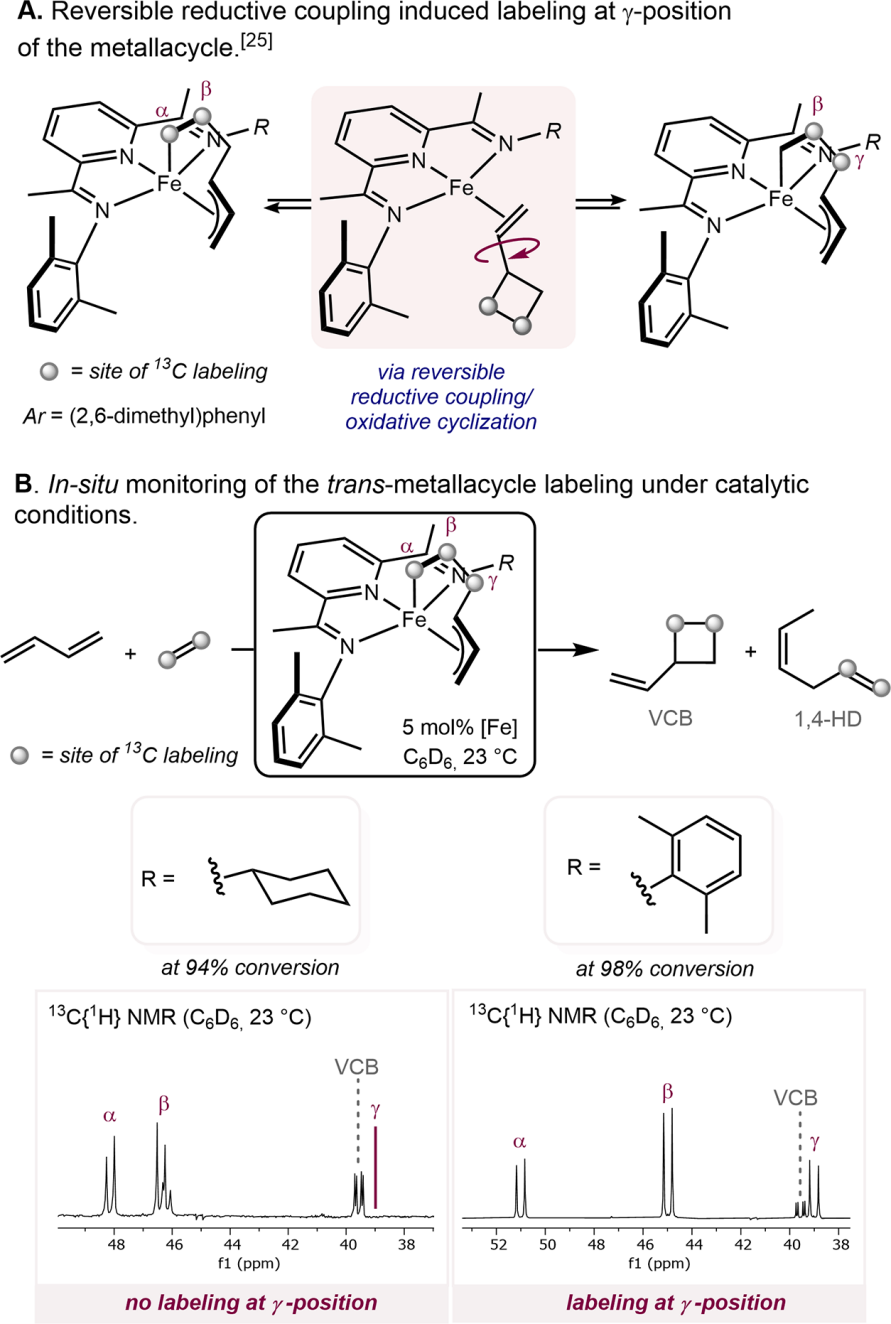
^13^C_2_H_4_ Labeling Experiment under Catalytic Conditions

A notable feature of the (bis)aryl-substituted (PDI)Fe-catalyzed [2+2]-cycloaddition to form vinylcyclobutane and divinyl(oligocyclobutane) is the reversibility of the reaction, enabling C–C bond breaking, and in the case of the oligomer, a chemically recyclable polyolefin. To probe the reversibility of the reductive coupling leading to the formation of the four-membered ring, a stoichiometric labeling experiment was designed.^25^ Addition of ^13^C-enriched ethylene to a solution of (^Me^PDI)Fe(s-*trans*-C_4_H_6_) resulted in labeling at the α, β, and γ positions of the formed (^Me^PDI)Fe(η^1^,η^3^-*trans*-C_6_H_10_) metallacycle (Scheme 13A). The *C*_S_-symmetric (^Cy^A^Me^PDI)Fe(η^1^,η^3^-*trans*-C_6_H_10_) **6a** was not isolable; therefore, the labeling experiment was conducted with *in situ* formation of the metallacycle under catalytic conditions. A control experiment was conducted using [(^Me^PDI)Fe(N_2_)]_2_(μ-N_2_) as a precatalyst to generate *in situ* the corresponding metallacycle. Labeling at the γ-position was detected at conversions higher than 80%, in agreement with the competitive vinylcyclobutane ligand exchange with free butadiene at high diene concentrations (Scheme 13B). Using **1-CH**_**3**_ as a precatalyst to form *in situ***7a**, no γ-position labeling on the metallacycle was observed even at high substrate conversion, demonstrating irreversible C–C bond formation.

### Mechanistic Distinctions in Catalytic C–C Bond Formation between *C*_S_- and *C*_2v_-Symmetric (PDI) Iron Precatalysts

The direct comparison between the (bis)aryl-substituted and the (aryl,alkyl)-substituted (PDI) iron catalyst informs on the effect of the ligand modification on both the stoichiometric reactivity and overall selectivity in catalytic C–C bond-forming reactions. The stoichiometric experiments established less stable, though accessible and observable, formally iron(0) complexes as compared to the isolable *C*_2V_-symmetric variants. Conversion of **1-CH**_3_ under one atmosphere of dinitrogen at room temperature led to a dinuclear iron complex with a bridging methyl complex formed upon dimerization of the (PDI) ligand at the imine carbon position. At −35 °C, a solution of **3-CH**_**3**_ in hexanes was converted to the corresponding iron dinitrogen **3-(N**_**2**_**)**_**2**_ complex. Computational studies support a spin density favoring the aniline-substituted portion of the ligand. The more σ-donating alkyl imine is likely less effective at stabilizing the antiferromagnetically coupled radical, therefore enabling (PDI) ligand reactivity and consequential catalyst degradation. These results highlight the role of redox-activity and noninnocence in supporting isolable, reduced iron precatalysts, as well as tuning the potential of the chelate to suppress unwanted ligand-based reactivity.

In the case of bis(arylated), *C*_2v_-symmetric pyridine(diimine) complexes, both the iron butadiene and metallacycle complexes are isolable. Introduction of an alkyl imine substituent decreases the stability of the corresponding butadiene complex, while the metallacycles are kinetically persistent at ambient temperature and isolable.

These metallacycles with the *C*_S_-symmetric ligand decomposed rapidly in solution at an ambient temperature when substoichiometric quantities of butadiene and ethylene were present. The *in situ* solution characterization of this metallacycle, supported by DFT calculations, established a cyclohexyl ring oriented distal from the metal, rendering a more open coordination sphere than with the *C*_2*V*_-symmetric arylated complexes. Complementary deuterium labeling experiments support a metallacycle pathway as responsible for the formation of (*Z*)-hexa-1,4-diene, with β-hydride elimination being the rate-determining step. Taken together, these experiments support an accessible *cis*-metallacycle, consistent with an increased σ-donating character and more accessible steric environment of the *C*_S_-symmetric (PDI) ligand favoring oxidative cyclization. Thus, to favor [2+2] cycloaddition reactivity, more hindered by less electron-donating ligands are preferred.

The features of the *C*_*S*_-symmetric (PDI) iron catalysts also influence the mechanism of the [2+2]-cycloaddition. No evidence for the formation of an (alkyl,aryl)-substituted (PDI)Fe(s-*trans*-η^4^-C_4_H_6_) complex was obtained. In contrast, for the (bis)aryl-substituted case, (^Me^PDI)Fe(s-*trans*-η^4^-C_4_H_6_) was observed as an on-cycle intermediate during the [2+2]-cycloaddition of butadiene and ethylene. This experimental observation is consistent with a fast and reversible oxidative cyclization induced by the more σ-donating (alkyl,aryl)-substituted (PDI) ligand. Moreover, substituting one N-aryl ring by a cyclohexyl amine on the (PDI) iron catalyst also influences the final C(sp^3^)–C(sp^3^) bond-forming step and has consequences in the design of iron catalysts for the synthesis of chemically recyclable polyolefins. ^13^C labeling experiments with isotopically enriched ethylene with the *C*_2v_-symmetric iron catalysts demonstrated reversible reductive coupling as isotopic labels were observed in *three* positions. This was not observed with the *C*_S_-symmetric catalyst, establishing irreversible C–C bond formation. This irreversibility of the reductive elimination is likely responsible for a lower stability observed of the (aryl,alkyl)-substituted (PDI) iron metallacycle.

**Scheme 14:**
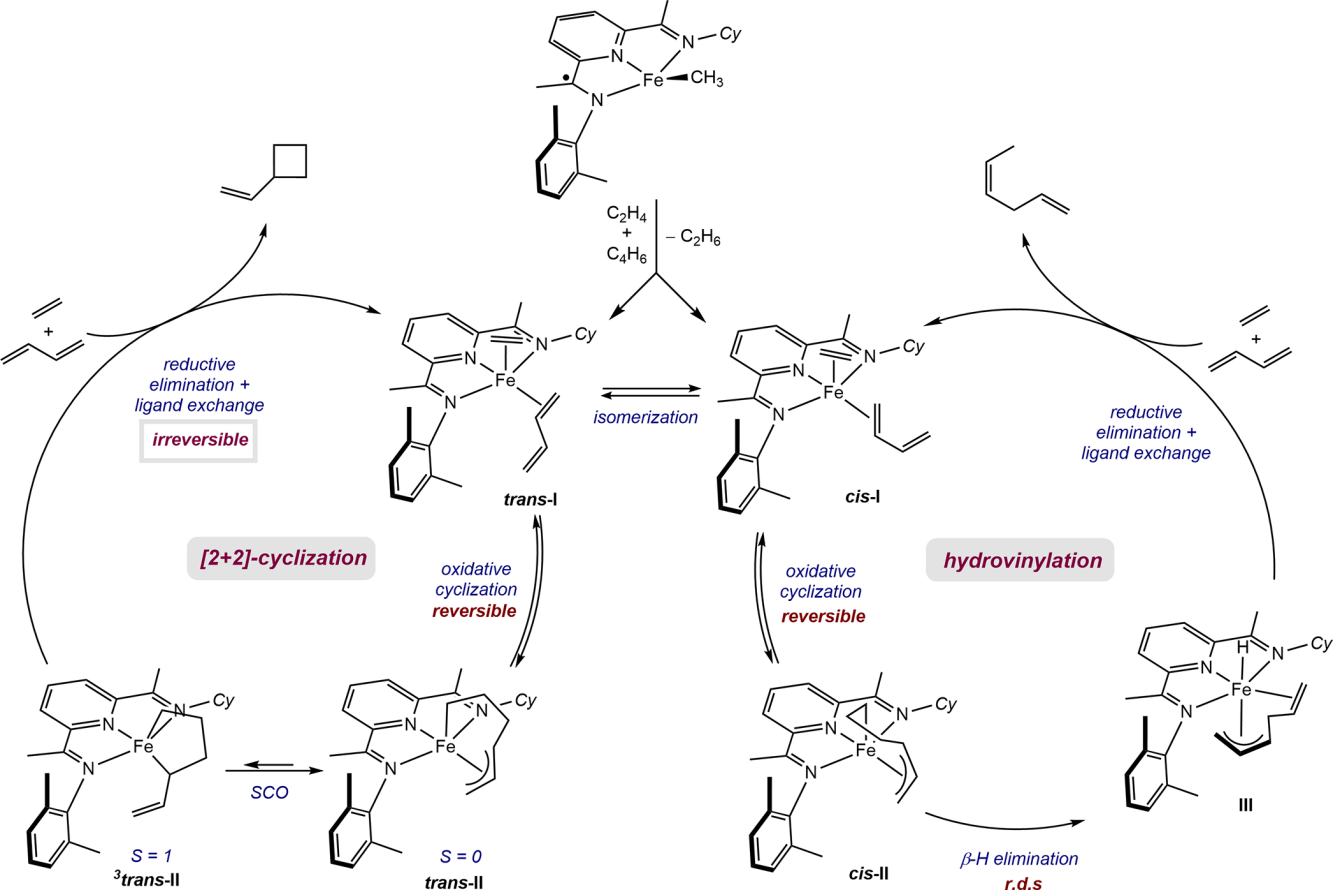
Proposed Catalytic Cycle for the Hydrovinylation of Butadiene and Ethylene with *C*_*S*_-Symmetric (PDI)FeCH_3_

With the combined resting state analysis, labeling experiments, and computational study, a catalytic cycle is proposed for the [2+2]-cycloaddition and hydrovinylation of butadiene and ethylene catalyzed with *C*_*S*_-symmetric pyridine(diimine) iron catalysts (Scheme 14). Activation of the iron methyl precatalyst occurs by bimolecular reductive elimination in the presence of ethylene and butadiene with the concomitant release of ethane. The resulting intermediate **I** with coordinated ethylene and butadiene undergoes fast and reversible oxidative cyclization to form a mixture of metallacycle isomers **II**. Previous work from our laboratory has demonstrated the importance of spin cross-over to access the triplet metallacycle responsible for accelerating C(sp^3^)–C(sp^3^) formation.^25^ The *trans*-isomers ***trans*****-II** undergo vinylcyclobutane-forming reductive elimination that is irreversible. In parallel, *C*_*S*_-symmetric (PDI) ligand enables thermally accessible *cis*-metallacycles ***cis*****-II** that are on cycle for catalytic butadiene hydrovinylation, with β-H elimination being the rate-determining step. Based on these data alone, an alternative mechanism involving isomerization of an allyl intermediate after β-hydride elimination from ***trans-*****II**, leading to the *cis*-hexadiene, cannot be excluded. Additionally, distinguishing between a cyclobutane-forming step occurring on a ground-state ***trans*****-II** metallacycle or on a triplet surface is challenging to discern and is currently under investigation. The equimolar ratio of vinylcyclobutane and hexadiene likely arises from similar rates of elimination from each metallacycle isomer, ***trans-*****II** and ***cis-*****II**, under a Curtin–Hammett scenario. More open and desymmetrized *C*_*S*_-symmetric iron catalysts likely facilitate the β-hydride elimination, which arise from more kinetically favorable imine dissociation due to enhanced *trans*-effect of the N-alkyl substituent.

## Conclusions

Mixed (alkyl,aryl)-substituted, *C*_*S*_-symmetric (PDI) iron methyl precatalysts have been synthesized and characterized. Previously, only *C*_2V_-symmetric (PDI) iron complexes bearing 2,6-disubstituted anilines were known to support catalytically active iron(0) complexes for C–C bond formation. Bimolecular reductive elimination of ethane from iron methyl complexes provided unique access to iron complexes that were catalytically active for the [2+2]-cycloaddition of butadiene and ethylene. However, substituting one of the N-imine aniline groups for an alkyl amine had profound effects on catalyst stability and selectivity. The formally iron(0) dinitrogen and butadiene derivatives proved less thermally stable and have resisted isolation, likely due to a more electron-rich and sterically open pyridine(diimine) chelate. In catalytic C–C bond-forming chemistry with butadiene and ethylene, hydrovinylation to form (*Z*)-hexa-1,4-diene was also observed in addition to the [2+2]-cycloaddition product. A combination of stoichiometric experiments, resting state analysis deuterium and ^13^C labeling experiments provided insights into the origins of the altered chemoselectivity. The decreased stability of the formally iron(0) compounds due to ligand reactivity coupled with access to a stereoisomeric *cis*-metallacycle and the irreversibility of cyclobutane-forming reductive elimination work in concert to enable the formation of acyclic products. The knowledge gained from these studies provides fundamental insights into the unique selectivity of the pyridine(diimine)-substituted precatalysts for catalytic C–C bond-forming chemistry with feedstock alkenes and dienes, a consequence of a balance of steric environment and redox-activity.

## Methods

### General Procedure for the Synthesis of (PDI)FeCH_3_

In a glovebox, a 20 mL scintillation vial was charged with 1.0 equiv of (PDI)FeCl_2_ and approximately 15 mL of diethyl ether. The slurry was frozen in a cold well, and MeLi (1.5M in diethyl ether, 2.0 equiv) was added. The solution was stirred as it was warmed to room temperature. After stirring for 30 min, the solution was filtered through celite and washed with diethyl ether and the volatiles were removed in vacuo. The crude product was recrystallized at −35 °C overnight to afford a pure sample of (PDI)FeCH_3_ as a dark green solid or used as the crude material.

### General Procedure for the Fe-Catalyzed [2+2]-Cycloaddition/Hydrovinylation of Ethylene and Butadiene

In a nitrogen-filled glovebox, a solution of [Fe] (0.022 mmol) in 500 mg of benzene-*d*_6_ was transferred to a J. Young tube. The tube was sealed and removed from the glovebox, and the contexts were frozen in liquid dinitrogen. The head-space was evacuated, and butadiene (0.44 mmol), followed by ethylene (0.44 mmol), was added by vacuum transfer via a calibrated bulb. The tube was sealed under static vacuum, and contents were thawed and mixed by inversion at room temperature while the reaction progress was monitored by ^1^H NMR spectroscopy. The volatiles of the reaction were transferred to another J. Young tube and then analyzed by ^1^H and ^13^C{1H} NMR spectroscopies. The products of the reaction, vinylcyclobutane (VCB) and (Z)-hexa-1,4-diene (1,4-HD) and hexa-2,6-diene isomers, are known in the literature, and the NMR spectra were compared to commercial samples or previously reported data.

## Supporting Information Available

General considerations; synthesis and characterization of (PDI) ligands and iron complexes; iron-catalyzed [2+2]-cycloaddition/hydrovinylation of ethylene and butadiene; in situ characterization of the iron metallacycles; labeling experiments; and computational analyses (PDF)
Crystallographic data (CIF)

## Supplementary Material

au3c00229_si_001.pdf

au3c00229_si_002.cif
