# Supplementary material for: CS-Symmetric Pyridine(diimine) Iron Methyl Complexes for Catalytic [2+2] Cycloaddition and Hydrovinylation: Metallacycle Geometry Determines Selectivity
Source: JACS Au. 2023 Jul 12;3(7):2007–24. doi: 10.1021/jacsau.3c00229 (PMC10369671; doi:10.1021/jacsau.3c00229)
Supplement: Supplementary file 1 — au3c00229_si_001.pdf [file au3c00229_si_001.pdf]

Supporting Information

C_S-symmetric Pyridine(dimine) Iron Methyl Complexes for Catalytic [2+2] Cycloaddition and Hydrovinylation : Metallacycle Geometry Determines Selectivity

*Coralie Duchemin, Junho Kim, and Paul J. Chirik**

Department of Chemistry, Frick Laboratory
Princeton University, Princeton, NJ 08544, USA

pchirik@princeton.edu

Table of Content.

I. General Considerations S3

II. Synthesis and Characterization of (PDI) ligands and Iron Complexes S4
- i. Preparation of C_S-symmetric (PDI)$FeCl_2$ S4
- ii. Preparation of C_S-symmetric (PDI)$FeCH_3$ S14
- iii. Preparation of Dimeric Dinuclear Bridging Methyl Complex **5** S26
- iv. Preparation of ($^{Cy}A^{Me}$PDI)$Fe(CO)_2$ **1-(CO)$_2$** S30
- v. Preparation of ($^{Cy}A^{Me}$PDI)$_2$Fe **6** S34
- vi. Preparation of ($^{Cy}A^{Me}$(Me/Et)PDI)$Fe(N_2)_2$ **3-(N$_2$)$_2$** S37

III. Iron-catalyzed [2+2]-cycloaddition/hydrovinylation of ethylene and butadiene S39
- i. Extended Optimization Table S39
- ii. Time-course of the Reaction with **1-CH$_3$** and **4-CH$_3$** S40

IV. In-situ Characterization of the Iron Metallacycles S41
- i. NMR Experiments S41
- ii. Mössbauer Spectroscopy S48
- iii. X-Ray diffraction analysis for **7a** S49
- iv. Variable Temperature ^{1}H NMR Spectroscopy S51

V. Labeling Experiments S53
- i. Cross-over Experiment with Butadiene and Ethylene/Ethylene-d_4 S53
- ii. H/D Kinetic Isotopic Effect Measurement S57

iii. (PDI) to Substrates H/D Scrambling Evidence S58
iv. ^{13}C-labeled Ethylene Experiment Relevant to Reductive Elimination S60

VII. Computational Analyses S63
i. Computational Details S63
ii. ($^{Cy}A^{Me}$PDI)$FeCH_3$ (**1-CH_3**) S64
iii. ($^{Cy}A^{Me}$(Me/Et)PDI)$Fe(N_2)_2$ ((**3-N_2)$_2$**) S68
iv. ($^{Cy}A^{Me}$PDI)Fe(η^1,η^3-C_6H_{10}) S70

I. General Considerations

All air- and moisture-sensitive manipulations were carried out using vacuum line, Schlenk and cannula techniques or in an MBraun inert atmosphere (nitrogen) dry box unless otherwise noted. All glassware was stored in a pre-heated oven prior to use. The solvents used for air- and moisture-sensitive manipulations were dried and deoxygenated using literature procedures.[i] Butadiene, butadiene-*d6*, ethylene, ethylene-d_6 and ^{13}C-labeled ethylene were purchased in reagent grade from either Matheson or Aldrich. The butadiene was dry and deoxygenated by stirring vigorously with n-BuLi below 0 °C for 10 minutes, then vacuum transferred into a thick-walled glass vessel containing 4 Å molecular sieves. Ethylene was stored over activated 4 Å molecular sieves for at least 24 hours before use. Labelled gases were stored in thick-walled glass vessels and used as received. The following compounds were prepared according to literature procedures: vinylcyclobutane,[ii] $^{Cy}A^{Me}PDIFeCl_2$,[iii] $^{Cy}A^{iPr}PDIFeCl_2$,[iii] $[(^{Me}PDI)Fe(N_2)]_2(\mu\text{-}N_2)$.[iv]

^{1}H NMR spectra were recorded on either Bruker ADVANCE 400 or 500 spectrophotometers operating at 399.80 MHz, and 500.46 MHz, respectively. ^{13}C NMR spectra were recorded on either Bruker Avance 400 or 500 spectrometer operating at 100.54 MHz and 125.85 MHz, respectively. All ^{1}H and ^{13}C NMR chemical shifts are reported in ppm relative to $SiMe_4$ using the ^{1}H (chloroform-*d*: 7.26 ppm; benzene-d_6: 7.16 ppm; toluene-d_8: 7.00 ppm; cyclohexane-d_{12}: 1.38 ppm) and ^{13}C (chloroform-*d*: 77.16 ppm; benzene-d_6: 128.06 ppm; toluene-d_8: 137.48ppm) chemical shifts of the solvent as a standard. ^{1}H NMR data for diamagnetic compounds are reported as follows: chemical shift, multiplicity (s = singlet, d = doublet, t = triplet, q = quartet, p = pentet, br = broad, m = multiplet, app = apparent, obsc = obscured), coupling constants (Hz), integration, assignment. ^{1}H NMR data for paramagnetic compounds are reported as follows: chemical shift, integration, peak width at half height (Hz). ^{13}C NMR data for diamagnetic compounds are reported as follows: chemical shift, number of protons attached to carbon (e.g. CH_2), assignment.

Zero-field ^{57}Fe Mössbauer spectra were recorded on a SEE Co. Mössbauer spectrometer (MS4) at 80 K in constant acceleration mode. ^{57}Co/Rh was used as the radiation source. WMOSS software[v] was used for the quantitative evaluation of the spectral parameters (least-squares fitting to Lorentzian peaks). The temperature of the sample was controlled by a Janis Research Co. CCS-850 He/N_2 cryostat within an accuracy of 0.3 K. Isomer shifts were determined relative to α-iron at 298 K.

High-resolution mass spectra were measured using an Agilent 5975C GC-MS at the Princeton University Mass Spectrometry Facility.

Elemental analyses were performed at Robinson Microlit Laboratories, Inc., in Ledgewood, NJ. Solid-state magnetic moments were determined using a Johnson Matthey Magnetic Susceptibility Balance that was calibrated with $HgCo(SCN)_4$. High-resolution mass spectra were obtained at Princeton University mass spectrometry facilities using an Agilent 6210 TOF LC/MS. Infrared spectroscopy was conducted on a Thermo-Nicolet iS10 FT-IR spectrometer calibrated with a polystyrene standard.

Single crystals suitable for X-ray diffraction were coated with polyisobutylene oil in a drybox, transferred to a nylon loop and then quickly transferred to the goniometer head of a Bruker SMART APEX DUO diffractometer equipped with a molybdenum X-ray tube (λ = 0.71073 Å) and a Cu X-ray tube (λ = 1.54178 Å). Preliminary data revealed the crystal system. The data collection strategy was optimized for completeness and redundancy using the Bruker COSMO software suite. The space group was identified, and the data were processed using the Bruker SAINT+ program and corrected for absorption using SADABS. The structures were solved using direct methods (SHELXS) completed by subsequent Fourier synthesis and refined by full-matrix least-squares procedures.

Details for the DFT calculations are given in the section VII. Computational Analysis.

II. Synthesis and Characterization of (PDI) ligands and Iron Complexes

i. C_S-symmetric (PDI)$FeCl_2$ Complexes.

Characterization of C_S-symmetric (PDI)$FeCl_2$ (2-Cl_2) by X-ray Diffraction Analysis.

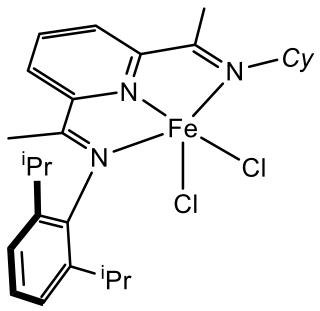

2-Cl_2 was prepared following the reported procedure from Bianchini and coworkers.[iii] Recrystallization by slow diffusion of pentane to a dichloromethane solution of **2-Cl_2** over four days at room temperature afforded suitable crystals for single-crystal X-ray diffraction analysis. The structure and coordinates were deposited with the Cambridge Crystallographic Data Center (CCDC# 2256982).

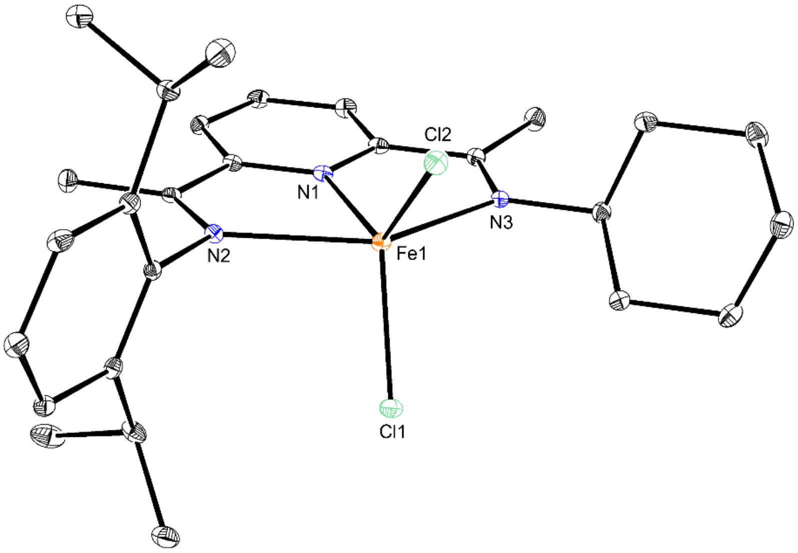

Figure S1. solid-state structure of **2-Cl_2**, depicted with **30**% probability ellipsoids (CCDC #2256982). Hydrogen atoms are omitted for clarity. C = gray, N = blue, Fe = red-orange, Cl = green.

Table S1: Experimental crystal data for **2-Cl_2**.

Crystal data	
Chemical formula	$C_{14}H_{19}Cl_2Fe_{0.50}N_{1.50}$
M_r	307.13

Crystal system, space group	Orthorhombic, $P2_12_12_1$
Temperature (K)	100
a, b, c (Å)	10.567 (4), 16.349 (5), 16.708 (5)
V (Å^3)	2886.3 (16)
Z	8
Radiation type	Mo $K\alpha$
μ (mm^{-1})	0.92
Crystal size (mm)	0.31 × 0.30 × 0.29
Data collection	
Diffractometer	Bruker *APEX*-II CCD
Absorption correction	Multi-scan<br>TWINABS BRUKER AXS
T_{min}, T_{max}	0.631, 0.746
No. of measured, independent and observed [$I > 2\sigma(I)$] reflections	21175, 7449, 6380
R_{int}	0.052
(sin θ/λ)$_{max}$ (Å^{-1})	0.676
Refinement	
$R[F^2 > 2\sigma(F^2)]$, $wR(F^2)$, S	0.036, 0.085, 1.02
No. of reflections	7449
No. of parameters	331
H-atom treatment	H-atom parameters constrained
$\Delta\rangle_{max}$, $\Delta\rangle_{min}$ (e Å^{-3})	0.68, -0.42
Absolute structure	Flack x determined using 2403 quotients [(I+)-(I-)]/[(I+)+(I-)] (Parsons, Flack and Wagner, Acta Cryst. B69 (2013) 249-259).
Absolute structure parameter	0.003 (10)

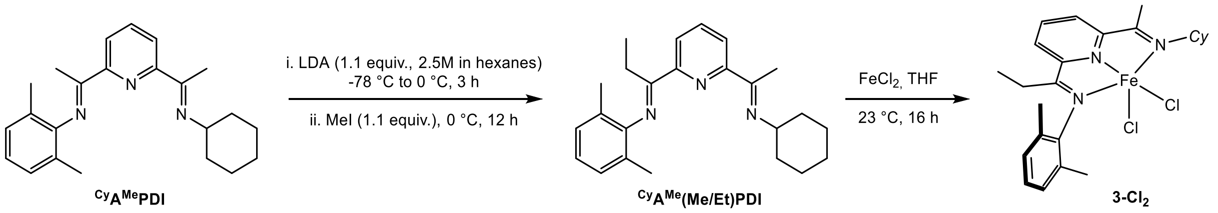

$^{Cy}A^{Me}$PDI **$^{Cy}A^{Me}$(Me/Et)PDI** **3-Cl$_2$**

Preparation of C_S-symmetric (PDI)$FeCl_2$ (3-Cl_2). In a 100ml two-necked round-bottom flask, nBuLi (1.1 equiv., 1.58 mmol, 2.5M in hexanes) was added dropwise at -78 °C to a solution of diisopropylamine (1.1 equiv., 160.16 mg, 1.58 mmol, 223.06 μL) in dry THF (10 ml) under argon. The solution was stirred for 30min, followed by slow addition of a solution of pyridine(diiimine) **$^{Cy}A^{Me}$PDI** (0.5 g, 1.44 mmol) in dry THF (10 ml). The reaction vessel was warm up to 0 °C and stirred for 3 h. At 0 °C, methyl iodide (224.66 mg, 1.58 mmol, 98.53 μL) was added dropwise and the reaction was run for 12 h at room temperature. The reaction mixture was then quenched with a saturated solution of $NaHCO_3$ and extracted with ethyl acetate (3 x 50 ml). The organic fractions were combined, dried over $MgSO_4$ and concentrated under vacuum to give the crude desired **$^{Cy}A^{Me}$(Me/Et)PDI** (525 mg, >99% yield)) as a light yellow oil. The crude material was dried under vacuum overnight and used without further purification in the next step.

1H NMR (500 MHz, chloroform-*d*, 20 ºC): δ 8.30 (d, *J* = 7.7 Hz, 1H), 8.19 (d, *J* = 7.7 Hz, 1H), 7.80 (t, *J* = 7.8 Hz, 1H), 7.07 (d, *J* = 7.6 Hz, 2H), 6.94 (t, *J* = 7.5 Hz, 1H), 3.60 (td, *J* = 10.2, 5.1 Hz, 1H), 2.70 (q, *J* = 7.6 Hz, 2H), 2.42 (s, 3H), 2.06 (s, 6H), 1.86 (dt, *J* = 13.1, 3.7 Hz, 2H), 1.79 – 1.64 (m, 4H), 1.62 – 1.51 (m, 3H), 1.49 – 1.28 (m, 4H), 1.03 (t, *J* = 7.6 Hz, 3H).

^{13}C NMR (126 MHz, chloroform-*d*, 20 ºC) δ 171.79, 163.88, 157.20, 154.14, 148.68, 136.88, 127.98 (2C), 125.54, 122.93, 122.16, 122.01, 60.39, 33.62 (2C), 25.96 (2C), 24.94 (2C), 23.45, 18.30 (2C), 13.59, 11.42.

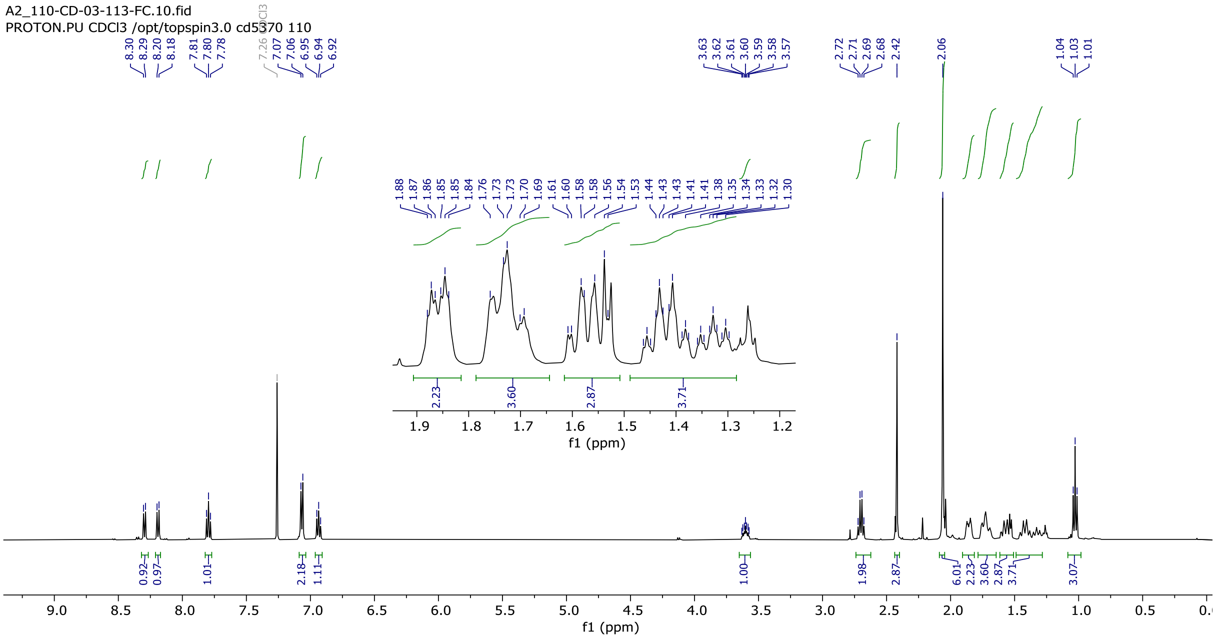

Figure S2. ^{1}H NMR (500 MHz, $CDCl_3$) spectrum of **$^{Cy}A^{Me}$(Me/Et)PDI**.

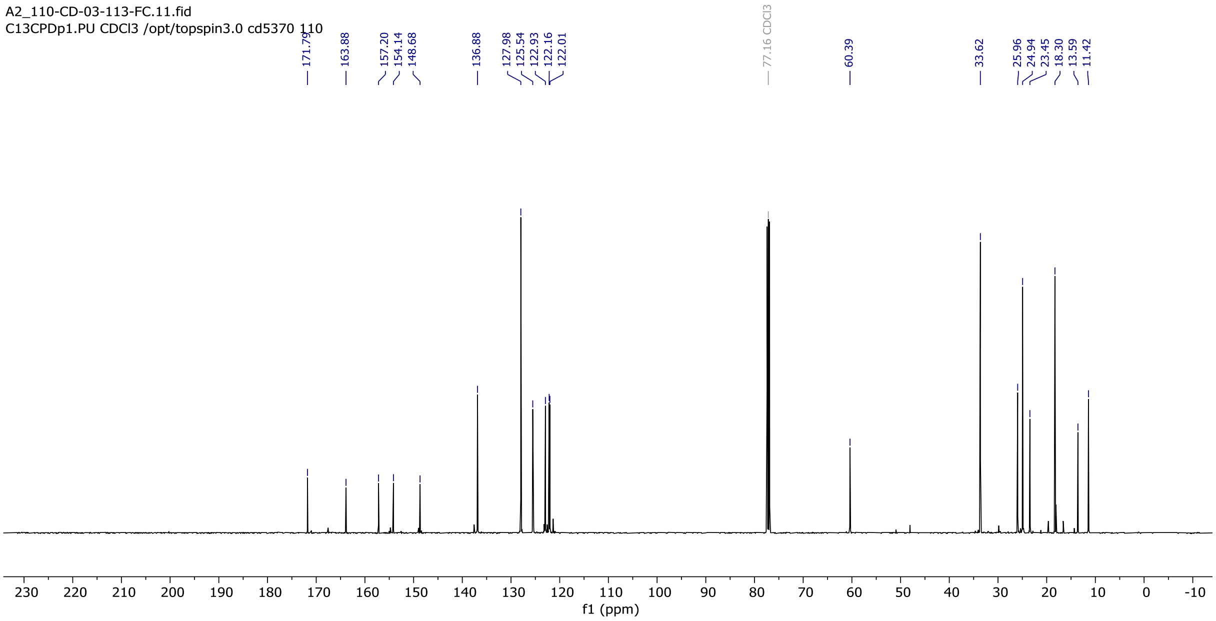

Figure S3. ^{13}C NMR (126 MHz, $CDCl_3$) spectrum of **$^{Cy}A^{Me}$(Me/Et)PDI**.

In a nitrogen-filled glovebox, iron(II) dichloride (178.67 mg, 1.41 mmol, 0.98 equiv.) was added to a solution of **$^{Cy}A^{Me}$(Me/Et)PDI** (520 mg, 1.44 mmol, 1.00 equiv.) in dry THF (20 ml) and the reaction was stirred overnight. The resulting precipitate was then filtered off and rinsed with a 1:1 mixture of THF and Et_2O and dried over vacuum to give **3-Cl_2** as a blue

powder (550 mg, 1.13 mmol, 78% yield). Recrystallization by slow diffusion of pentane to a dichloromethane solution of **3-Cl₂** over four days at room temperature afforded suitable crystals for single-crystal X-ray diffraction analysis. The structure and coordinates were deposited with the Cambridge Crystallographic Data Center (CCDC#2256989).

1H NMR (400 MHz, dichloromethane-*d₂*, 20 ºC): δ 200.54 (181 Hz), 87.62 (104 Hz), 71.95 (77 Hz), 20.63 (49 Hz), 19.15 (57 Hz), 14.72 (75 Hz), 12.18 (131 Hz), -0.80 (54 Hz), -2.00 (63 Hz), -3.38 (41 Hz), -4.11 (45 Hz), -5.23 (52 Hz), -5.46 (66 Hz), -8.13 (45 Hz), -22.67 (133 Hz), -26.74 (292 Hz).

Analysis for $C_{24}H_{31}Cl_2FeN_3$: Calc. C, 59.04; H, 6.40; N, 8.61. Found: C, 58.74 ; H, 6.44; N, 8.38.

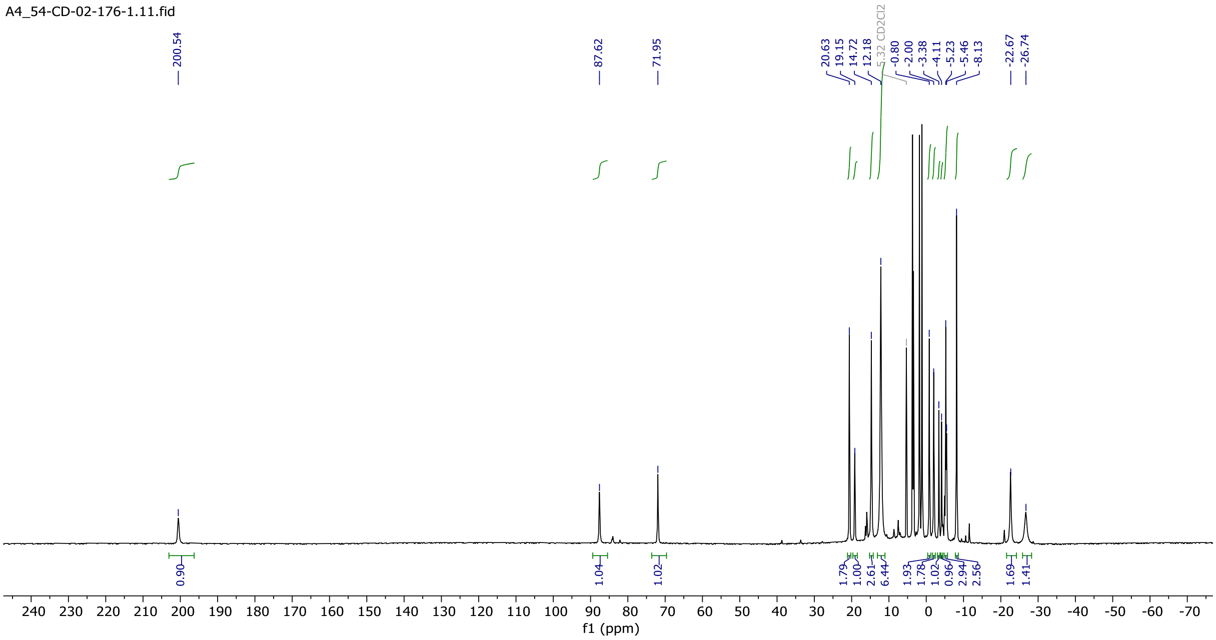

Figure S4. ¹H NMR (400 MHz, CD_2Cl_2) spectrum of **3-Cl₂**.

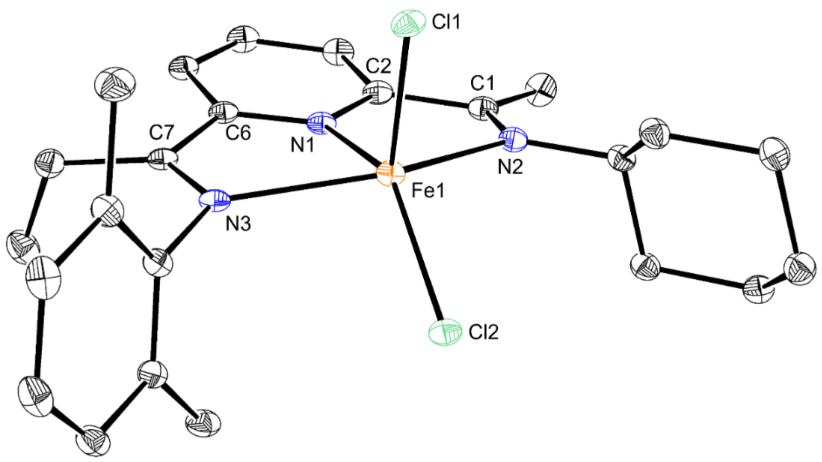

Figure S5. solid-state structure of **3-Cl₂**, depicted with **30**% probability ellipsoids (CCDC #2256989). Hydrogen atoms are omitted for clarity. C = gray, N = blue, Fe = red-orange, Cl = green.

Table S2: Experimental crystal data for **3-Cl₂**.

Crystal data

$C_{48}H_{62}Cl_4Fe_2N_6$	$F(000) = 1024$
$M_r = 976.53$	$D_x = 1.236$ Mg m^{-3}
Monoclinic, $P2_1/n$	Cu $K\alpha$ radiation, $\lambda = 1.54178$ Å
$a = 13.914$ (3) Å	Cell parameters from 9962 reflections
$b = 8.7239$ (10) Å	$\theta = 4.0–66.6°$
$c = 21.752$ (3) Å	$\mu = 6.58$ mm^{-1}
$\beta = 96.464$ (12)°	$T = 100$ K
$V = 2623.6$ (7) Å^3	Plate, dark blue
$Z = 2$	0.18 × 0.06 × 0.02 mm

Data collection

Bruker APEX-II CCD diffractometer	4289 reflections with $I > 2\sigma(I)$
ϕ and ω scans	$R_{int} = 0.043$
Absorption correction: multi-scan TWINABS BRUKER AXS	$\theta_{max} = 66.8°$, $\theta_{min} = 3.6°$
$T_{min} = 0.552$, $T_{max} = 0.753$	$h = -16\rightarrow16$
37602 measured reflections	$k = -10\rightarrow10$
4659 independent reflections	$l = -25\rightarrow24$

Refinement

Refinement on F^2	0 restraints
Least-squares matrix: full	Hydrogen site location: inferred from neighbouring sites
$R[F^2 > 2\sigma(F^2)] = 0.033$	H-atom parameters constrained
$wR(F^2) = 0.088$	$w = 1/[\sigma^2(F_o^2) + (0.039P)^2 + 2.5108P]$ where $P = (F_o^2 + 2F_c^2)/3$
$S = 1.08$	$(\Delta/\sigma)_{max} = 0.001$
4659 reflections	$\Delta\rangle_{max} = 0.61$ e Å^{-3}
275 parameters	$\Delta\rangle_{min} = -0.43$ e Å^{-3}

Special details

Geometry. All esds (except the esd in the dihedral angle between two l.s. planes) are estimated using the full covariance matrix. The cell esds are taken into account individually in the estimation of esds in distances, angles and torsion angles; correlations between esds in cell parameters are only used when they are defined by crystal symmetry. An approximate (isotropic) treatment of cell esds is used for estimating esds involving l.s. planes.

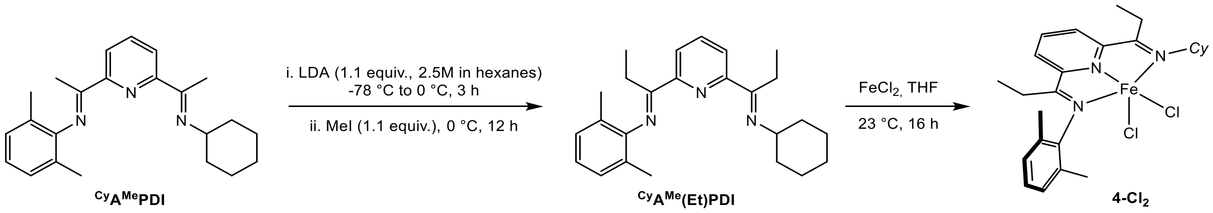

^{Cy}A^{Me}PDI **^{Cy}A^{Me}(Et)PDI** **4-Cl$_2$**

Preparation of C$_S$-symmetric (PDI)FeCl$_2$ (4-Cl$_2$). In a 100ml two-necked round-bottom flask, nBuLi (2.2 equiv., 12.66 mmol, 2.5M in hexanes) was added dropwise at -78 °C to a solution of diisopropylamine (2.2 equiv., 12.66 mmol, 1.78 mL) in dry THF (30 ml) under argon. The solution was stirred for 30min, followed by slow addition of a solution of pyridine(diimine) **^{Cy}A^{Me}PDI** (2.00 g, 5.76 mmol) in dry THF (20 ml). The reaction vessel was warm up to 0 °C and stirred for 3 h. At 0 °C, methyl iodide (12.66 mmol, 0.788 ml) was added dropwise and the reaction was run for 12 h at room temperature. The reaction mixture was then quenched with a saturated solution of $NaHCO_3$ and extracted with ethyl acetate (3 x 100 ml). The organic fractions were combined, dried over $MgSO_4$ and concentrated under vacuum to give the crude desired **^{Cy}A^{Me}(Et)PDI** (2.11 mg, >99% yield)) as a light yellow oil.

The crude material was dried under vacuum overnight and used without further purification in the next step.

1H NMR (500 MHz, chloroform-*d*, 20 ºC): δ 8.40 – 8.23 (m, 1H), 8.17 (d, *J* = 7.8 Hz, 1H), 7.79 (t, *J* = 7.8 Hz, 1H), 7.07 (d, *J* = 7.6 Hz, 2H), 6.93 (dd, *J* = 8.1, 6.8 Hz, 1H), 3.64 (td, *J* = 9.8, 4.8 Hz, 1H), 2.97 (q, *J* = 7.6 Hz, 2H), 2.69 (q, *J* = 7.5 Hz, 2H), 2.06 (s, 6H), 1.78 – 1.23 (m, 10H), 1.17 (t, *J* = 7.6 Hz, 3H), 1.02 (t, *J* = 7.6 Hz, 3H).

^{13}C NMR (126 MHz, chloroform-*d*, 20 ºC): δ 171.81, 168.44, 156.53, 154.16, 148.69, 136.96, 127.99 (2C), 125.54, 122.93, 122.63, 122.08, 59.94, 34.18 (2C), 25.93, 24.91 (2C), 23.42, 20.67, 18.30 (2C), 12.75, 11.41.

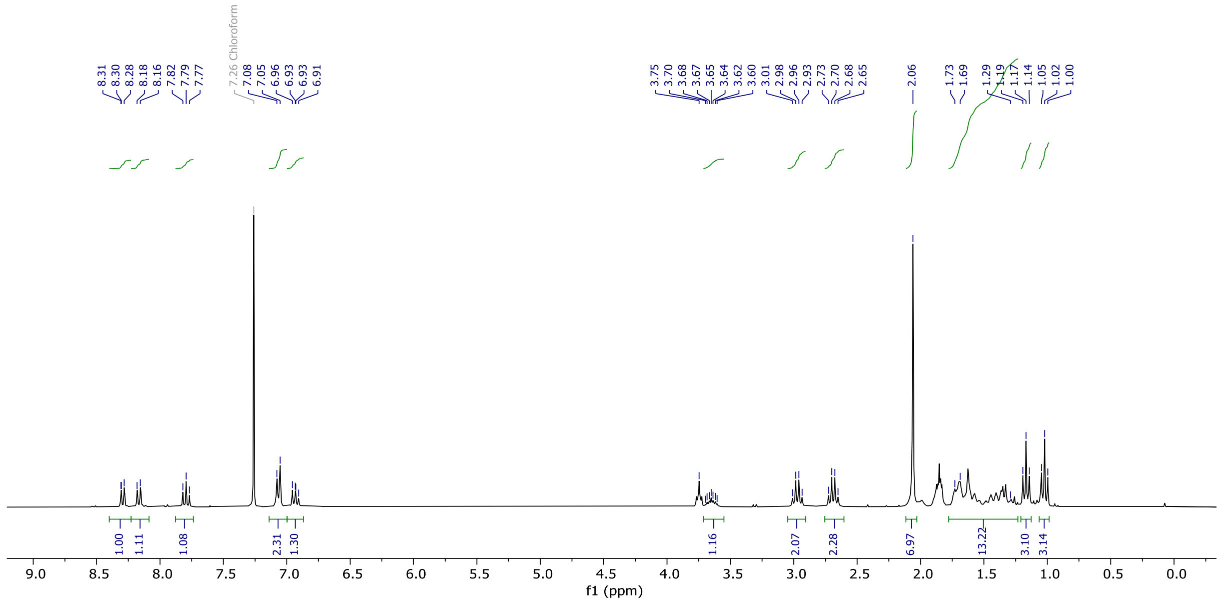

Figure S6. ^{1}H NMR (500 MHz, $CDCl_3$) spectrum of **^{Cy}A^{Me}(Et)PDI**.

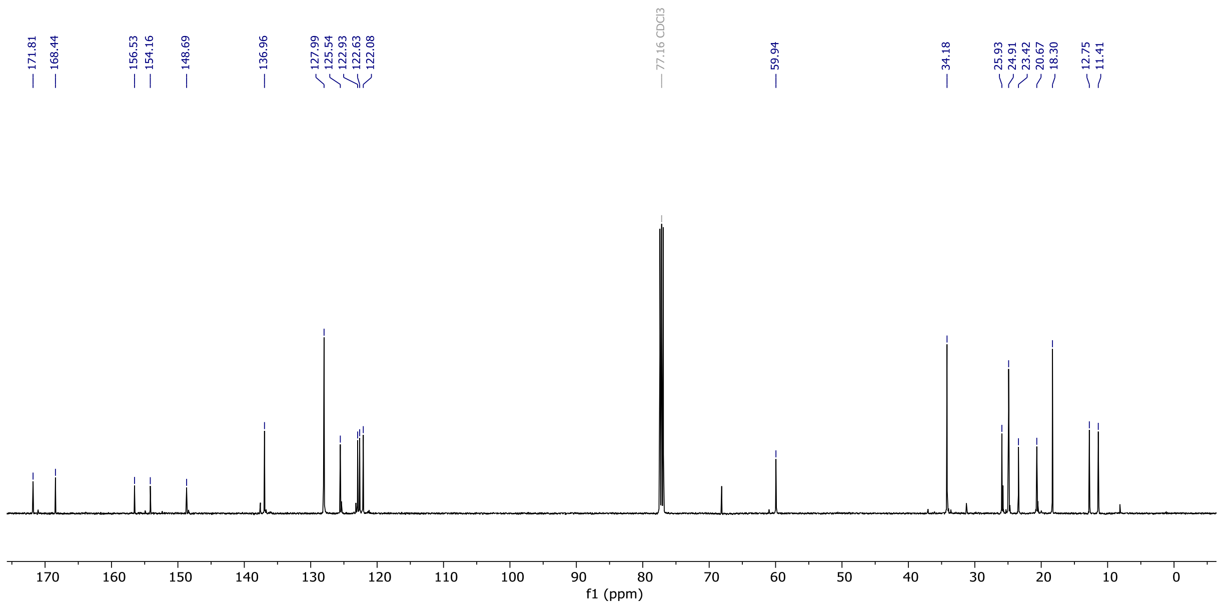

Figure S7. ^{13}C NMR (126 MHz, $CDCl_3$) spectrum of **^{Cy}A^{Me}(Et)PDI**.

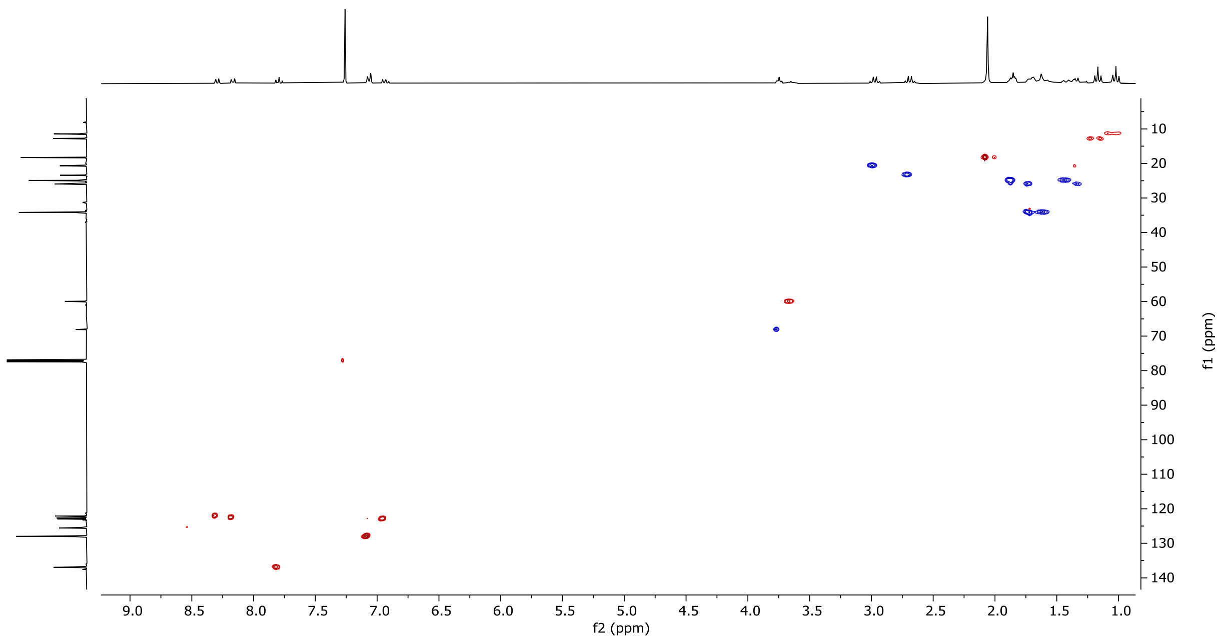

Figure S8. ^{1}H-^{13}C HSQC NMR (500 MHz, 126 MHz, $CDCl_3$) spectrum of **^{Cy}A^{Me}(Et)PDI**.

In a nitrogen-filled glovebox, iron(II) dichloride (695 mg, 5.48 mmol, 0.98 equiv.) was added to a solution of **^{Cy}A^{Me}(Et)PDI** (2.1 g, 5.59 mmol, 1.00 equiv.) in dry THF (20 ml) and the reaction was stirred overnight. The resulting precipitate was then filtered off and rinsed with

a 1:1 mixture of THF and Et_2O and dried over vacuum to give **4-Cl_2** as a blue powder (2.4 g, 4.78 mmol, 85% yield).

1H NMR (400 MHz, dichloromethane-*d_2*, 20 ºC): δ 222.50 (217 Hz), 89.58 (97 Hz), 69.56 (62 Hz), 29.52 (46 Hz), 27.76 (83 Hz), 21.11 (41 Hz), 11.29 (112 Hz), 10.53 (44 Hz), -1.64 (43 Hz), -3.96 (34 Hz), -4.32 (35 Hz), -5.88 (47 Hz), -6.87 (82 Hz), -7.29 (52 Hz), -9.02 (39 Hz), -9.51 (41 Hz), -24.50 (136 Hz), -33.35 (335 Hz).

Analysis for $C_{24}H_{31}Cl_2FeN_3$: Calc. C, 59.78; H, 6.62; N, 8.37. Found: C, 59.76 ; H, 6.57; N, 8.01.

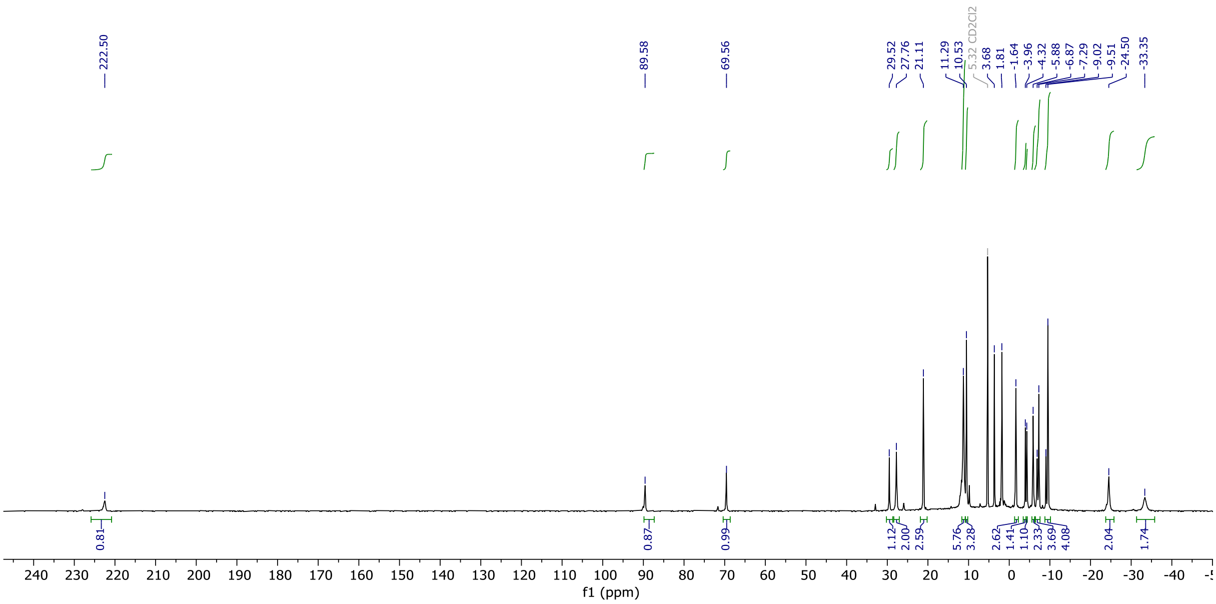

Figure S9. ^{1}H NMR (400 MHz, CD_2Cl_2) spectrum of **4-Cl_2**.

ii. C_S-symmetric (PDI)$FeCH_3$ Complexes.

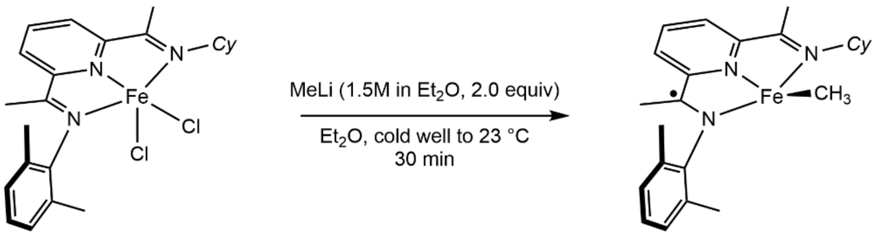

Preparation of 1-CH_3. In a glove-box, a 20 mL scintillation vial was charged with 0.215 g (0.453 mmol) of ($^{(Cy)Me}$PDI)$FeCl_2$ and approximately 15 mL of diethyl ether. The slurry was frozen in a cold well and 0.907 mmol of MeLi (1.5M in diethyl ether, 2.0 equiv.) was added. The solution was stirred as it was warmed to room temperature. After stirring for 30 min, the solution was filtered through Celite and washed with diethyl ether, and the volatiles removed in vacuo. The crude product was recrystallized from diethyl ether at -35 °C overnight to afford a pure sample of **1-CH_3** (0.125 g, 0.299 mmol, 66%) as a dark green solid. Recrystallization from a saturated solution of **1-Cl_2** in diethyl ether over six days at room temperature afforded suitable crystals for single-crystal X-ray diffraction analysis. The structure and coordinates were deposited with the Cambridge Crystallographic Data Center (CCDC#2256985).

1H NMR (400 MHz, cyclohexane-d_{12}, 20 ºC): δ 316.37 (1H, 220 Hz, *p-py*), 80.39 (1H, 105 Hz), 49.76 (1H, 88 Hz), 22.61 (1H, 105 Hz), 2.58 (1H, 24 Hz), -4.54 (2H, 31 Hz, *Cy*), -5.78 (2H, 91 Hz, *Cy*), -13.66 (2H,151 Hz, *Cy*), -15.59 (1H, 22 Hz), -40.20 (1H, 144 Hz), -52.01 (6H, 170 Hz, CH_3-*Aryl*), -68.16 (2H, 225 Hz, *Cy*), -122.29 (3H, 121 Hz, CH_3(C=N)), -214.77 (3H, 179 Hz, CH_3(C=N)). 2H from Cy and Fe-CH_3 *not detected*.

Analysis for $C_{24}H_{32}FeN_3$: Calc. C, 68.90; H, 7.71; N, 10.04. Found: C, 68.56 ; H, 7.60; N, 9.95.

Magnetic Susceptibility Balance (Evans method, 23 °C): μ_{eff} = 4.0 μ_B.

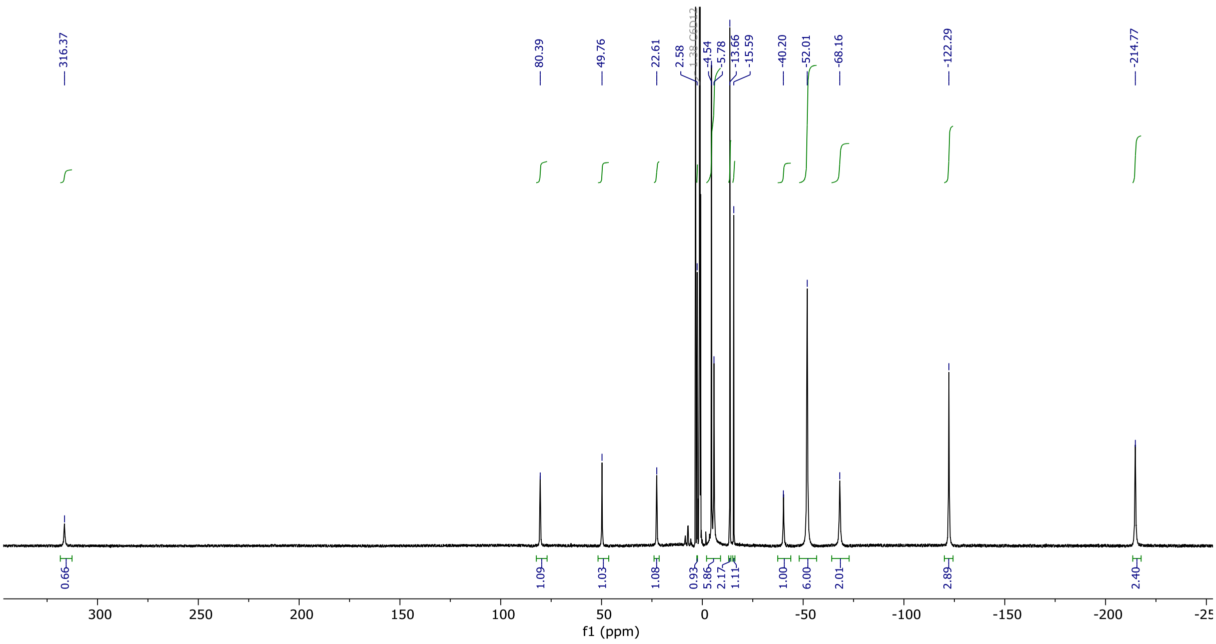

Figure S10. ¹H NMR (400 MHz, C_6D_{12}) spectrum of **1-CH$_3$**.

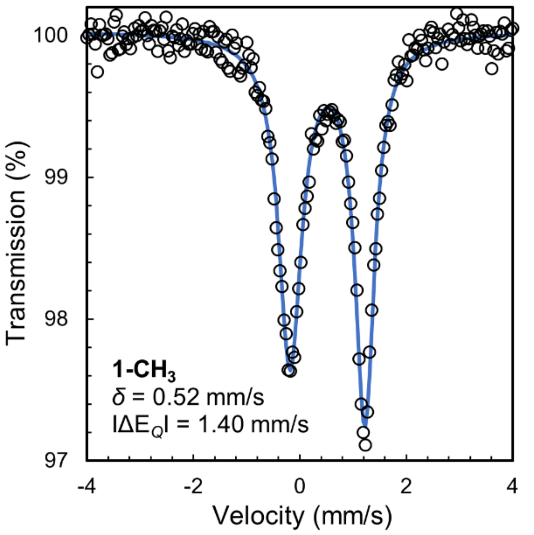

Figure S11. Zero-field 57Fe Mössbauer (solid state, 80 K) spectrum of **1-CH$_3$**.

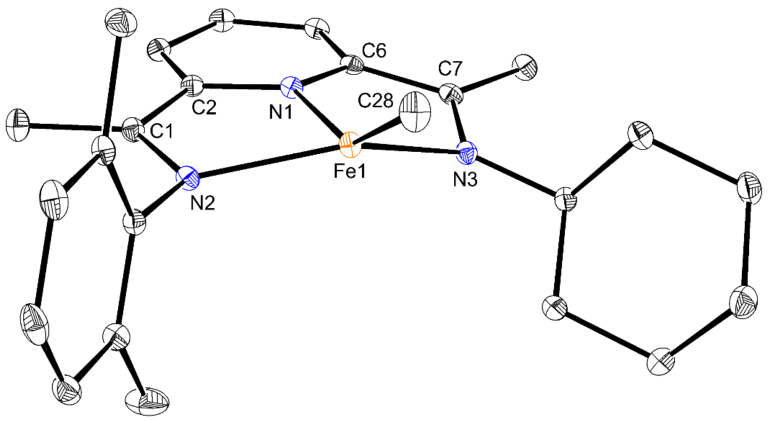

Figure S12. solid-state structure of **1-CH$_3$**, depicted with **30**% probability ellipsoids (CCDC #2256985). Hydrogen atoms are omitted for clarity. C = gray, N = blue, Fe = red-orange.

Table S3: Experimental crystal data for **1-CH$_3$**.

Crystal data	
Chemical formula	$C_{96}H_{128}Br_0Fe_4N_{12}$
M_r	1673.50
Crystal system, space group	Monoclinic, $P2_1/n$
Temperature (K)	100
a, *b*, *c* (Å)	16.792 (6), 7.911 (3), 19.420 (7)
β (°)	110.86 (2)
V (Å^3)	2410.7 (15)
Z	1
Radiation type	Mo $K\alpha$
μ (mm^{-1})	0.64
Crystal size (mm)	0.25 × 0.13 × 0.12
Data collection	
Diffractometer	Bruker *APEX*-II CCD
Absorption correction	Multi-scan<br>TWINABS BRUKER AXS
T_{min}, T_{max}	0.699, 0.746
No. of measured, independent and observed [$I > 2\sigma(I)$] reflections	106091, 6256, 5413
R_{int}	0.059
(sin θ/λ)$_{max}$ (Å^{-1})	0.678
Refinement	

$R[F^2 > 2\sigma(F^2)]$, $wR(F^2)$, S	0.036, 0.100, 1.05
No. of reflections	6256
No. of parameters	258
H-atom treatment	H-atom parameters constrained
$\Delta\rangle_{max}$, $\Delta\rangle_{min}$ (e $Å^{-3}$)	0.51, -0.42

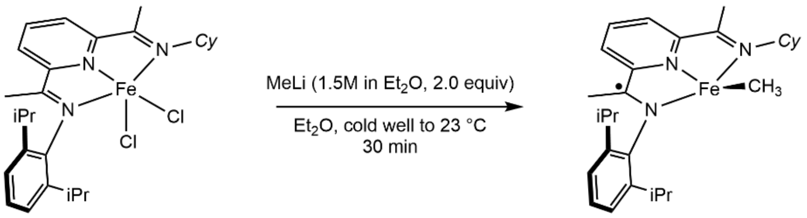

Preparation of 2-CH_3. In a glove-box, a 20 mL scintillation vial was charged with 0.239 g (0.450 mmol) of ($^{(Cy)Me}$PDI)$FeCl_2$ and approximately 15 mL of diethyl ether. The slurry was frozen in a cold well and 0.900 mmol of MeLi (1.5M in diethyl ether, 2.0 equiv.) was added. The solution was stirred as it was warmed to room temperature. After stirring for 30 min, the solution was filtered through Celite and washed with diethyl ether, and the volatiles removed in vacuo. The crude product was recrystallized from pentane at -35 °C overnight to afford a pure sample of **2-CH_3** (0.120 g, 0.253 mmol, 56%) as a dark green solid. Recrystallization from a saturated solution of **2-Cl_2** in pentane over three days at room temperature afforded suitable crystals for single-crystal X-ray diffraction analysis. The structure and coordinates were deposited with the Cambridge Crystallographic Data Center (CCDC#2256984).

1H NMR (400 MHz, cyclohexane-d_{12}, 20 ºC): δ 331.39 (1H, 227 Hz, *p-pyr*), 77.50 (1H, 112 Hz), 52.16 (1H, 96 Hz), 25.67 (1H, 111 Hz), 3.91 (1H, 33 Hz), -4.45 (2H, 86 Hz, *Cy*), -5.73 (2H, 41 Hz, *Cy*), -14.01 (2H, 33 Hz, *Cy*), -17.10 (1H, 30 Hz), -18.01 (6H, 42 Hz, CH(CH_3)-*aryl*), -28.28 (1H, 130 Hz) , -38.41 (6H, 152 Hz, CH(CH_3)-*aryl*), -66.68 (2H, 217 Hz, *Cy*), -95.06 (2H, 533 Hz, C*H*(CH3)-*aryl*), -129.18 (3H, 134 Hz, CH_3(C=N)), -221.48 (3H, 178 Hz, CH_3(C=N)). 2H from Cy and Fe-CH_3 *not detected*.

Analysis for $C_{28}H_{40}FeN_3$: Calc. C, 70.88; H, 8.50; N, 8.86. Found: C, 70.77; H, 8.28; N, 8.53.

Magnetic Susceptibility Balance (Evans method, 23 °C): μ_{eff} = 4.0(3) μ_B.

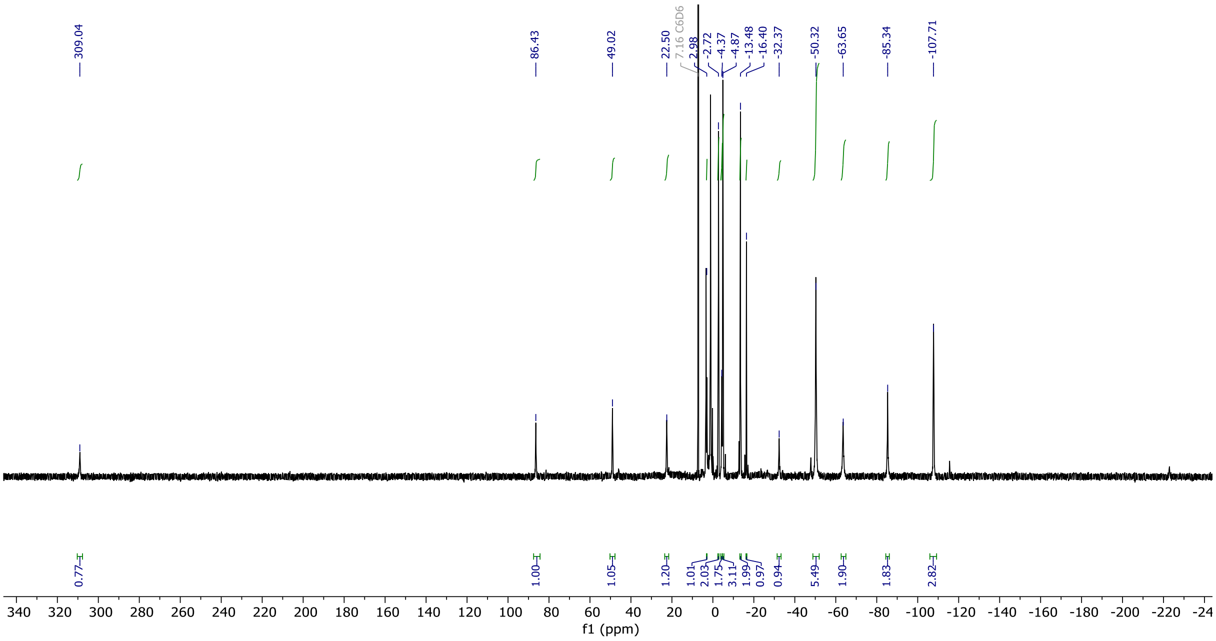

Figure S13. ^{1}H NMR (400 MHz, C_6D_{12}) spectrum of **2-CH$_3$**.

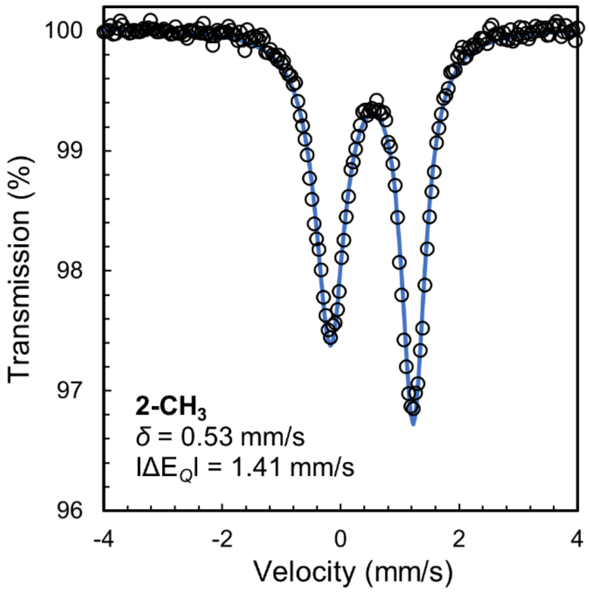

Figure S14. Zero-field 57Fe Mössbauer (solid state, 80 K) spectrum of **2-CH$_3$.**

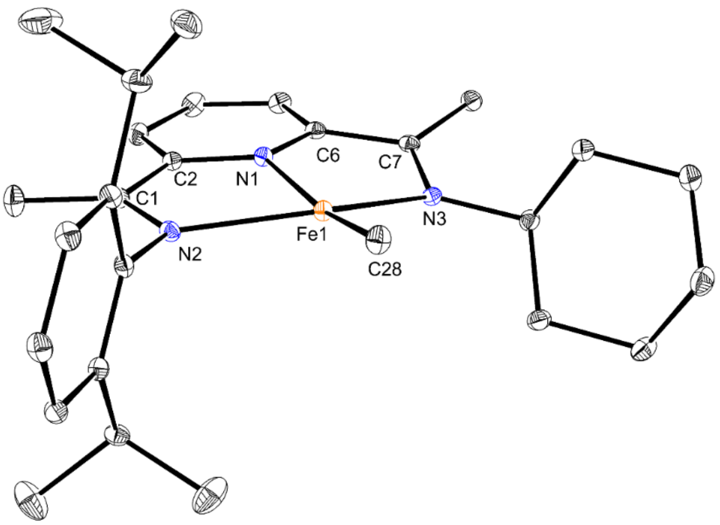

Figure S15. solid-state structure of **2-CH$_3$**, depicted with **30**% probability ellipsoids (CCDC #2256984). Hydrogen atoms are omitted for clarity. C = gray, N = blue, Fe = red-orange.

Table S4: Experimental crystal data for **2-CH$_3$**.

Crystal data	
Chemical formula	$C_{56}H_{80}Fe_2N_6$
M_r	948.96
Crystal system, space group	Monoclinic, $P2_1/n$
Temperature (K)	100
a, *b*, *c* (Å)	8.3837 (2), 20.1567 (4), 15.3689 (3)
β (°)	90.799 (1)
V ($Å^3$)	2596.90 (10)
Z	2
Radiation type	Mo *K*α
μ (mm^{-1})	0.60
Crystal size (mm)	0.16 × 0.11 × 0.10
Data collection	
Diffractometer	Bruker *APEX*-II CCD
Absorption correction	Multi-scan<br>TWINABS BRUKER AXS
T_{min}, T_{max}	0.708, 0.746
No. of measured, independent and observed [$I > 2\sigma(I)$] reflections	52331, 6727, 5338
R_{int}	0.143
$(\sin \theta/\lambda)_{max}$ ($Å^{-1}$)	0.676
Refinement	

$R[F^2 > 2\sigma(F^2)]$, $wR(F^2)$, S	0.039, 0.094, 1.02
No. of reflections	6727
No. of parameters	296
H-atom treatment	H-atom parameters constrained
$\Delta\rangle_{max}$, $\Delta\rangle_{min}$ (e Å^{-3})	0.46, -0.43

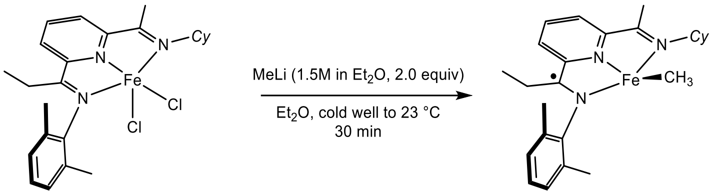

Preparation of 3-CH_3. In a glove-box, a 20 mL scintillation vial was charged with 0.151 g (0.310 mmol) of ($^{(Cy)Me}$PDI)$FeCl_2$ and approximately 15 mL of diethyl ether. The slurry was frozen in a cold well and 0.620 mmol of MeLi (1.6M in diethyl ether, 2.0 equiv.) was added. The solution was stirred as it was warmed to room temperature. After stirring for 30 min, the solution was filtered through Celite and washed with diethyl ether, and the volatiles removed in vacuo. The crude product **3-CH_3** (0.134 g, 0.310 mmol, >99%) was obtained as a dark green solid. No recrystallization was obtained in different combination of solvents at 0 °C or -35 °C.

1H NMR (400 MHz, benzene-*d*$_6$, 20 ºC): δ 309.04 (1H, 224 Hz, *p-pyr*), 86.43 (1H, 113 Hz), 49.02 (1H, 95 Hz), 22.50 (1H, 125 Hz), 2.98 (1H, 34 Hz), -2.72 (2H, 37 Hz, Cy), -4.37 (2H, 136 Hz, Cy), -4.87 (3H, 59 Hz, C*H*$_3$CH$_2$(C=N-Ar)), -13.48 (2H, 34 Hz, Cy), -16.40 (1H, 28 Hz), -32.37 (1H, 173 Hz), -50.32 (6H, 184 Hz, C*H*$_3$-*Aryl*), -63.65 (2H, 259 Hz, Cy), -85.34 (2H, 143 Hz, CH$_3$C*H*$_2$(C=N-Ar)), -107.71 (3H, 116 Hz, C*H*$_3$(C=N-Cy)). 2H from Cy and Fe-CH_3 *not detected*.

Analysis for $C_{25}H_{34}FeN_3$: Calc. C, 69.44; H, 7.93; N, 9.72. Found: C, 69.36; H, 7.67; N, 9.67.

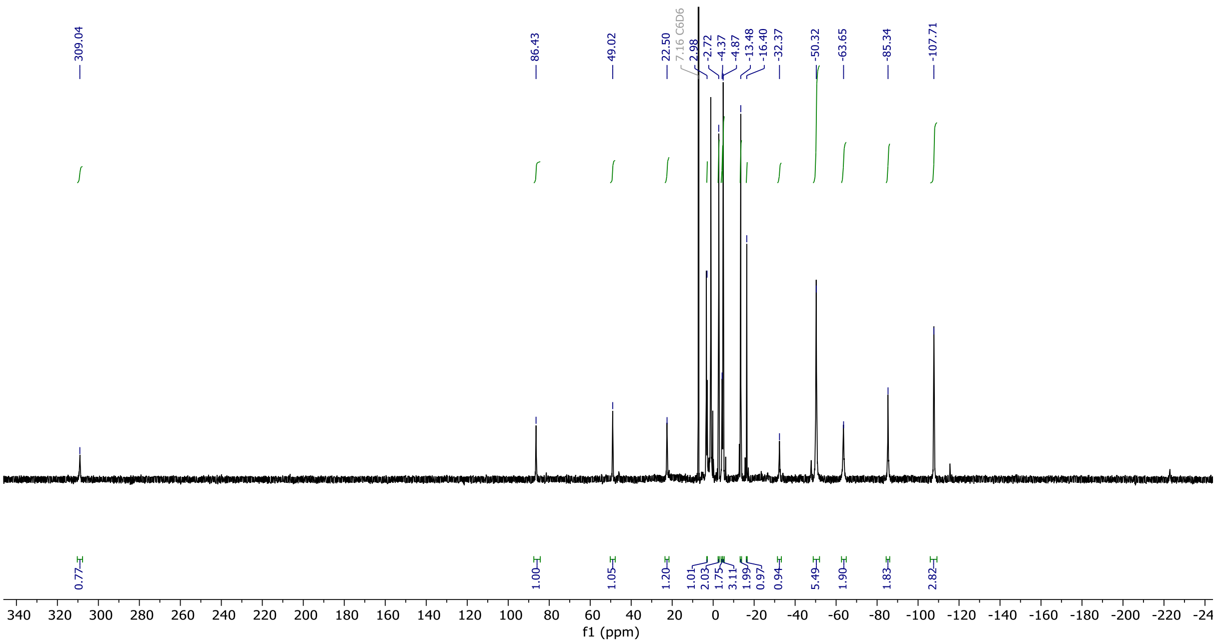

Figure S16. ^{1}H NMR (400 MHz, C_6D_{12}) spectrum of **3-CH₃**.

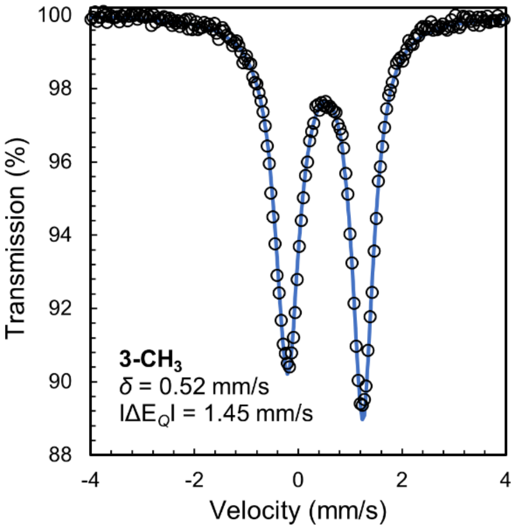

Figure S17. Zero-field 57Fe Mössbauer (solid state, 80 K) spectrum of **3-CH₃**.

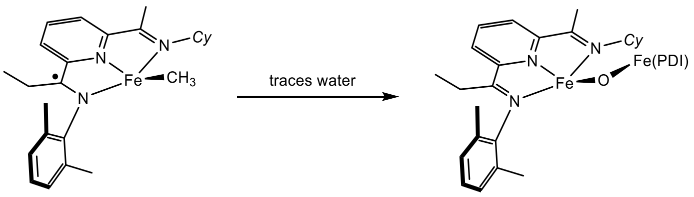

The formation of the side-product $[(PDI)Fe]_2(\mu\text{-}O)$ was observed attributed to accidental traces of water as an oxygen source. The side-product was isolated by extraction from

diethyl ether of a mixture of **3-CH$_3$** and **[3]$_2$(μ-O)** as a green solid. Recrystallization from a saturated solution of **[3]$_2$(μ-O)** in diethyl ether over three days at room temperature afforded suitable crystals for single-crystal X-ray diffraction analysis. The structure and coordinates were deposited with the Cambridge Crystallographic Data Center (CCDC# 2256988). ^{1}H NMR (500 MHz, benzene-*d*$_6$, 20 °C): δ 105.90 (1H, 107 Hz), 104.89 (1H, 100 Hz), 64.71 (1H, 35Hz), 40.84 (1H, 32 Hz), 40.16 (1H, 49 Hz), 33.07 (1H, 30 Hz), 17.16 (1H, 36 Hz), 12.76 (1H, 31 Hz), 10.89 (1H, 40 Hz), 8.62 (1H, 32 Hz), 6.16 (1H, 39 Hz), 5.53 (1H, 46 Hz), 3.55 (3H, 29 Hz), -0.75 (1H, 42 Hz), -3.77 (1H, 39 Hz), -4.19 (1H, 26 Hz), -5.53 (1H, 28 Hz), -10.71 (1H, 28 Hz), -17.88 (3H, 27 Hz) , -26.41 (3H, 34 Hz), -27.77 (1H, 40 Hz), -29.75 (1H, 39 Hz), -83.24 (3H, 44 Hz).

Analysis for $C_{48}H_{62}FeN_6O$: Calc. C, 67.77; H, 7.35; N, 9.88. Found: C, 67.71; H, 7.10; N, 9.86.

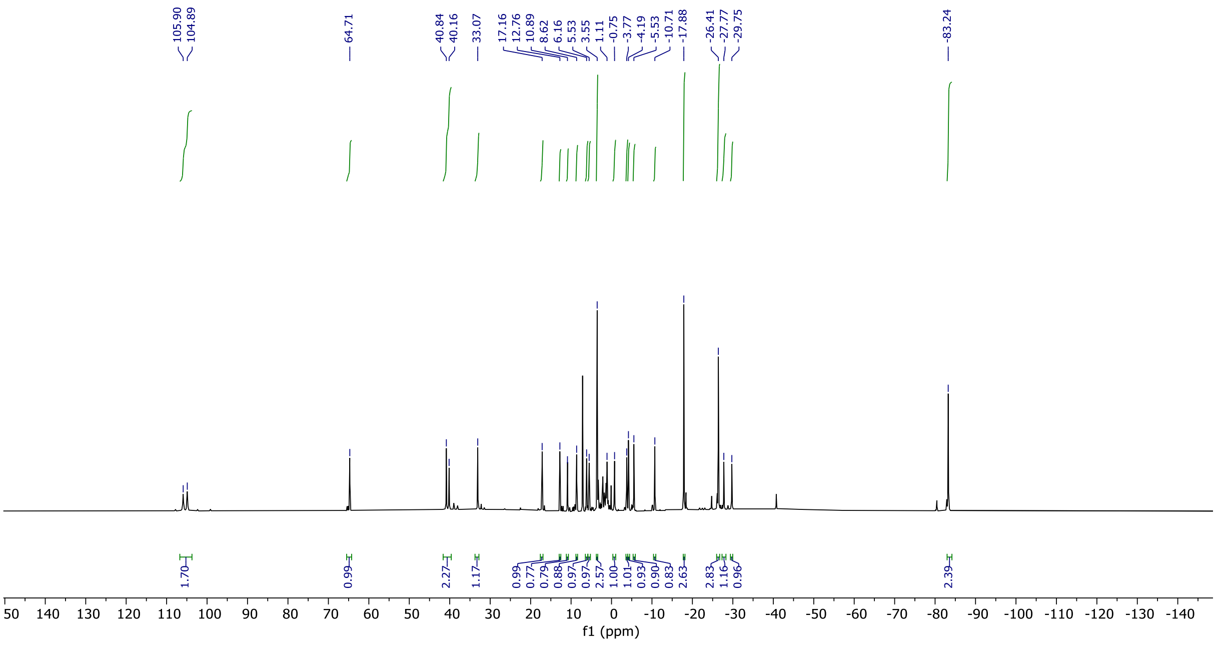

Figure S18. ^{1}H NMR (400 MHz, C_6D_6) spectrum of **[3](μ-O)**.

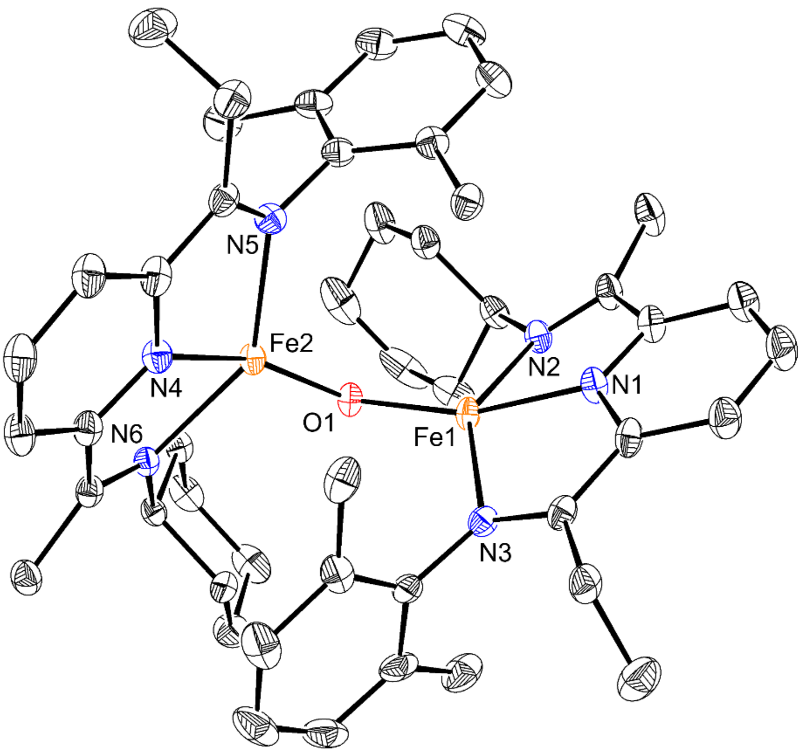

Figure S19. Solid-state structure of **[3]$_2$-(μ-O)** depicted with **30**% probability ellipsoids (CCDC #2256988). Hydrogen atoms are omitted for clarity. C = gray, N = blue, Fe = red-orange, O = red.

Table 5: Experimental crystal data for **[3]$_2$-(μ-O)**.

Crystal data	
Chemical formula	$C_{48}H_{62}Fe_2N_6O$
M_r	850.73
Crystal system, space group	Triclinic, $P\bar{1}$
Temperature (K)	100
a, *b*, *c* (Å)	12.1724 (2), 17.1982 (3), 22.4138 (3)
α, β, γ (°)	81.514 (1), 79.805 (1), 80.149 (1)
V (Å^3)	4516.60 (13)
Z	4
Radiation type	Cu *K*α
μ (mm^{-1})	5.46
Crystal size (mm)	0.21 × 0.12 × 0.10
Data collection	
Diffractometer	Bruker *APEX*-II CCD
Absorption correction	Multi-scan

	TWINABS BRUKER AXS
T_{min}, T_{max}	0.558, 0.753
No. of measured, independent and observed [$I > 2\sigma(I)$] reflections	63211, 15852, 12033
R_{int}	0.066
$(\sin \theta/\lambda)_{max}$ (Å^{-1})	0.596
Refinement	
$R[F^2 > 2\sigma(F^2)]$, $wR(F^2)$, S	0.051, 0.134, 1.01
No. of reflections	15852
No. of parameters	1043
H-atom treatment	H-atom parameters constrained
$\Delta\rangle_{max}$, $\Delta\rangle_{min}$ (e Å^{-3})	0.79, -0.35

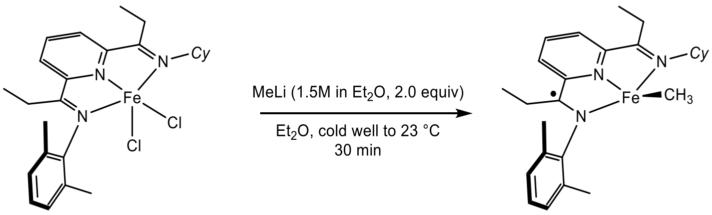

Preparation of 4-CH_3. In a glove-box, a 20 mL scintillation vial was charged with 0.269 g (0.535 mmol) of **4-Cl_2** and approximately 15 mL of diethyl ether. The slurry was frozen in a cold well and 1.070 mmol of MeLi (1.6M in diethyl ether, 2.0 equiv.) was added. The solution was stirred as it was warmed to room temperature. After stirring for 30 min, the solution was filtered through Celite and washed with diethyl ether, and the volatiles removed in vacuo. The crude product **4-CH_3** (0.232 g, 0.520 mmol, 97%) was obtained as a dark green solid. No preparative recrystallization was obtained in different combination of solvents at 0 °C or -35 °C. 1H NMR (400 MHz, benzene-d_6, 20 ºC): δ 311.62 (1H, 217 Hz, *p-pyr*), 86.88 (1H, 116 Hz), 48.58 (1H, 91 Hz), 23.03 (1H, 116 Hz), 2.96 (1H, 30 Hz), -2.22 (2H, 33 Hz), -5.40 (5H, 52 Hz, Cy and CH_3CH_2(C=N-Ar)), -12.73 (2H, 46 Hz, CH_3CH_2(C=N-Ar)), -13.23 (2H, 31 Hz, Cy), -15.54 (1H, 23 Hz), -18.49 (1H, 46 Hz), -32.05 (2H, 101 Hz, CH_3CH_2(C=N-Ar)), -51.06 (6H, 182 Hz, CH_3-*Aryl*), -62.60 (2H, 231 Hz, Cy), -83.50 (2H, 138 Hz, CH_3CH_2(C=N-Ar)). 2H from Cy and Fe-CH_3 *not detected*.

Analysis for $C_{26}H_{36}FeN_3$: Calc. C, 69.95; H, 8.13; N, 9.41. Found: C, 69.27; H, 7.18; N, 8.75.

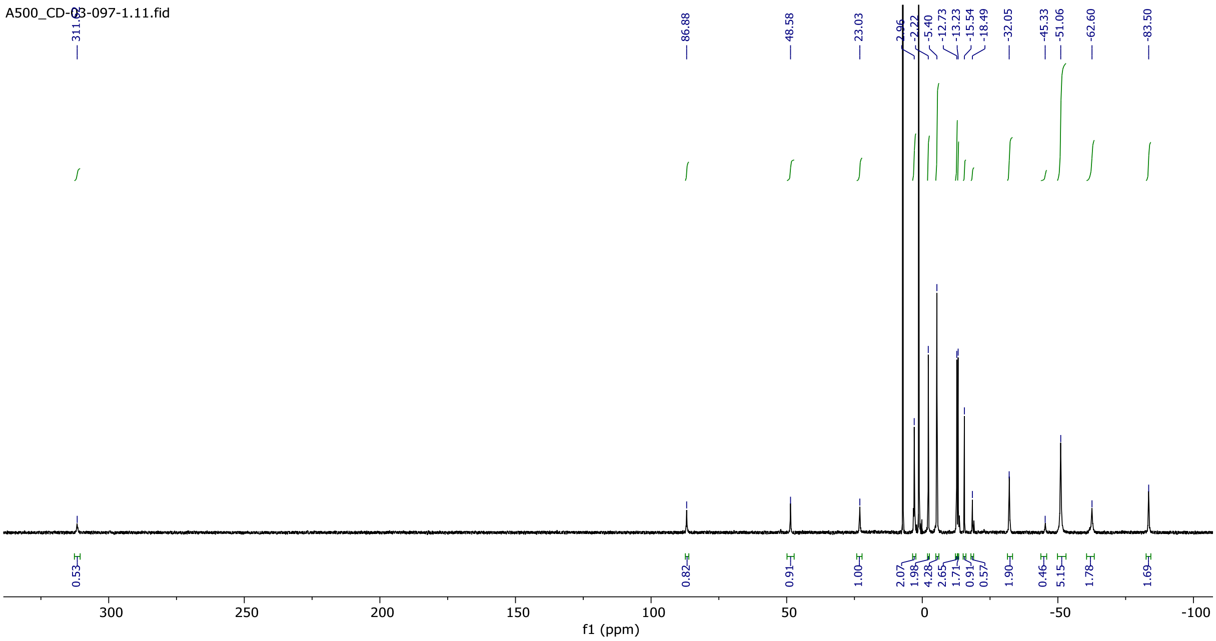

Figure S20. ^1H NMR (500 MHz, C_6D_6) spectrum of **4-CH$_3$**.

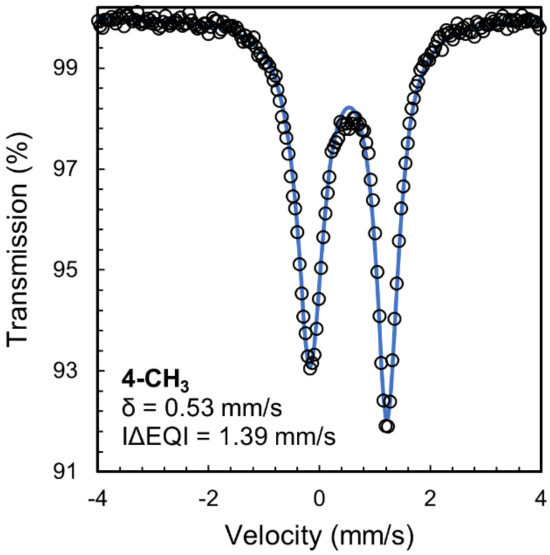

Figure S21. Zero-field 57Fe Mössbauer (solid state, 80 K) spectrum of **4-CH$_3$**.

iii. Dimeric Dinuclear Bridging Methyl Complex 5.

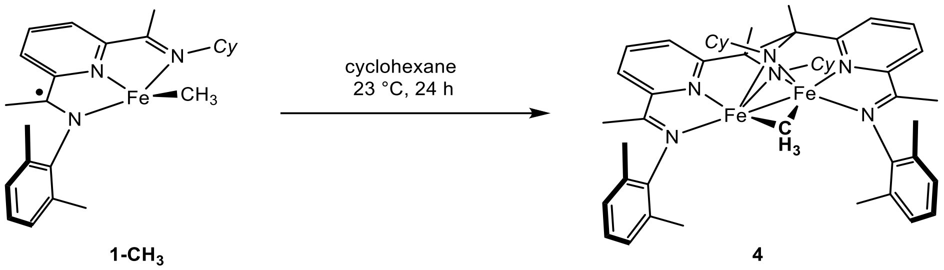

Preparation of 5. In a glove-box, a 20 mL scintillation vial was charged with 0.126 g (0.300 mmol) of **1-CH_3** and approximately 6 mL of cyclohexane. The solution was kept at room temperature without stirring for 24 h. The solution was then filtered through Celite and washed with cyclohexane, and the volatiles removed in vacuo. The crude product was recrystallized from diethyl ether at -35 °C overnight to afford an analytically pure sample of **5** (0.064 g, 0.253 mmol, 52%) as a orange/brown solid. Crystals suitable for single-crystal X-ray diffraction analysis were obtained following a second recrystallization in diethyl ether at -35 °C for 1 week. The structure and coordinates were deposited with the Cambridge Crystallographic Data Center (CCDC #2256986). The presence of a bridging methyl fragment, as opposite to methylene fragment, was confirmed by methanolysis, as solely CH_4 and CH_3D were detected by ^1H NMR spectroscopy after adding CD_3OD to a C_6D_6 solution of **5**.

^{1}H NMR (400 MHz, cyclohexane-*d_{12}*, 20 ºC): δ 82.51 (1H, 358 Hz), 77.30 (1H, 675 Hz), 65.10 (1H, 100 Hz), 42.83 (1H, 308 Hz), 8.46 (3H, 64 Hz), 5.72 (3H, 87 Hz), 3.03 (1H, 17 Hz), 2.80 (1H, 27 Hz), 1.74 (1H, 19 Hz), 0.68 (1H, 28 Hz), -1.73 (2H, 26 Hz), -3.58 (1H, 35 Hz), -4.93 (1H, 35 Hz), -5.00 (1H, 36 Hz), -11.35 (1H 242 Hz), -13.26 (3H, 76 Hz), -14.42 (1H, 360 Hz), -15.24 (1H, 225 Hz), -27.03 (1H, 616 Hz), -88.04 (1H, 1765 Hz), 2H and Fe-CH_3 *not detected*.

Analysis for $C_{47}H_{61}FeN_6$: Calc. C, 68.70; H, 7.48; N, 10.23. Found: C, 68.52; H, 7.13; N, 9.84.

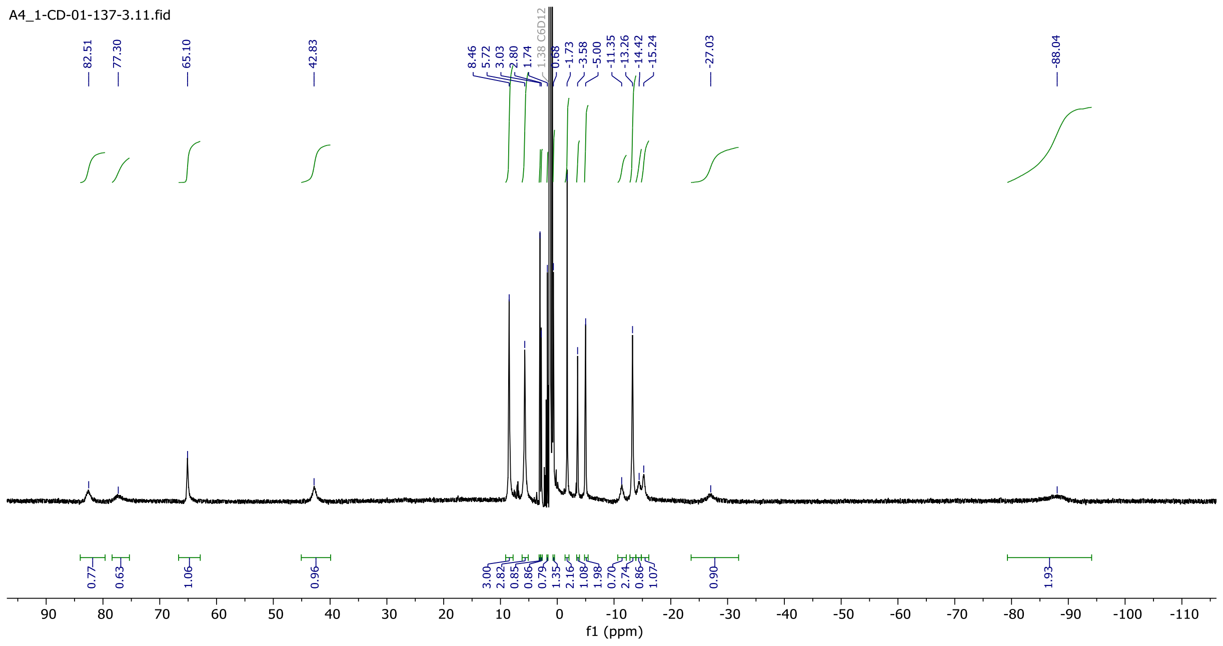

Figure S22. ^{1}H NMR (400 MHz, C_6D_{12}) spectrum of **5**.

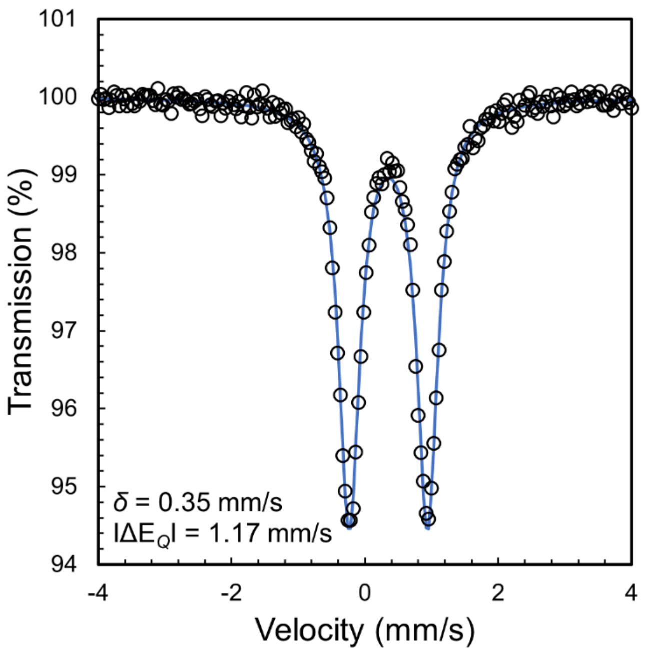

Figure S23. Zero-field ^{57}Fe Mössbauer (solid state, 80 K) spectrum of **5**

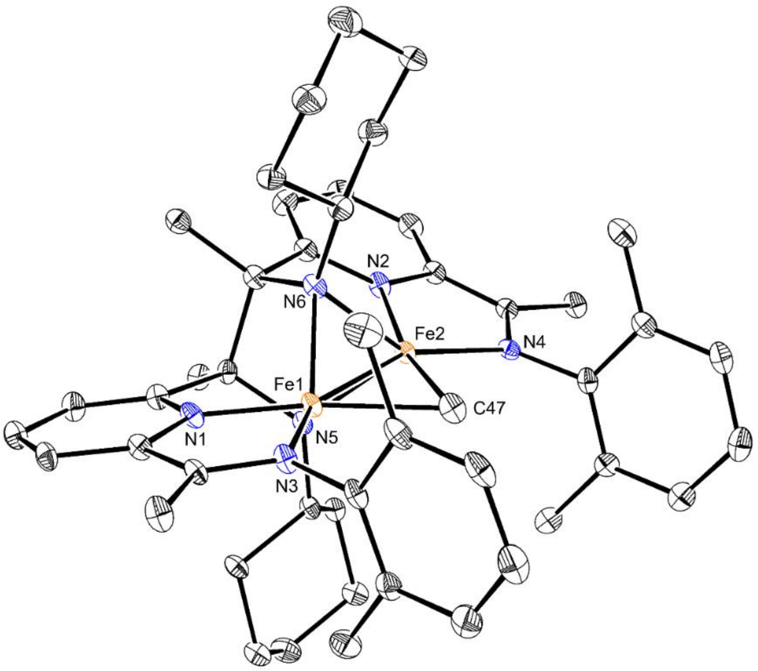

Figure S24. Solid-state structure of **5** depicted with 30% probability ellipsoids (CCDC #2256986). Hydrogen atoms are omitted for clarity. C = gray, N = blue, Fe = red-orange.

Table S6: Experimental crystal data for **5**.

Crystal data

$C_{47}H_{60}Fe_2N_6$	Z = 2
M_r = 820.71	F(000) = 872
Triclinic, $P\bar{1}$	D_x = 1.321 Mg m^{-3}
a = 11.041 (6) Å	Cu $K\alpha$ radiation, λ = 1.54178 Å
b = 11.225 (6) Å	Cell parameters from 9960 reflections
c = 16.84 (2) Å	θ = 4.0–66.6°
α = 96.69 (6)°	μ = 5.94 mm^{-1}
β = 94.65 (7)°	T = 100 K
γ = 92.42 (5)°	Plate, black
V = 2064 (3) Å^3	0.13 × 0.12 × 0.06 mm

Data collection

Bruker APEX-II CCD diffractometer	4950 reflections with $I > 2\sigma(I)$
ϕ and ω scans	R_{int} = 0.113
Absorption correction: multi-scan TWINABS BRUKER AXS	θ_{max} = 67.1°, θ_{min} = 2.7°

T_{min} = 0.589, T_{max} = 0.753	h = -13→13
31960 measured reflections	k = -13→13
7246 independent reflections	l = -20→20

Refinement

Refinement on F^2	0 restraints
Least-squares matrix: full	Hydrogen site location: inferred from neighbouring sites
$R[F^2 > 2\sigma(F^2)]$ = 0.056	H-atom parameters constrained
$wR(F^2)$ = 0.132	$w = 1/[\sigma^2(F_o^2) + (0.0574P)^2]$ where $P = (F_o^2 + 2F_c^2)/3$
S = 1.02	$(\Delta/\sigma)_{max}$ < 0.001
7246 reflections	$\Delta\rangle_{max}$ = 0.61 e Å^{-3}
498 parameters	$\Delta\rangle_{min}$ = -0.50 e Å^{-3}

Special details

Geometry. All esds (except the esd in the dihedral angle between two l.s. planes) are estimated using the full covariance matrix. The cell esds are taken into account individually in the estimation of esds in distances, angles and torsion angles; correlations between esds in cell parameters are only used when they are defined by crystal symmetry. An approximate (isotropic) treatment of cell esds is used for estimating esds involving l.s. planes.

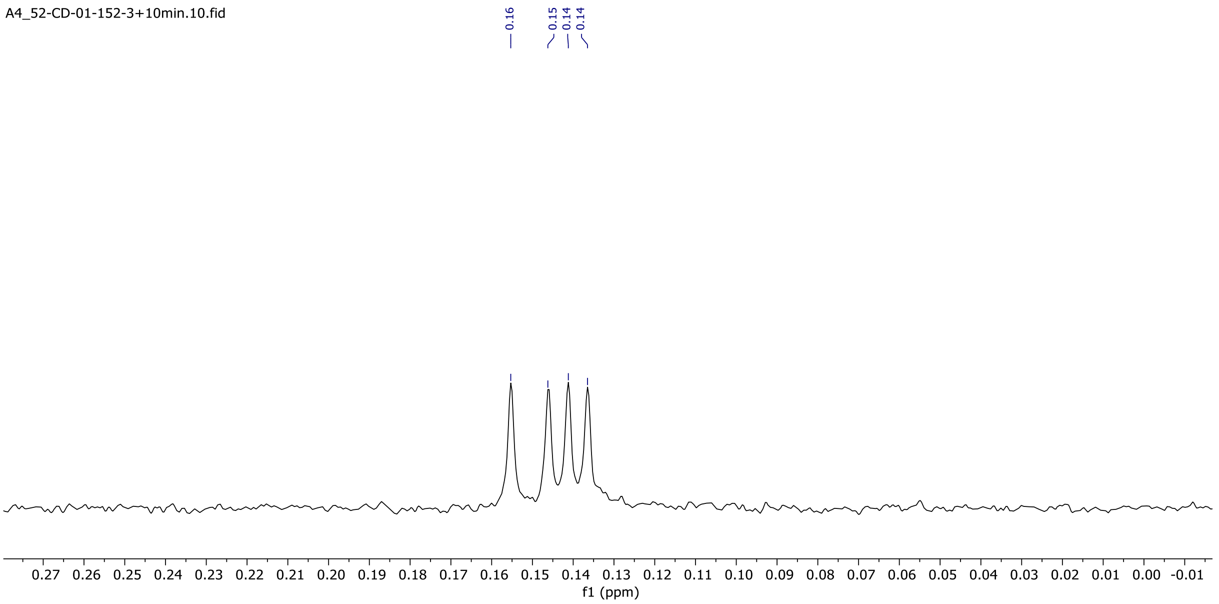

Figure S25. Truncated ^{1}H NMR (400 MHz, C_6D_6) spectrum of the reaction of **5** with CD_3OD.

iii. C_S-symmetric (PDI)Fe(CO)$_2$ Complex.

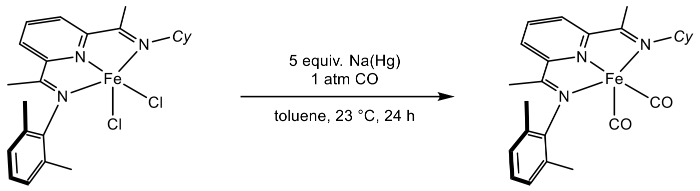

1-Cl$_2$ 1-(CO)$_2$

Preparation of 1-(CO)$_2$: Following the reported procedure,[vi] a thick-wall vessel was charged with mercury (6.47 g, 32.24 mmol) and toluene (5 mL), then Sodium (32.51 mg, 1.41 mmol) was added portion wise under vigorous stirring. The resulting amalgam was stirred for 10 minutes to ensure complete dissolution. A suspension of **1-Cl$_2$** (134.13 mg, 282.83 μmol) in toluene (5 ml) was added to the reaction vessel which was then sealed. The resulting mixture was brought to -196 °C, and the vessel was evacuated. One atmosphere of CO was added and the reaction mixture was stirred for 24 h. The resulting green mixture was decanted away from the amalgam and filtered through a pad of Celite. The solvent was removed in vacuo and the residue was recrystallized from pentane/toluene at -35 °C to give a olive green solid identified as **1-(CO)$_2$** (82 mg, 178.1 μmol, 63% yield). Crystals were suitable for single-crystal X-ray diffraction analysis and the structure and coordinates were deposited with the Cambridge Crystallographic Data Center (CCDC# 2256983).

^{1}H NMR (400 MHz, benzene-*d*$_6$, 20 ºC): δ 7.77 – 7.64 (m, 2H), 7.19 (t, *J* = 7.7 Hz, 1H), 7.09 – 6.99 (m, 3H), 4.00 (s, 1H), 2.36 – 2.22 (m, 2H), 2.19 (s, 3H), 2.00 (s, 6H), 1.96 (s, 3H), 1.96 – 1.87 (m, 2H), 1.77 – 1.66 (m, 2H), 1.55 – 1.47 (m, 1H), 1.28 – 1.16 (s, 3H). **^{13}C NMR (126 MHz, benzene-*d*$_6$, 20 ºC):** δ 217.29 (2C, CO), 154.82 (1C, CH$_3$(*C*=N)), 154.40 (1C, CH$_3$(*C*=N)), 153.04 (1C, ipso-aryl), 146.07 (1C, ipso-pyr), 144.50 (1C, ipso-pyr), 129.90 (2C, o-aryl), 128.59 (2C, m-aryl), 125.82 (1C, p-aryl), 120.62 (1C, m-pyr), 120.21 (1C, m-pyr), 116.09 (1C, p-pyr), 36.60 (2C, Cy), 26.60 (2C, Cy), 25.85 (2C, Cy), 18.45 (2C, *C*H$_3$-aryl), 15.11 (2C, *C*H$_3$(C=N)). **IR (pentane):** ν_{CO} 1964, 1904 cm^{-1}. **Analysis for $C_{25}H_{29}FeN_3O_2$**: Calc. C, 65.37; H, 6.36; N, 9.15. Found: C, 64.95; H, 6.30; N, 8.90.

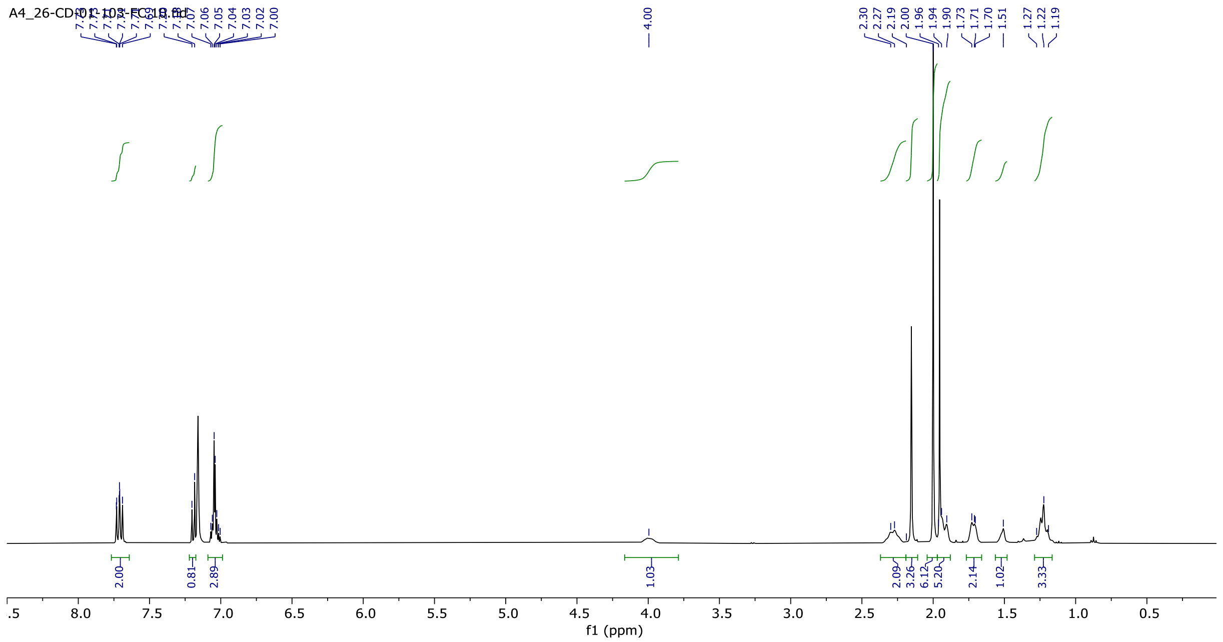

Figure S26. ^{1}H NMR (400 MHz, C_6D_6) spectrum of **1-(CO)₂**.

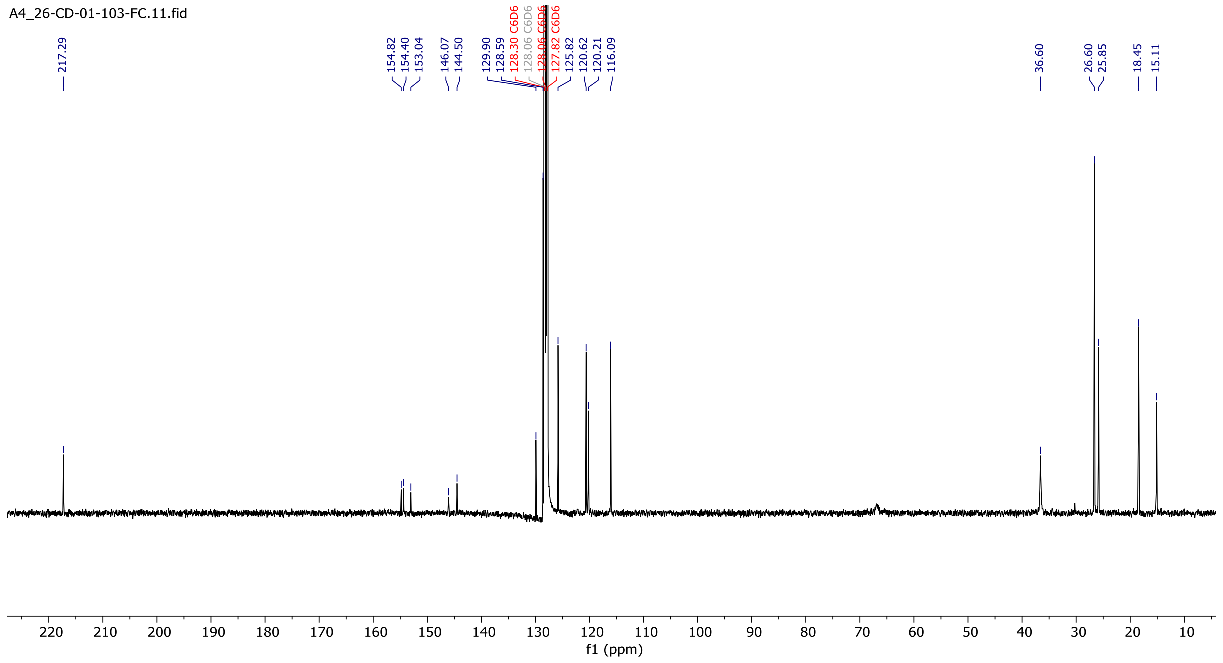

Figure S27. ^{13}C NMR (126 MHz, C_6D_6) spectrum of **1-(CO)₂**.

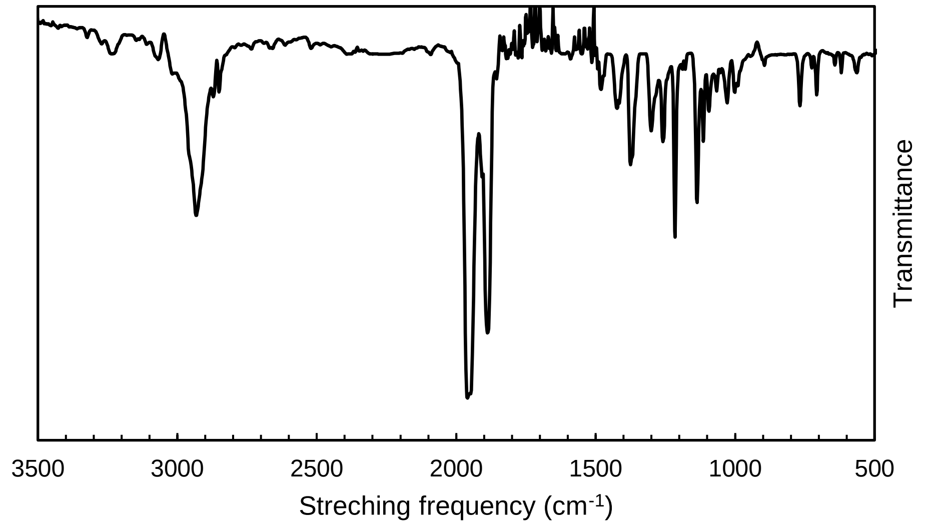

Figure S28. IR spectrum (pentane, 23 °C) of **1-(CO)₂**.

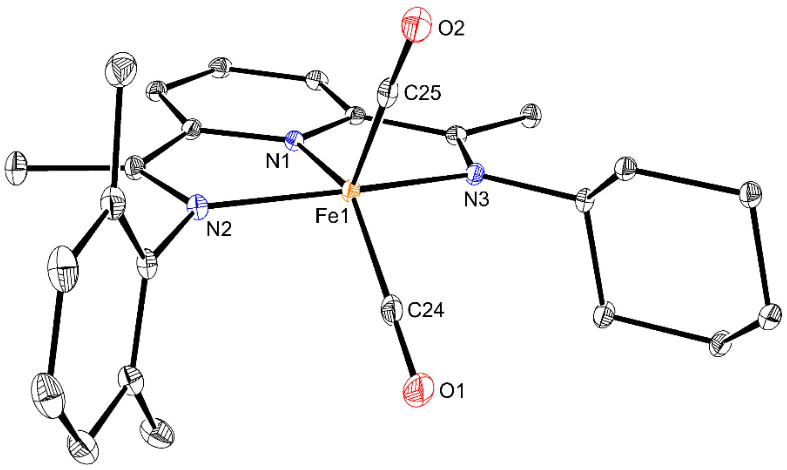

Figure S29. Solid-state structure of **1-(CO)₂** depicted with **30**% probability ellipsoids (CCDC #2256983). Hydrogen atoms are omitted for clarity. C = gray, N = blue, Fe = red-orange, O = red.

Table S7: Experimental crystal data for **1-(CO)₂**.

Crystal data

$C_{25}H_{29}Br_0FeN_3O_2$	$Z = 2$
$M_r = 459.36$	$F(000) = 484$
Triclinic, $P\bar{1}$	$D_x = 1.231$ Mg m^{-3}
$a = 8.717$ (3) Å	Mo $K\alpha$ radiation, $\lambda = 0.71073$ Å

b = 9.145 (4) Å	Cell parameters from 9996 reflections
c = 16.280 (7) Å	θ = 2.4–28.7°
α = 74.470 (16)°	μ = 0.63 mm^{-1}
β = 82.24 (2)°	T = 100 K
γ = 88.067 (13)°	Plate, brown
V = 1239.0 (9) Å^3	0.20 × 0.13 × 0.06 mm

Data collection

Bruker APEX-II CCD diffractometer	5520 reflections with $I > 2\sigma(I)$
ϕ and ω scans	R_{int} = 0.060
Absorption correction: multi-scan TWINABS BRUKER AXS	θ_{max} = 28.7°, θ_{min} = 2.3°
T_{min} = 0.716, T_{max} = 0.746	h = -11→11
66137 measured reflections	k = -12→11
6406 independent reflections	l = -21→21

Refinement

Refinement on F^2	0 restraints
Least-squares matrix: full	Hydrogen site location: inferred from neighbouring sites
$R[F^2 > 2\sigma(F^2)]$ = 0.031	H-atom parameters constrained
$wR(F^2)$ = 0.076	$w = 1/[\sigma^2(F_o^2) + (0.0273P)^2 + 0.8614P]$ where $P = (F_o^2 + 2F_c^2)/3$
S = 1.01	$(\Delta/\sigma)_{max}$ = 0.001
6406 reflections	$\Delta\rangle_{max}$ = 0.43 e Å^{-3}
284 parameters	$\Delta\rangle_{min}$ = -0.33 e Å^{-3}

Special details

Geometry. All esds (except the esd in the dihedral angle between two l.s. planes) are estimated using the full covariance matrix. The cell esds are taken into account individually in the estimation of esds in distances, angles and torsion angles; correlations between esds in cell parameters are only used when they are defined by crystal symmetry. An approximate (isotropic) treatment of cell esds is used for estimating esds involving l.s. planes.

iv. (PDI)$_2$Fe complex 6.

Preparation of (^{Cy}A^{Me}PDI)$_2$Fe (6). Following the reported procedure,[vii] In a nitrogen-filled glovebox, a round-bottom flask was loaded with 1-Cl$_2$ (200 mg, 0.422 mmol, 1 equiv.), Na^0 (20 mg, 0.886 mmol, 2.1 equiv.), naphthalene (2.7 mg, 0.021 mmol, 0.05 equiv.) and THF

(10 ml). The reaction was run for 20 h during which time the color solution changed from blue to green to orange. The mixture was concentrated and then the residue was dissolved into diethyl ether, transferred to a 20 ml vial and topped with pentane. After 20 h at -35 °C, crystals of **6** (38 mg, 0.051 mmol, 12% yield) were obtained, separated from the mother liquor and dried under vacuum. Crystals suitable for single-crystal X-ray diffraction analysis were obtained following a second recrystallization in pentane at -35 °C for 1 week. The structure and coordinates were deposited with the Cambridge Crystallographic Data Center (CCDC #2256987). **^{1}H NMR** (500 MHz, benzene-d_6, 20 ºC): δ 196.03 (876 Hz), 74.12 (340 Hz), 72.45 (311 Hz), 10.61 (575 Hz), 8.31 (205 Hz), 5.55 (45 Hz), 3.52 (66 Hz), 2.24 (178 Hz), 0.62 (42 Hz), -0.32 (99 Hz), -1.36 (124 Hz), -4.55 (130 Hz), -4.66 (45 Hz), -151.21 (1023 Hz), -161.19 (1303 Hz).

Synthesis of (^{Cy}A^{Me}PDI)$_2$Fe from the methyl bridging dimer 6: In a nitrogen-filled glovebox, to a J. Young tube was added **5** (10 mg, 0.012 mmol) in benzene-d_6 (0.5 mol). The tube was sealed and heated to 60 °C for 24h after which time full conversion of **5** to (^{Cy}A^{Me}PDI)$_2$Fe was observed by ^{1}H NMR spectrum of the sample.

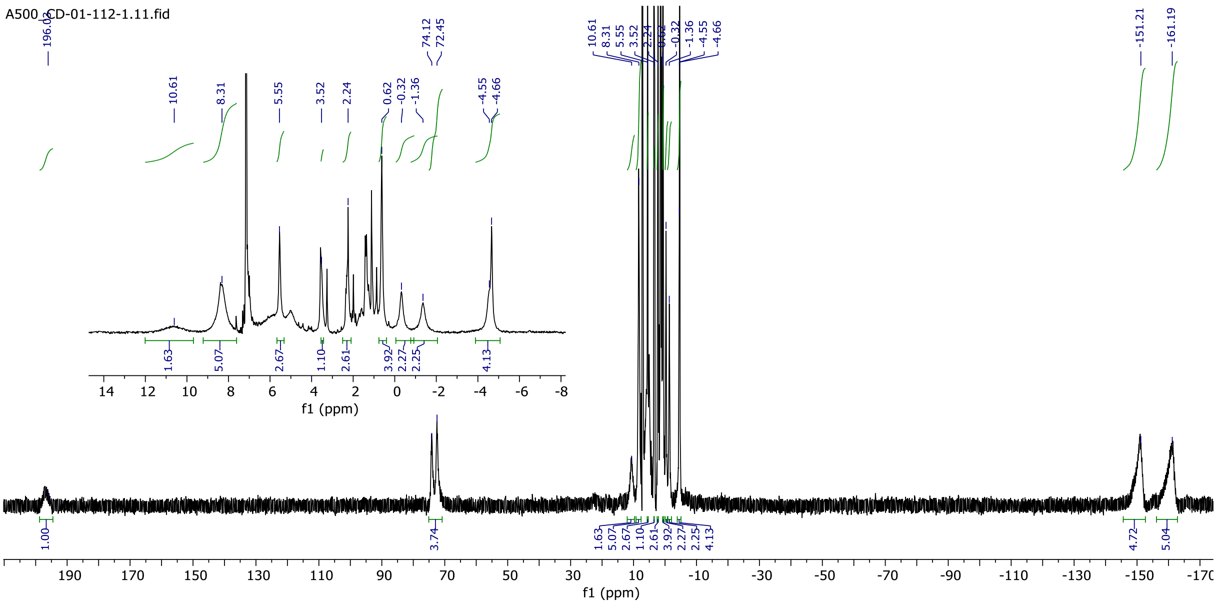

Figure S30. ^{1}H NMR (400 MHz, C_6D_{12}) spectrum of **6**.

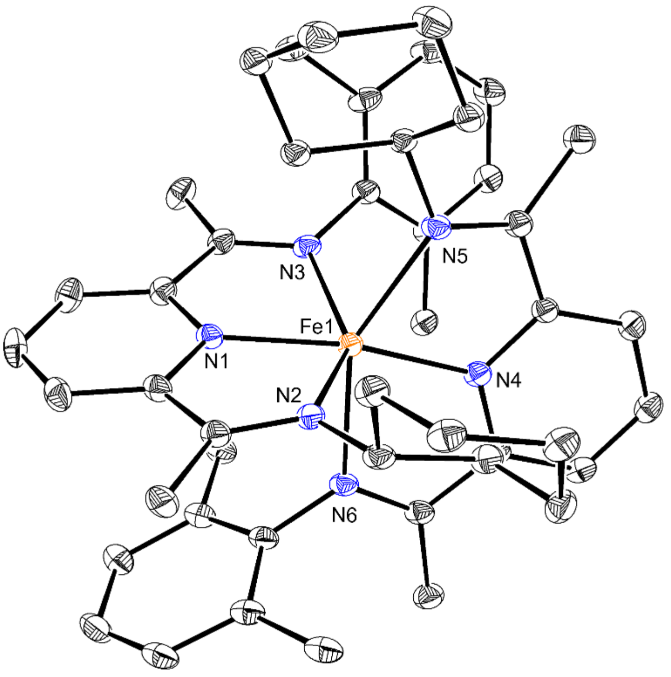

Figure S31. Solid-state structure of **6** depicted with **30**% probability ellipsoids (CCDC #2256987). Hydrogen atoms are omitted for clarity. C = gray, N = blue, Fe = red-orange.

Table S8: Experimental crystal data for **6**.

Crystal data

$C_{46}H_{58}FeN_6$	$Z = 2$
$M_r = 750.83$	$F(000) = 804$
Triclinic, $P\bar{1}$	$D_x = 1.181$ Mg m^{-3}
$a = 10.3124$ (3) Å	Cu $K\alpha$ radiation, $\lambda = 1.54178$ Å
$b = 13.9551$ (4) Å	Cell parameters from 8638 reflections
$c = 15.9871$ (5) Å	$\theta = 2.8$–$66.7°$
$\alpha = 77.932$ (2)°	$\mu = 3.15$ mm^{-1}
$\beta = 80.724$ (2)°	$T = 100$ K
$\gamma = 70.546$ (2)°	Plate, black
$V = 2110.94$ (11) Å^3	0.11 × 0.09 × 0.04 mm

Data collection

Bruker APEX-II CCD diffractometer	5285 reflections with $I > 2\sigma(I)$
ϕ and ω scans	$R_{int} = 0.121$

Absorption correction: multi-scan TWINABS BRUKER AXS	θ_{max} = 66.8°, θ_{min} = 2.8°
T_{min} = 0.556, T_{max} = 0.753	h = -12→12
32263 measured reflections	k = -16→16
7417 independent reflections	l = -19→19

Refinement

Refinement on F^2	0 restraints
Least-squares matrix: full	Hydrogen site location: inferred from neighbouring sites
$R[F^2 > 2\sigma(F^2)]$ = 0.063	H-atom parameters constrained
$wR(F^2)$ = 0.180	$w = 1/[\sigma^2(F_o^2) + (0.1087P)^2]$ where $P = (F_o^2 + 2F_c^2)/3$
S = 0.97	$(\Delta/\sigma)_{max}$ = 0.001
7417 reflections	$\Delta\rangle_{max}$ = 0.95 e Å^{-3}
486 parameters	$\Delta\rangle_{min}$ = -0.71 e Å^{-3}

Special details

Geometry. All esds (except the esd in the dihedral angle between two l.s. planes) are estimated using the full covariance matrix. The cell esds are taken into account individually in the estimation of esds in distances, angles and torsion angles; correlations between esds in cell parameters are only used when they are defined by crystal symmetry. An approximate (isotropic) treatment of cell esds is used for estimating esds involving l.s. planes.

v. Preparation and analytic procedure for 3-(N_2)$_2$.

In a nitrogen-filled glovebox, **3-CH_3** (50 mg, 0.116 mmol) was dissolved into hexanes (2 ml) and transferred to a 20 ml scintillation vial. The solution was maintained at -35 °C for two weeks after which the green solution turned deep red. The cold solution was transferred to a cold IR cell using a chilled glass pipette. The cell was brought out of the glovebox for IR measurement within 5 minutes. The temperature of the sample was estimated to not reached higher than 0 °C over the time of the spectrum recording. A second spectrum was recorded after the sample reached room temperature showing disappearance of the stretching frequency peaks observed at 2091 and 2067 cm^{-1} and color change from deep red to brown.

IR (hexanes, 0 °C) : ν(N_2) 2091, 2067 cm^{-1}.

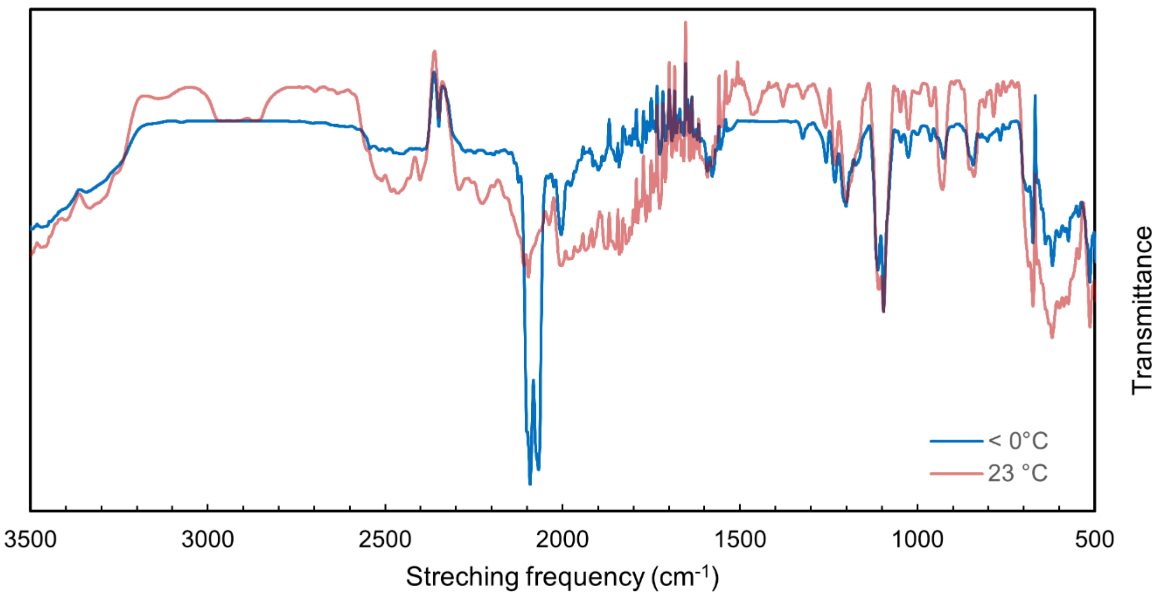

Figure S32. IR spectrum (hexanes, < 0 °C and 23 °C) of **3-$(N_2)_2$**.

In a nitrogen-filled glovebox, **3-CH_3** (50 mg, 0.116 mmol) was dissolved into hexanes (0.5 ml) and transferred to a Mössbauer cell for liquid samples. The solution was maintained at -35 °C for 7 days and after this time the cell was transferred to the Mössbauer instrument for measurement.

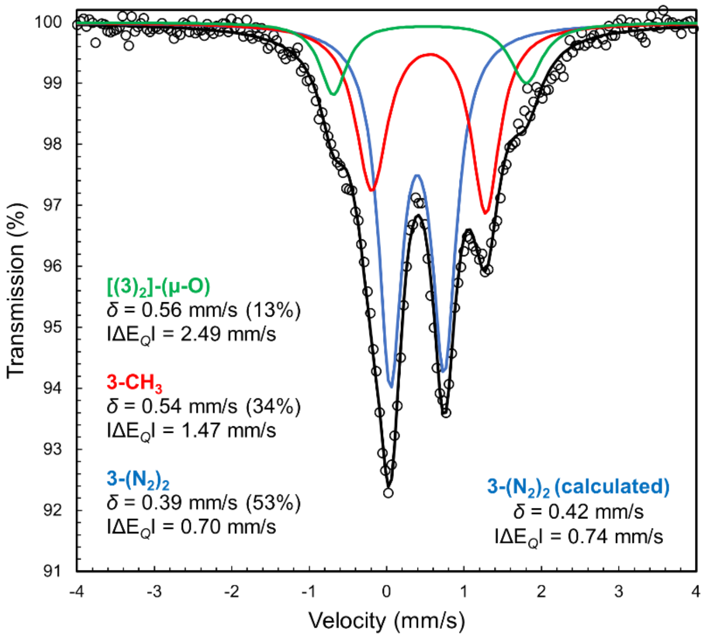

Figure S33. Zero-field ^{57}Fe Mössbauer (solid state, 80 K) spectrum of **3-(N$_2$)$_2$**.

III. Iron-catalyzed [2+2]-cycloaddition/hydrovinylation of ethylene and butadiene

i. Extended Optimization

Table S9: Optimization for the conversion of butadiene and ethylene.

Entry	[Fe]	Modification to conditions	t [h]	C_4H_6 conv. [%]	product ratio		
					VCB	1,4-HD	2,4-HD
1	**1-CH$_3$**	-	14	>99	51	47	2
2	**1-CH$_3$**	-	6	48	51	49	0
3	**1-CH$_3$**	-	12	80	51	49	0
4	**2-CH$_3$**	-	20	>99	11	68	21
5	**3-CH$_3$**	-	6	68	51	49	0
6	**4-CH$_3$**	-	6	60	50	50	0
7	**1-CH$_3$**	2 mol% [Fe]	6	17	50	50	0
8	**1-CH$_3$**	10 mol% [Fe]	6	93	54	45	1
9	**1-CH$_3$**	40 °C	2	70	55	43	2
10	**1-CH$_3$**	40 °C	4	99	55	33	12
11	**4-CH$_3$**	in C_7D_8	4	49	50	50	0
12	**4-CH$_3$**	in C_7D_8, 0 °C	24	30	36	64	0
13	**1-CH$_3$**	+ VCB (0.44 mmol)	17	>99	74	12	14
14	**1-CH$_3$**	+ 4-methylpent-1-ene (0.22 mmol)	17	>99	51	47	2
15	**1-CH$_3$**	+ 1-hexene (0.22 mmol)	15	>99	49	48	3
16	**1-CH$_3$**	1.5 equiv. C_2H_4	15	>99	44	50	6
17	**1-CH$_3$**	1.5 equiv. C_4H_6	25	65%	56	43	1

ii. Time-course of the Reaction

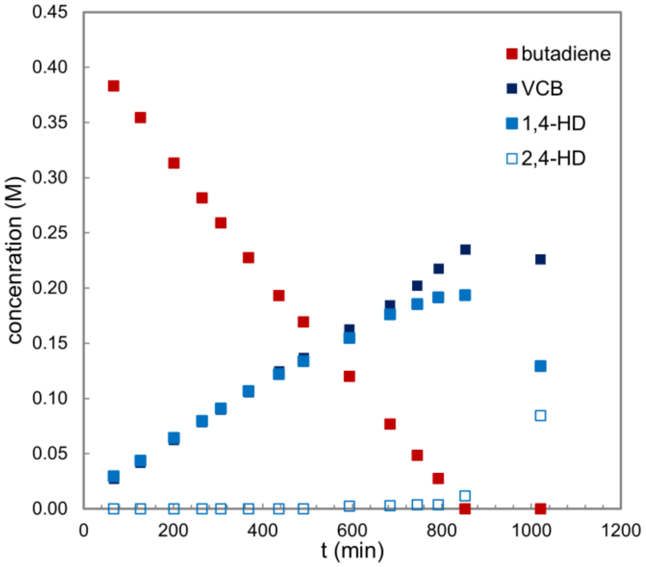

Figure S34. Time course of the cycloaddition/hydrovinylation of butadiene and ethylene with **1-CH_3** (5 mol%) as catalyst.

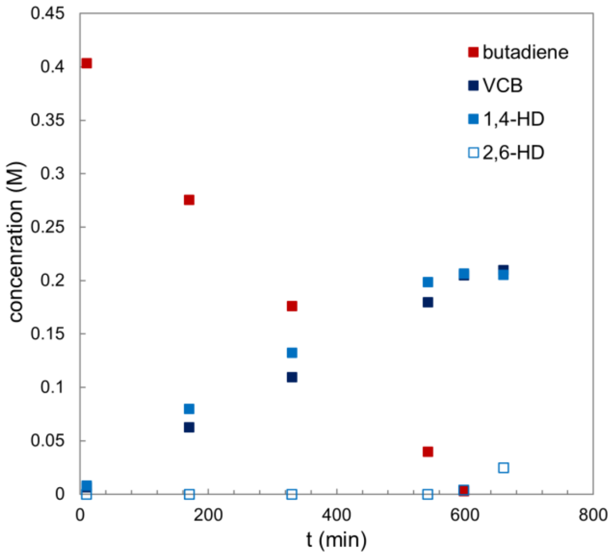

Figure S35. Time course of the cycloaddition/hydrovinylation of butadiene and ethylene with **4-CH_3** (5 mol%) as catalyst.

IV. In-situ Characterization of the Catalyst Resting States.

i. NMR Spectroscopy

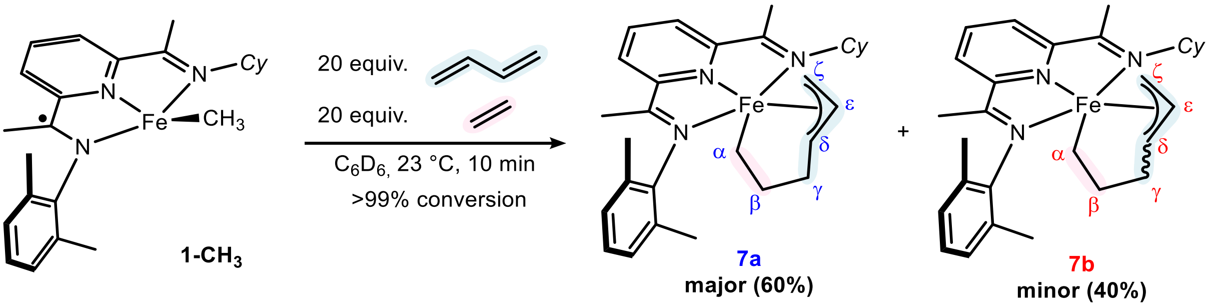

Characterization for the (PDI) ligand fragment: In a nitrogen-filled glovebox, a solution of **1-CH_3** (0.046 mg, 0.11 mmol) in 500mg of benzene-d_6 was transferred to a J. Young tube. The tube was sealed, removed from the glovebox and frozen in liquid dinitrogen. The head-space was evacuated and butadiene (0.44 mmol) followed by ethylene (0.44 mmol) were added by vacuum transfer via a calibrated bulb. The tube was sealed under static vacuum, thawed and mixed by inversion for 10 minutes at room temperature. 1H NMR spectrum was recorded at room temperature and reveals the formation of the title compounds in >99% overall yield and ethane as a byproduct. $^{13}C\{^{1}H\}$ APT, HSQC, NOESY NMR spectra were recorded at room temperature showing parallel conversion of the organic substrates to vinylcyclobutane and (*Z*)-hexa-1,4-diene. The proton and carbon chemical shifts of the (PDI) ligand are summarized in Table S10.

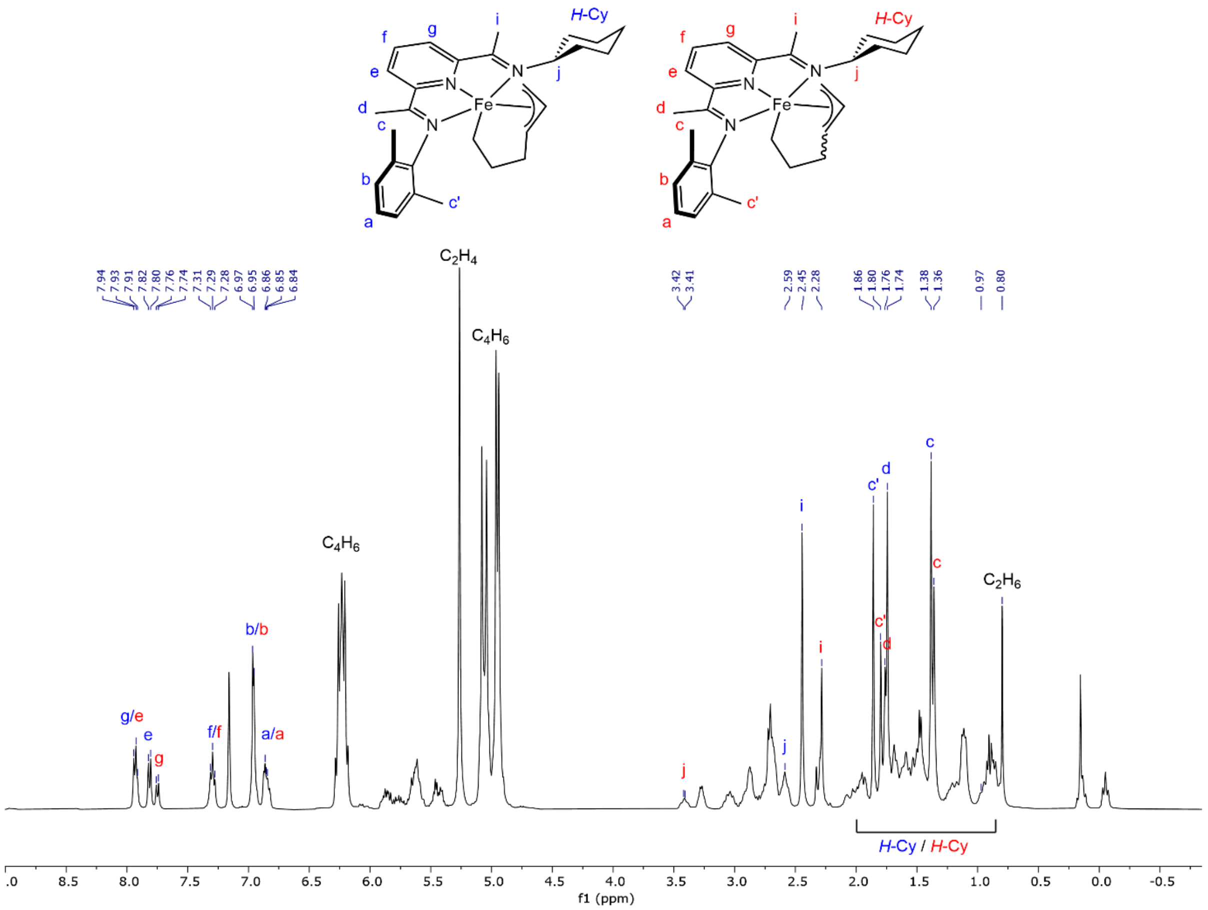

Figure S36. ^{1}H NMR (400 MHz, benzene-d_6) spectrum of **7a** and **7b.**

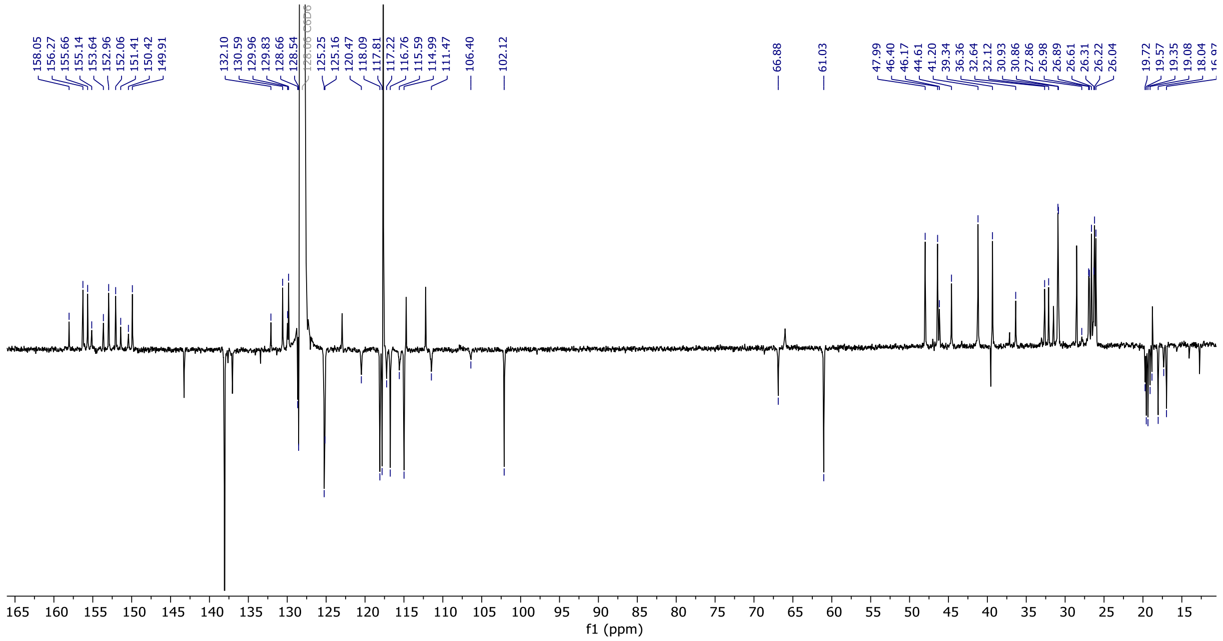

Figure S37. ^{13}C APT NMR (101 MHz, benzene-d_6) spectrum of **7a** and **7b.** (organic fraction chemical shifts are omitted for clarity)

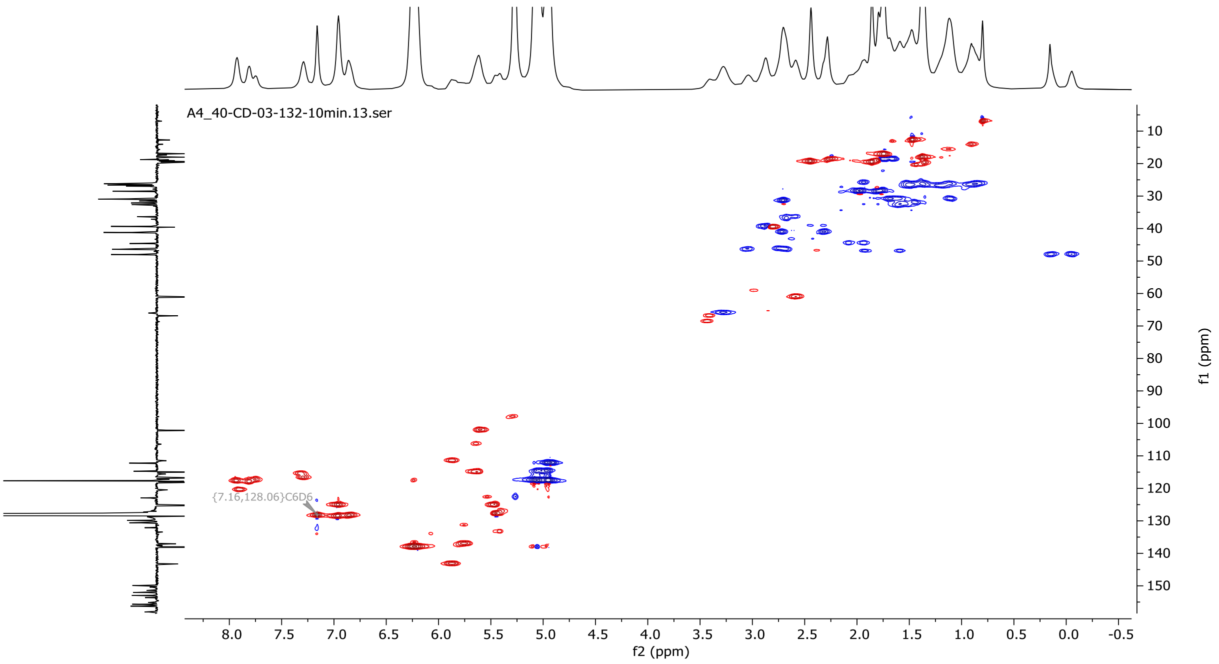

Figure S38. 1H{13C} HSQC (400 MHz, benzene-d_6) spectrum of **7a** and **7b.**

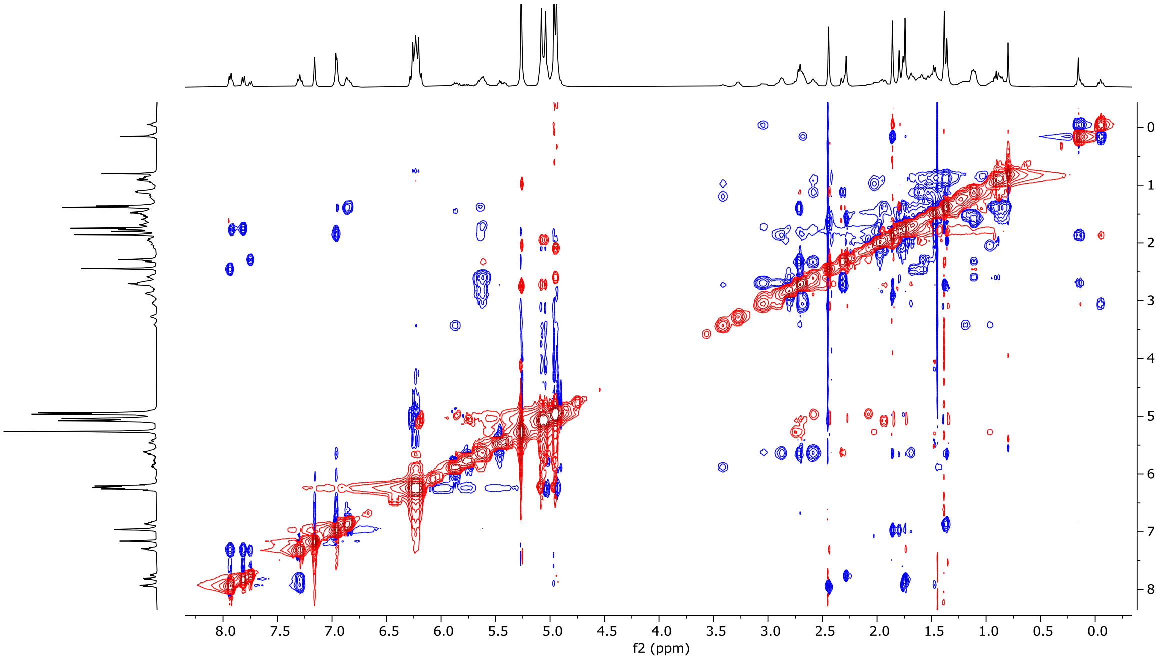

Figure S39. 1H{1H} NOESY (101 MHz, benzene-d_6) spectrum of **7a** and **7b.** (organic fraction chemical shifts are omitted for clarity)

Table 10 : Chemical shifts of **7a** and **7b** (PDI) ligand fragment.

		7a major isomer		7b minor isomer	
**fragment**	**position**	**^{1}H signals (ppm)**	**^{13}C signals (ppm)**	**^{1}H signals (ppm)**	**^{13}C signals (ppm)**

aryl	Ipso	-	151.97	-	151.44
	o (c side)	-	129.84	-	129.99
	o (c' side)	-	130.63	-	132.16
	m (b or b')	6.96	128.53	6.96	128.66
	m (b or b')	6.96	125.19	6.96	125.16
	p (a)	6.85	under benzene	6.85	under benzene
	CH_3 (c)	1.38	18.04	1.36	19.08
	CH_3 (c')	1.86	19.57	1.80	19.72
pyridine	2 (aryl)	-	149.93	-	150.42
	3 (e)	7.81	118.08	7.93	120.48
	4 (f)	7.29	115.59	7.29	116.77
	5 (g)	7.94	117.64	7.75	117.23
	6 (alkyl)	-	153.03	-	155.12
imine	C imine (aryl)	-	155.66	-	153.64
	methyl (aryl) (d)	1.74	16.97	1.76	17.33
	C imine (alkyl)	-	156.12	-	158.02
	methyl (alkyl) (i)	2.44	19.35	2.28	18.79
cyclohexyl	N-C*H* (j)	2.59	61.03	3.42	66.88
	CH_2	0.80 -1.71	30.93	0.80 -1.71	32.64
			30.86		32.12
			26.61		26.98
			26.22		26.89
			26.04		26.31

Characterization of the metallacycle fragment: In a nitrogen-filled glovebox, a solution of **1-CH$_3$** (0.009 mg, 0.022 mmol) in 500mg of benzene-d_6 was transferred to a J. Young tube. The tube was sealed, removed from the glovebox and frozen in liquid dinitrogen. The head-space was evacuated and butadiene (0.44 mmol) followed by ^{13}C-enriched ethylene (0.44 mmol) were added by vacuum transfer via a calibrated bulb. The tube was sealed under static vacuum, thawed and mixed by inversion for 10 minutes at room temperature. 1H NMR spectrum was recorded at room temperature and reveals the formation of the title compounds in >99% overall yield and ethane as a byproduct. 13C{1H} and HSQC NMR spectrum of the was recorded at room temperature showing parallel conversion of the organic substrates to ^{13}C enriched vinylcyclobutane and (*Z*)-hexa-1,4-diene. Ethylene fragment proton and carbon chemical shifts were determined by analysis of the NMR spectra obtained and are reported and Table S11.

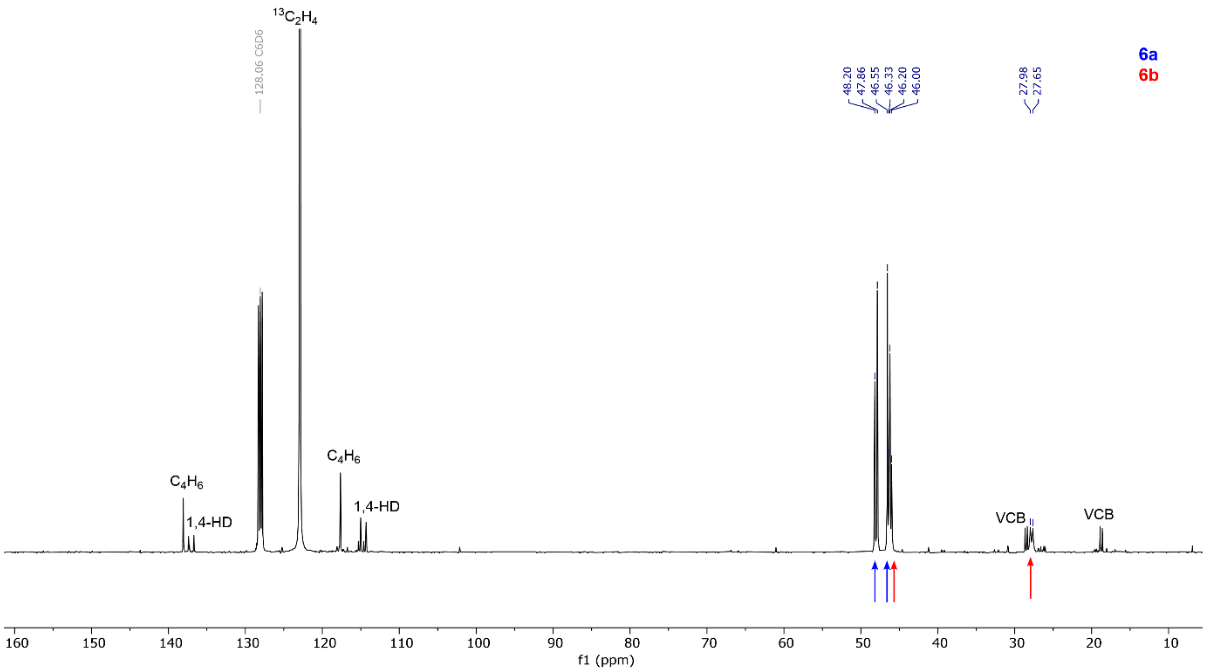

Figure S40. ^{13}C NMR (126 MHz, benzene-d_6) spectrum of **7a** and **7b** generated with 13C enriched ethylene.

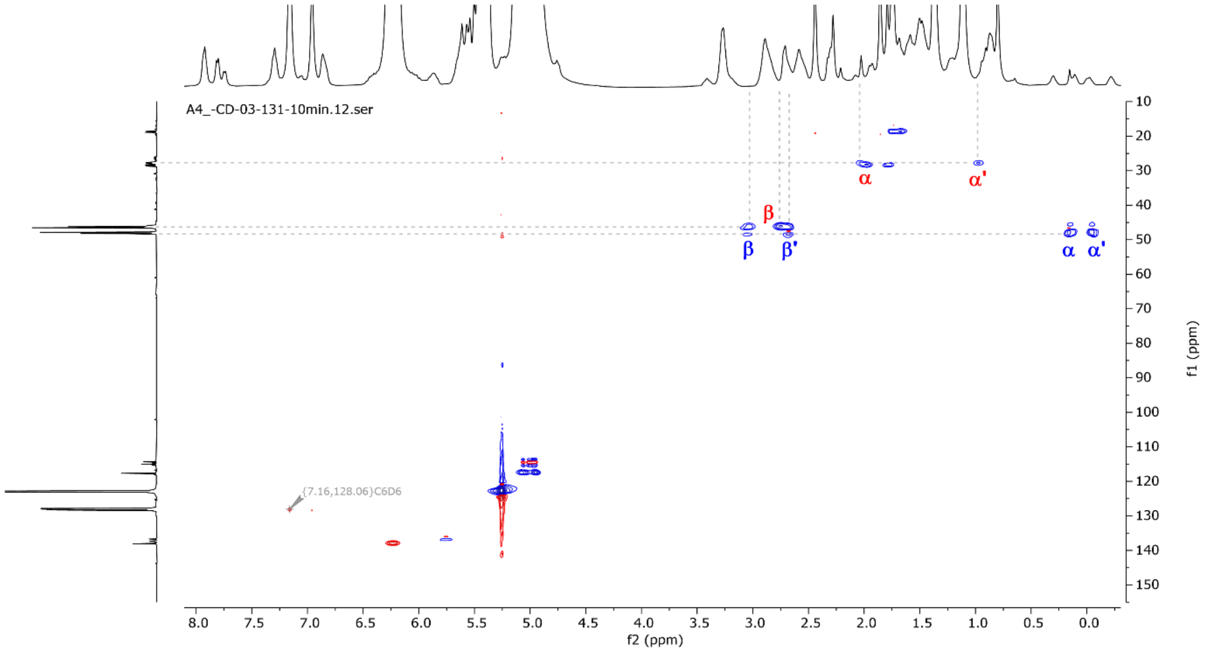

Figure S41. ^{13}C HSQC (101 MHz, benzene-d_6) spectrum of **7a** and **7b** generated with ^{13}C enriched ethylene.

In a nitrogen-filled glovebox, a solution of **1-CH_3** (0.009 mg, 0.022 mmol) in 500mg of benzene-*d*$_6$ was transferred to a J. Young tube. The tube was sealed, removed from the glovebox and frozen in liquid dinitrogen. The head-space was evacuated and butadiene or butadiene-*d*$_6$ (0.44 mmol) followed by ethylene (0.44 mmol) were added by vacuum transfer via a calibrated bulb. The tube was sealed under static vacuum, thawed, mixed by inversion for 10 minutes at room temperature and cooled down to 0 °C. 1H NMR spectrum was recorded at 0 °C and reveals the formation of the title compounds in >99% overall yield and ethane as a byproduct. 13C{1H} spectra were recorded at 0 °C. Butadiene fragment proton and carbon chemical shifts were determined by subtraction of the of the NMR spectra obtained with butadiene and butadiene-*d*$_6$ and are reported and Table 11.

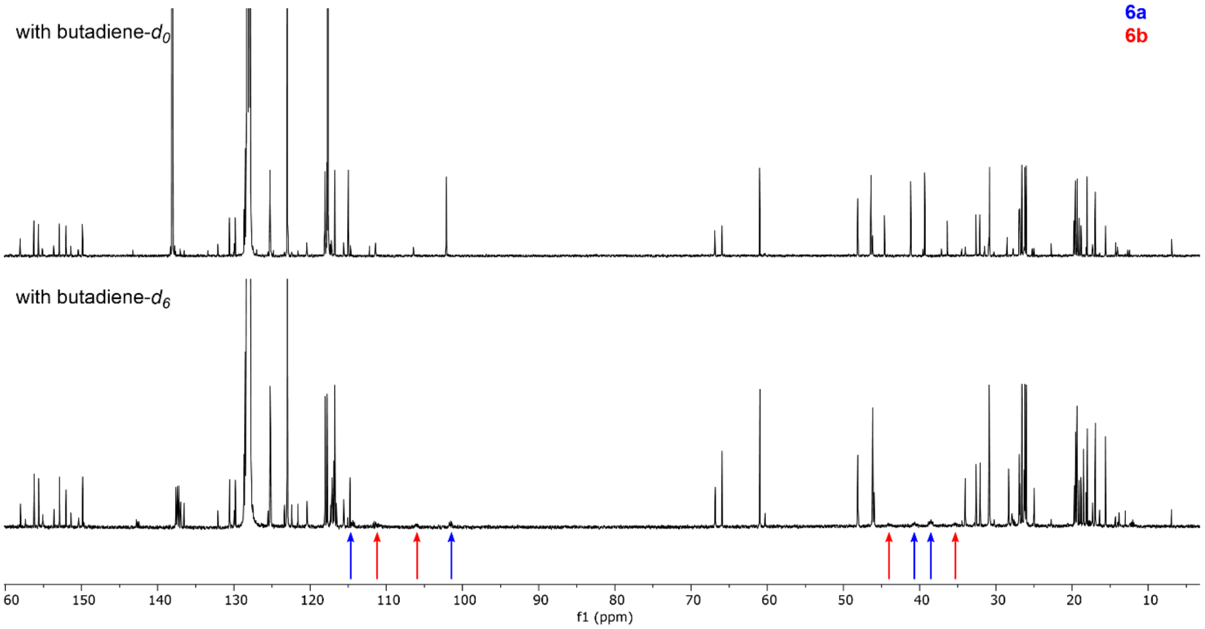

Figure S42. ^{13}C NMR (101 MHz, benzene-*d*$_6$) spectra of **7a** and **7b** with butadiene (top) and butadiene-d6 (bottom). Blue arrows indicate chemical shift of butadiene coordinated to **7a**. Red arrows indicate chemical shift of butadiene coordinated to **7b**.

Table 11: Chemical shifts of **7a** and **7b** metallacycle fragments.

		7a major isomer		7b minor isomer	
**fragment**	**position**	**^{1}H signals (ppm)**	**^{13}C signals (ppm)**	**^{1}H signals (ppm)**	**^{13}C signals (ppm)**
ethylene	α (2H)	-0.05 / 0.14	47.99	0.98 / 2.03	27.86
	β (2H)	2.68 / 3.04	46.40	2.76	46.17
butadiene	γ (2H)	2.88	39.34	2.59 / 2.68	36.36
	δ (1H)	5.63	114.99	5.62	106.40
	ε (1H)	5.60	102.12	5.87	111.47
	ζ (2H)	2.32 / 2.72	41.20	1.95 / 2.08	44.61

In a nitrogen-filled glovebox, a solution of **1-CH₃** (0.009 mg, 0.022 mmol) in 500mg of toluene-d_8 was transferred to a J. Young tube. The tube was sealed, removed from the glovebox and frozen in liquid dinitrogen. The head-space was evacuated and butadiene (0.44 mmol) followed by 13C enriched ethylene (0.44 mmol) were added by vacuum transfer via a calibrated bulb. The tube was sealed under static vacuum, thawed, mixed by inversion for 10 minutes at room temperature and cooled down to 0 °C. 1H NMR spectrum was recorded at 0 °C and reveals the formation of the title compounds in >99% overall yield and ethane as a byproduct. 13C{1H} and HBMC (constant13 = 15, 3 scans) spectra were recorded at 0 °C. Correlation signals between alpha and gamma-position of the metallacycle are found for **7a** and **7b**.

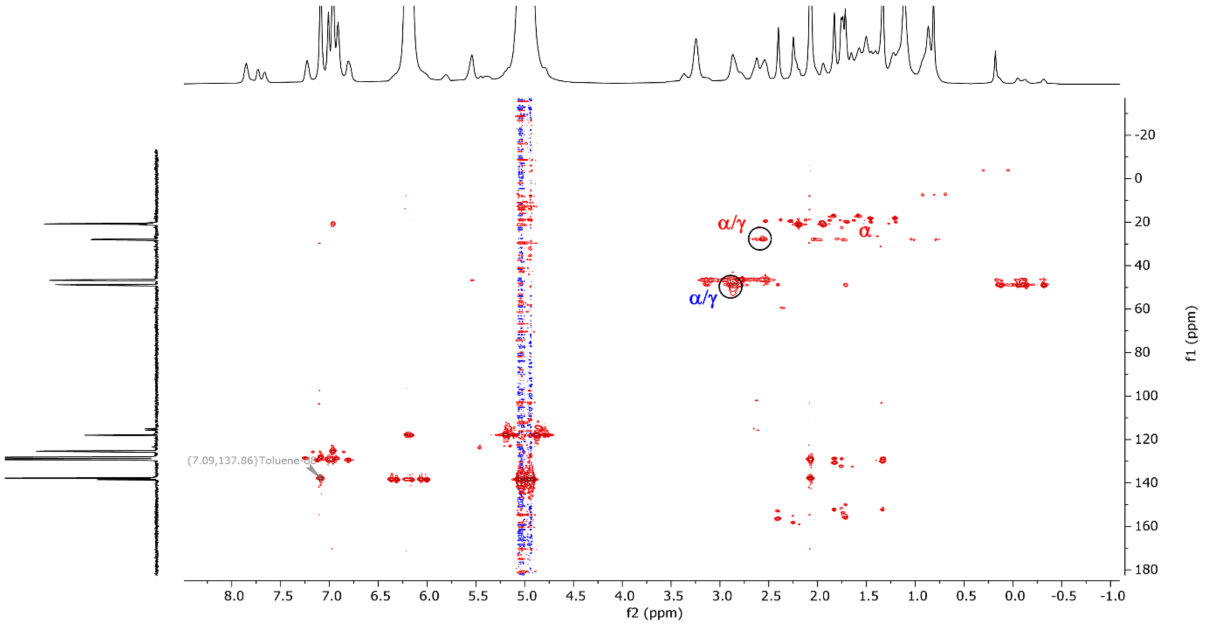

Figure S43. ^{1}H-^{13}C HMBC (101 MHz, toluene-d_8) spectrum of **7a** and **7b** generated with ^{13}C enriched ethylene. Correlation signals indicated in blue for **7a** and red for **7b**.

ii. Freeze-quenched Mössbauer Spectroscopy

In a nitrogen-filled glovebox, a solution of **1-CH₃** (0.046 mg, 0.11 mmol) in 500mg of benzene-d_6 was transferred to a J. Young tube. The tube was sealed, removed from the glovebox and frozen in liquid dinitrogen. The head-space was evacuated and butadiene (0.44 mmol) followed by ethylene (0.44 mmol) were added by vacuum transfer via a calibrated bulb. The tube was sealed under static vacuum, thawed and mixed by inversion for 10 minutes at room temperature. ^{1}H NMR spectrum was recorded to confirmed full conversion of **1-CH$_3$** to **7a** and **7b**. The tube was entered in the glovebox and the content transferred to a chilled Mössbauer cell.

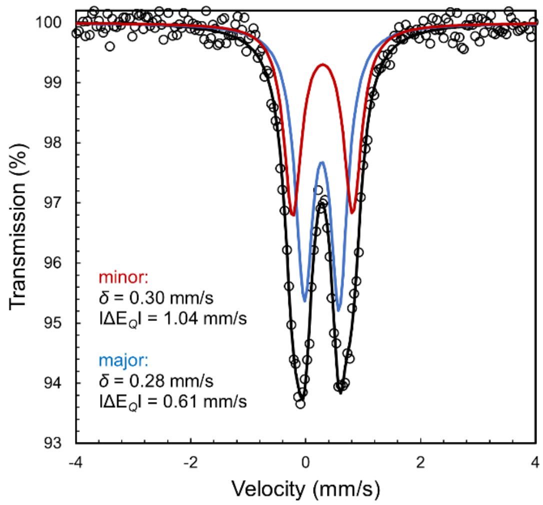

Figure S44. Zero-field 57Fe Mössbauer (freeze-quench, 80 K) spectrum of **7a** and **7b** after 10 minutes of reaction.

The same sample was prepared and the reaction allowed to run for one hour at room temperature. The tube was entered in the glovebox and the content transferred to a chilled Mössbauer cell.

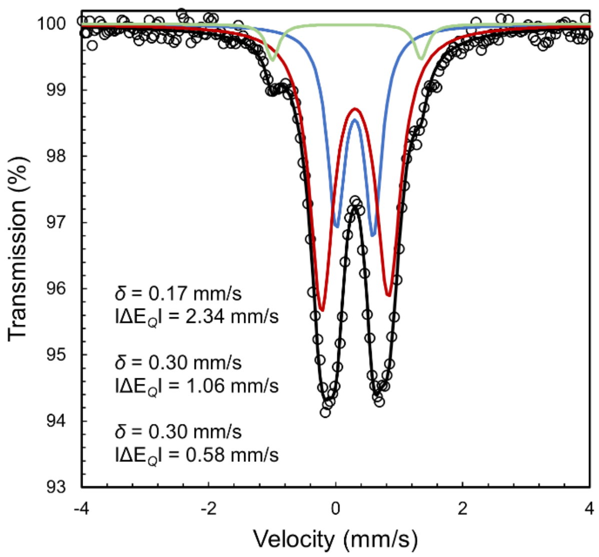

Figure S45. Zero-field 57Fe Mössbauer (freeze-quench, 80 K) spectrum of **7a** and **7b** after 60 minutes of reaction.

iii. X-ray diffraction analysis for 7a.

In a nitrogen-filled glovebox, a solution of **1-CH$_3$** (0.046 mg, 0.11 mmol) in 500mg of toluene-*d$_8$* was transferred to a J. Young tube. The tube was sealed, removed from the glovebox and frozen in liquid dinitrogen. The head-space was evacuated and butadiene (0.44 mmol) followed by ethylene (0.44 mmol) were added by vacuum transfer via a calibrated bulb. The tube was sealed under static vacuum, thawed and mixed by inversion for 10 minutes at room temperature. ^{1}H NMR spectrum was recorded to confirmed full conversion of **1-CH$_3$** to **7a** and **7b**. The tube was entered in the glovebox and the content transferred to a chilled 20 ml scintillation vial, topped with cold hexanes and kept at -35 °C. Recrystallization over six days at -35 °C afforded suitable crystals for single-crystal X-ray diffraction analysis. The structure and coordinates were deposited with the Cambridge Crystallographic Data Center (CCDC# 2256990).

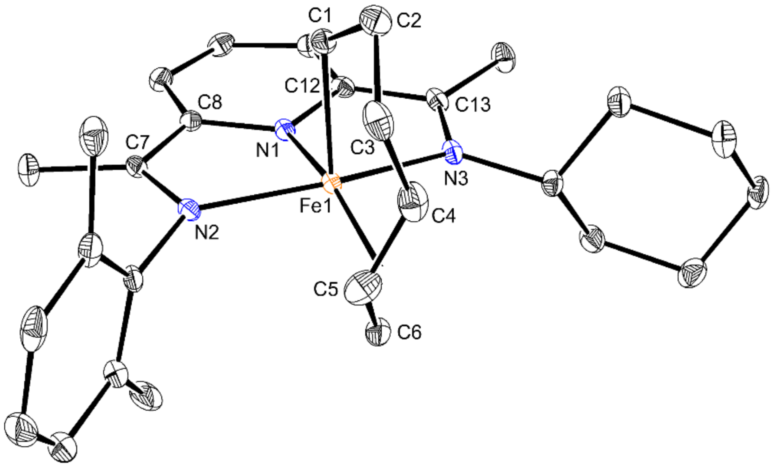

Figure S46. solid-state structure of **7a**, depicted with **30**% probability ellipsoids (CCDC # 2256990). Hydrogen atoms are omitted for clarity. C = gray, N = blue, Fe = red-orange.

Table S12: Experimental crystal data for **7a**.

Crystal data	
Chemical formula	$C_{29}H_{39}FeN_3 \cdot 0.5(C_{29}H_{38}FeN_3)$
M_r	727.72
Crystal system, space group	Monoclinic, $P2_1/c$
Temperature (K)	100
a, b, c (Å)	10.8686 (2), 28.2334 (6), 24.4611 (5)
β (°)	95.818 (1)
V (Å^3)	7467.4 (3)
Z	8
Radiation type	Cu $K\alpha$
μ (mm^{-1})	5.01
Crystal size (mm)	0.19 × 0.09 × 0.05
Data collection	
Diffractometer	Bruker *APEX*-II CCD
Absorption correction	Multi-scan *SADABS2016*/2 (Bruker,2016/2) was used for absorption correction. wR2(int) was 0.1176 before and 0.0657 after correction. The Ratio of minimum to maximum transmission is 0.8290. The λ/2 correction factor is Not present.
T_{min}, T_{max}	0.624, 0.753
No. of measured, independent and observed [$I > 2\sigma(I)$] reflections	82134, 13175, 10596
R_{int}	0.078

$(\sin \theta/\lambda)_{max}$ (Å^{-1})	0.596
Refinement	
$R[F^2 > 2\sigma(F^2)]$, $wR(F^2)$, S	0.052, 0.146, 1.02
No. of reflections	13175
No. of parameters	921
No. of restraints	3
H-atom treatment	H atoms treated by a mixture of independent and constrained refinement
	$w = 1/[\sigma^2(F_o^2) + (0.0732P)^2 + 11.2005P]$ where $P = (F_o^2 + 2F_c^2)/3$
$\Delta\rangle_{max}$, $\Delta\rangle_{min}$ (e Å^{-3})	1.15, -0.97

iv. Variable Temperature ^{1}H NMR Spectroscopy

In a nitrogen-filled glovebox, a solution of **1-CH$_3$** (0.009 mg, 0.022 mmol) in 500mg of toluene-d_8 was transferred to a J. Young tube. The tube was sealed, removed from the glovebox and frozen in liquid dinitrogen. The head-space was evacuated and butadiene (0.44 mmol) followed by ^{13}C enriched ethylene (0.44 mmol) were added by vacuum transfer via a calibrated bulb. The tube was sealed under static vacuum, thawed, mixed by inversion for 10 minutes at room temperature and cooled down to -78 °C. The sample was analyzed by 1H NMR spectroscopy at -80 °C then by 10 °C increasing increments to 30 °C. 5-minute intervals were allowed between scans to equilibrate the solution temperature. The compiled data are reported in Figure S47.

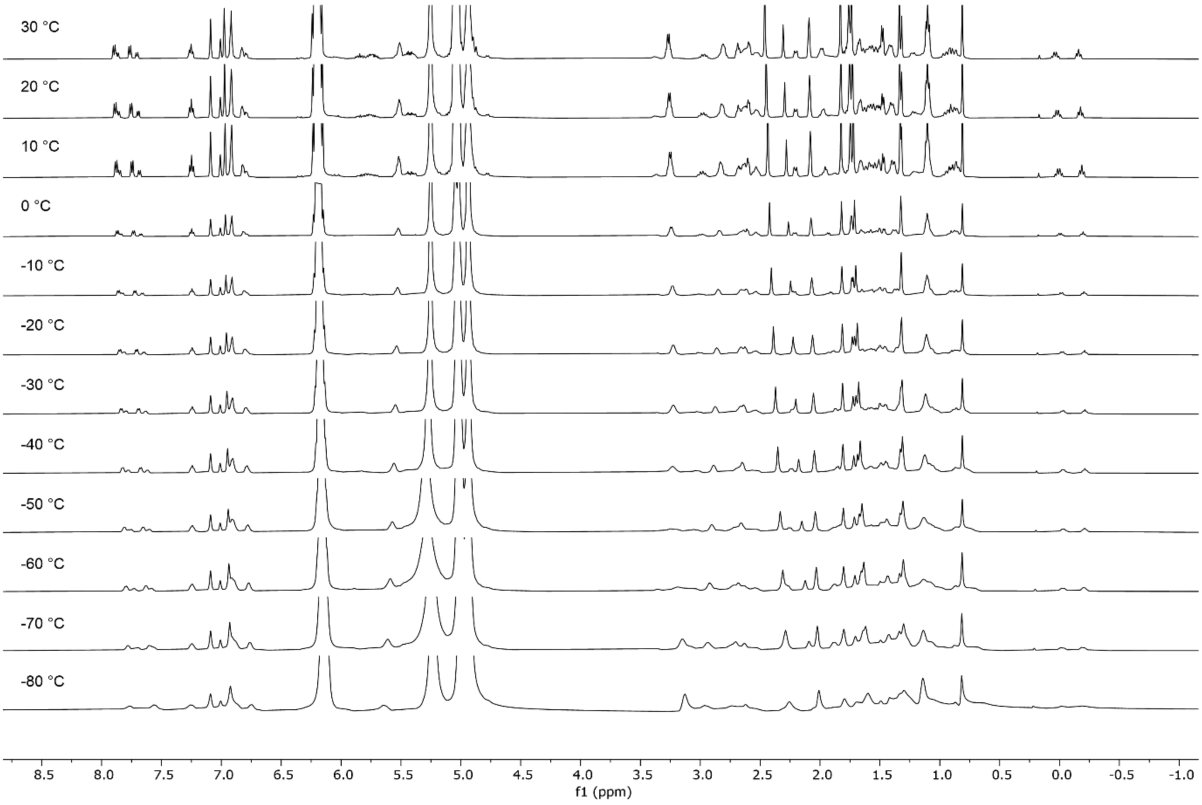

Figure S47. Variable temperature ^{1}H NMR (400 MHz, toluene-d_8) spectrum of **7a** and **7b.**

V. Deuterium Labeling Experiments

i. Cross-over experiment with butadiene and ethylene/ethylene-d_4

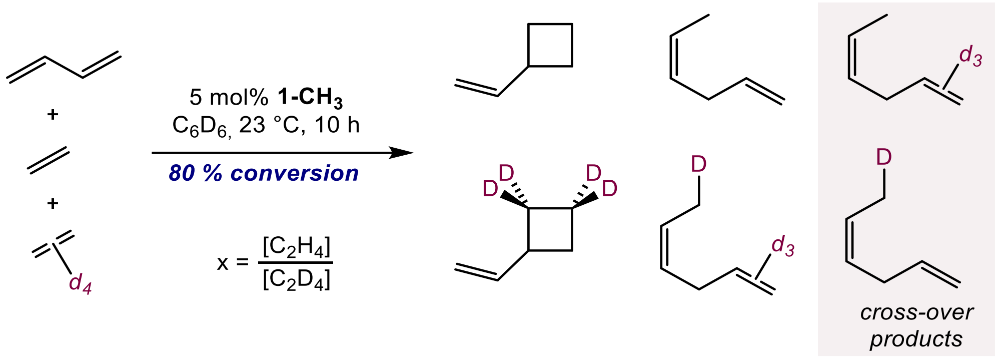

In a nitrogen-filled glovebox, a solution of **1-CH₃** (0.009 mg, 0.022 mmol) in 500 mg of benzene-d_6 transferred to a J. Young tube. The tube was sealed, removed from the glovebox and frozen in liquid dinitrogen. The head-space was evacuated and butadiene (0.44 mmol) followed by ethylene (0.44 mmol) and ethylene-d_4 (0.22 mmol) were added by vacuum transfer via a calibrated bulb. The tube was sealed under static vacuum, thawed and mixed by inversion at room temperature until 80% conversion of butadiene to vinylcyclobutane and (*Z*)-hexa-1,4-diene was observed. The volatiles of the reaction were transferred to an other J. Young tube and then analyzed by 1H and 13C{1H} NMR spectroscopy. In parallel, a sample was prepared in the similar manner with solely ethylene-d_4 (0.44 mmol) giving a sample of the isotopologue 1,4-HD-d_4. Entrainment of the deuterium label to the C4 terminus and retention at the olefinic position was identified through the combination 1H NMR and quantitative 13C NMR spectroscopy in comparison with the spectra for natural abundance (*Z*)-hexa-1,4-diene (1,4-HD) and 1,4-HD-d_4. Only two isotopologs of 1,4-HD, which were spectroscopically consistent with 1,4-HD-d_0 and 1,4-HD-*d4*, were observed.

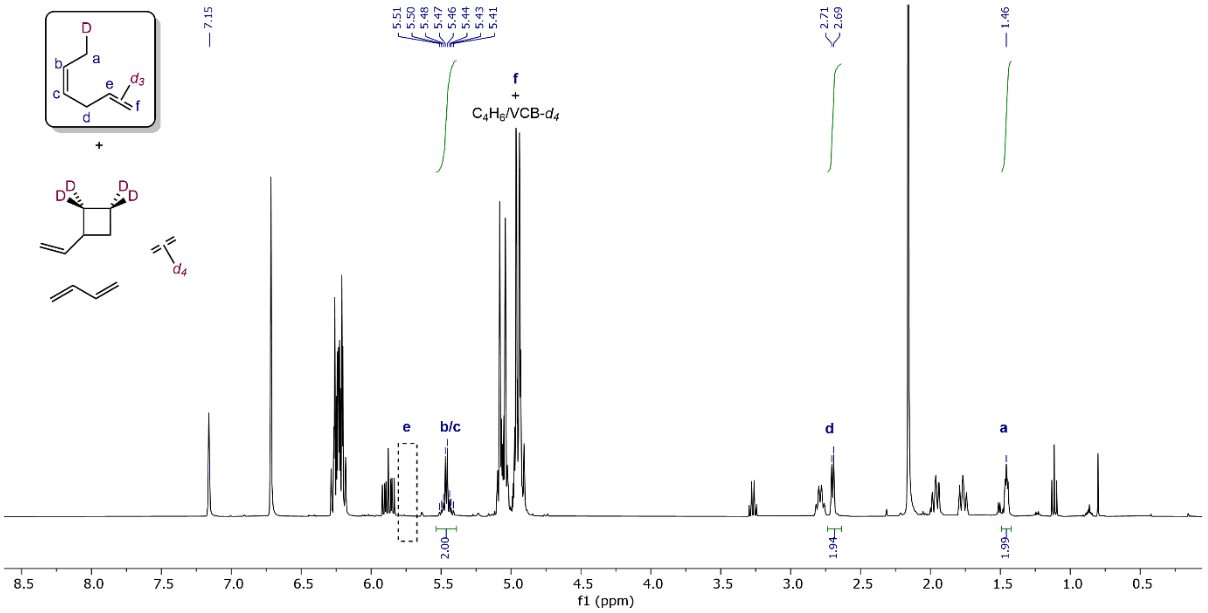

Figure S48. ^{1}H NMR (400 MHz, benzene-d_6) spectrum of (*Z*)-hexa-1,4-diene-d_4. Assignment of the signals corresponding to (*Z*)-hexa-1,4-diene are given.

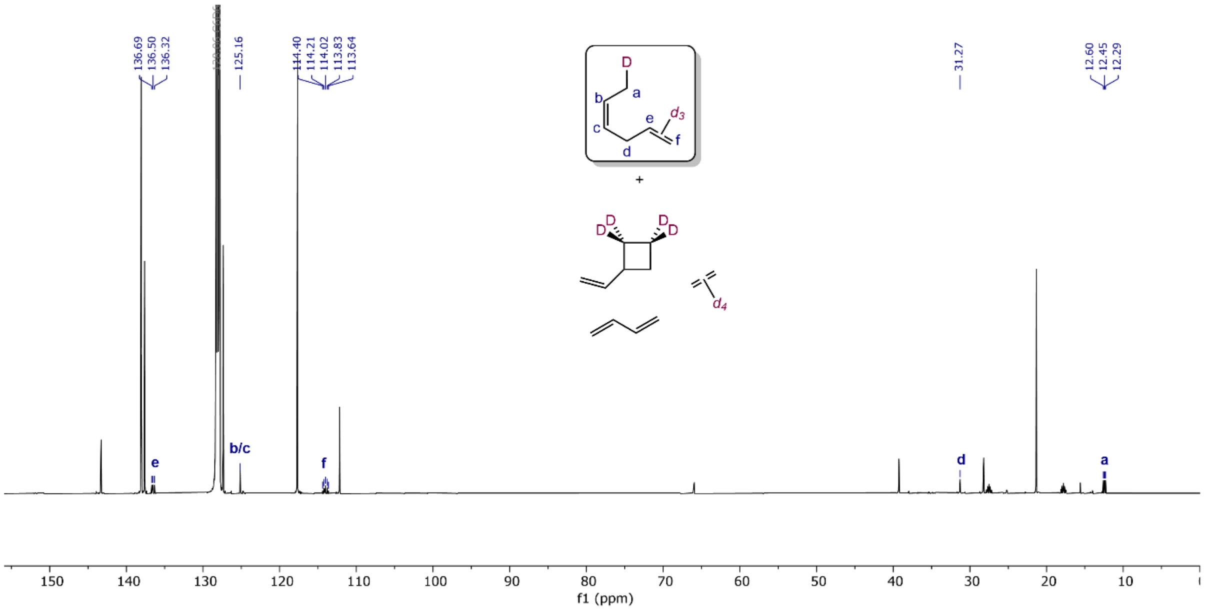

Figure S49. ^{13}C NMR (101 MHz, benzene-d_6) spectrum of (*Z*)-hexa-1,4-diene-d_4. Assignment of the signals corresponding to (*Z*)-hexa-1,4-diene are given.

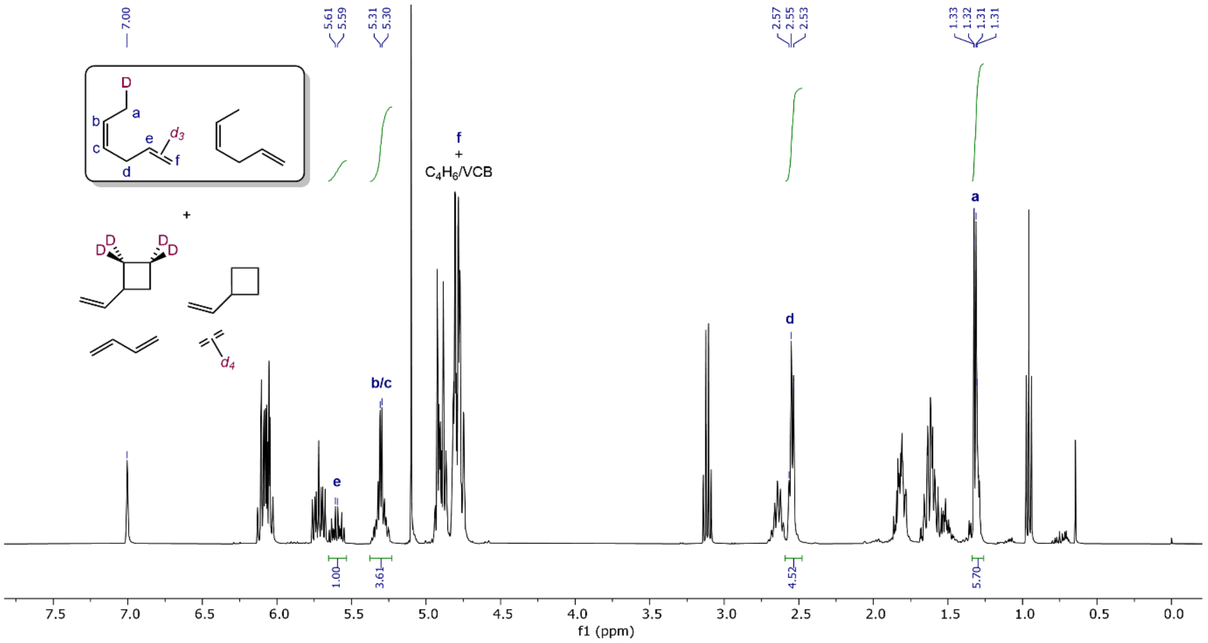

Figure S50. ^{1}H NMR (400 MHz, benzene-d_6) spectrum of the reaction run with an equimolar amount of ethylene and ethylene-d_4. Assignment of the signals corresponding to (*Z*)-hexa-1,4-diene are given.

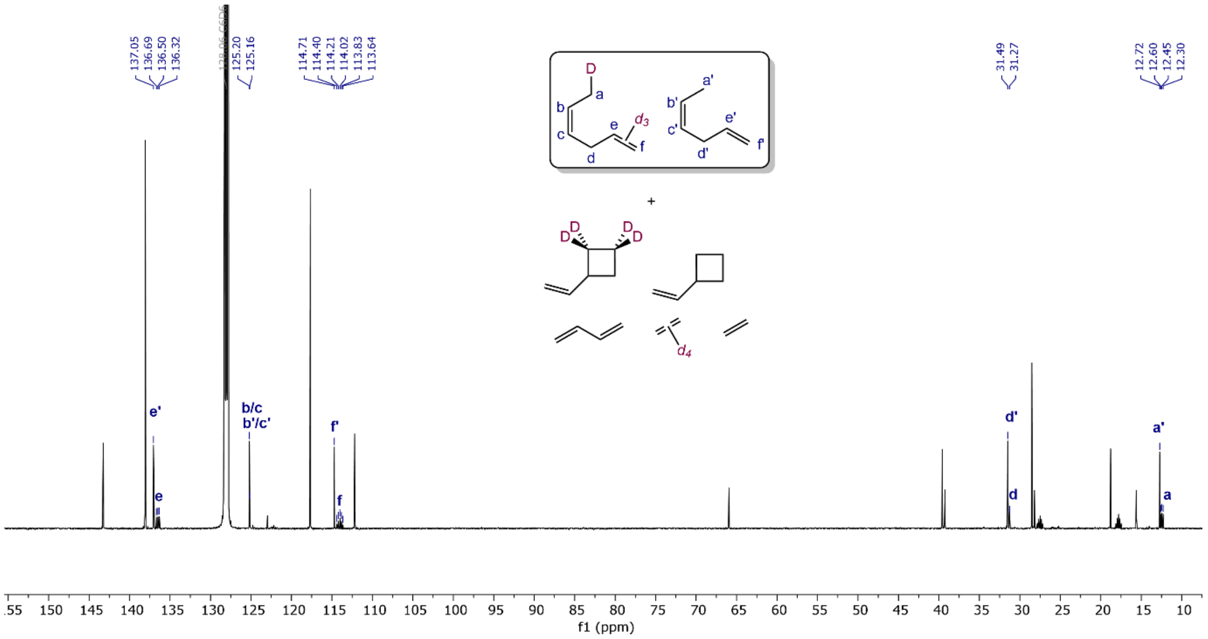

Figure S51. ^{13}C NMR (101 MHz, benzene-d_6) spectrum of the reaction run with an equimolar amount of ethylene and ethylene-d_4.

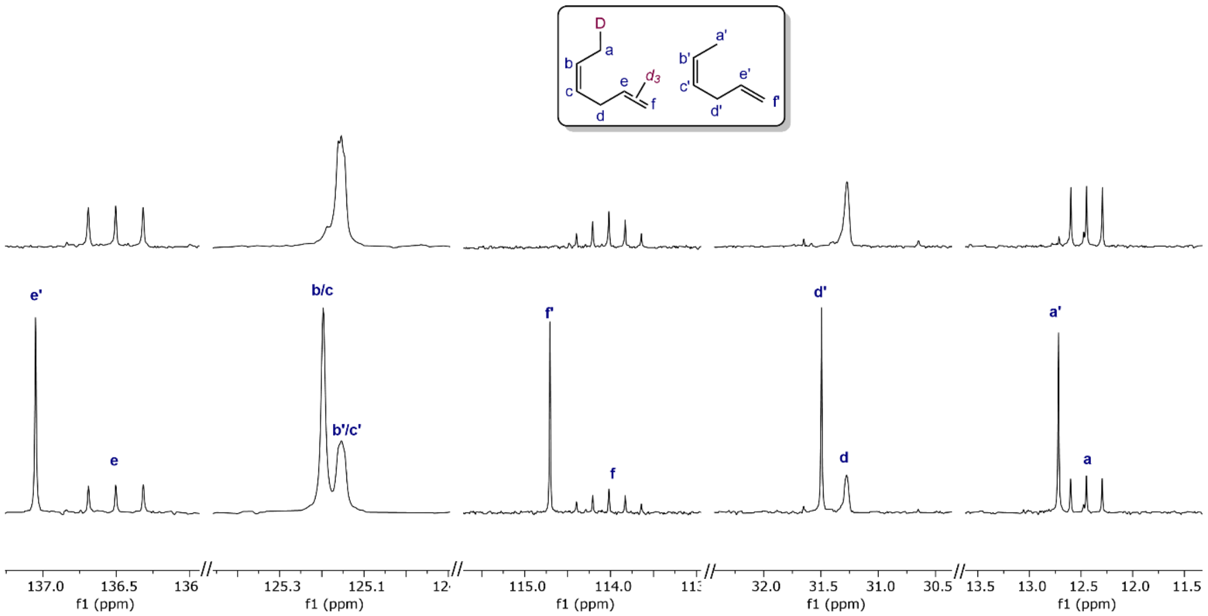

Figure S52. Expanded view of the overlay of the ^{13}C NMR (101 MHz, benzene-*d*$_6$) spectra of the reaction run with ethylene- *d*$_4$ (top) and an equimolar amount of ethylene and ethylene-*d*$_4$ (bottom).

In order to record a GC-HRMS chromatogram, the same protocol was run in mesitylene as the solvent of the reaction. After 14h, the reaction was filtered over a plug of silica to remove traces of catalyst. Both reaction solutions, run with ethylene- *d*$_4$ and an equimolar mixture of ethylene and ethylene-*d*$_4$, were analyzed by GC-HRMS using mesitylene as solvent, with a solvent delay of 1 minute and switching off the detector at t = 5 minutes. pentane, dichloromethane, benzene, toluene or dodecane could not be used as reaction solvent or GC sample solvent as it would interfere with the retention time of C6 volatile fraction or its mass spectrum.

A distribution of the *d*$_1$, *d*$_2$, *d*$_3$ isotopologues was observed while only the *d*$_4$ isotopologues was expected when using only ethylene- *d*$_4$. Analysis of the HRMS trace of the recovered PDI ligand following the completion of the reaction with ethylene-*d*$_4$ gave a mixture of isotopologues, consistent with a background H/D scrambling process (see below). The distribution pattern experimentally observed is therefore a result of either: (*i*) a competitive (PDI) to olefin substrates ligand-to-ligand hydride transfer; (ii) a low resolution between fragmentation and isotopologues mass peaks at this mass range. The comparison between

the GC-HRMS spectra of the two experiments rules out 1,4-hexadiene-d_1, -d_2 and -d_3 isotologues formation due to a Cossee-Arlman mechanism. For better visualization, subtraction of HRMS spectrum (experimental x=0) to spectrum (experimental x=1) was provided in the main text of the paper.

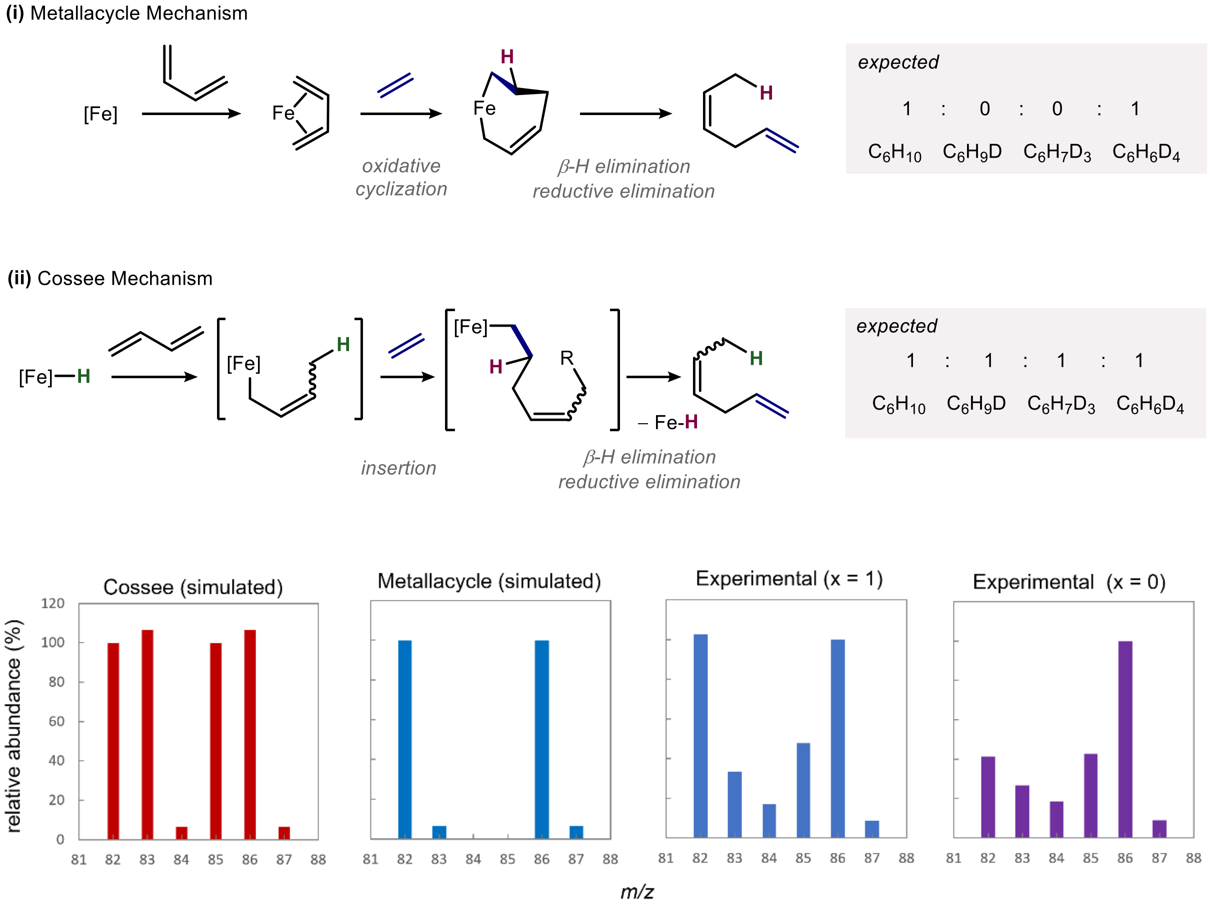

Figure S53. Crossover experiment isotopolog distributions expected for a catalyst system operating by either of two mechanistic possibilities, compared with experimental HRMS(EI) results. x corresponds to the ratio of ethylene-d_0 to ethylene-d_4.

ii. H/D Kinetic Isotopic Effect Measurement

In a nitrogen-filled glovebox, a solution of **1-CH$_3$** (0.009 mg, 0.022 mmol) in 500 mg of benzene-d_6 transferred to a J. Young tube. The tube was sealed, removed from the glovebox and frozen in liquid dinitrogen. The head-space was evacuated and butadiene (0.44 mmol) followed by ethylene (0.44 mmol) or ethylene-d_4 (0.44 mmol) were added by

vacuum transfer via a calibrated bulb. The tube was sealed under static vacuum, thawed and mixed by inversion at room temperature while 1H NMR spectra were recorded throughout the course of the reaction. Relative concentration of each products were determined by comparing 1H NMR integration of the products to butadiene. Each run was performed in duplicate.

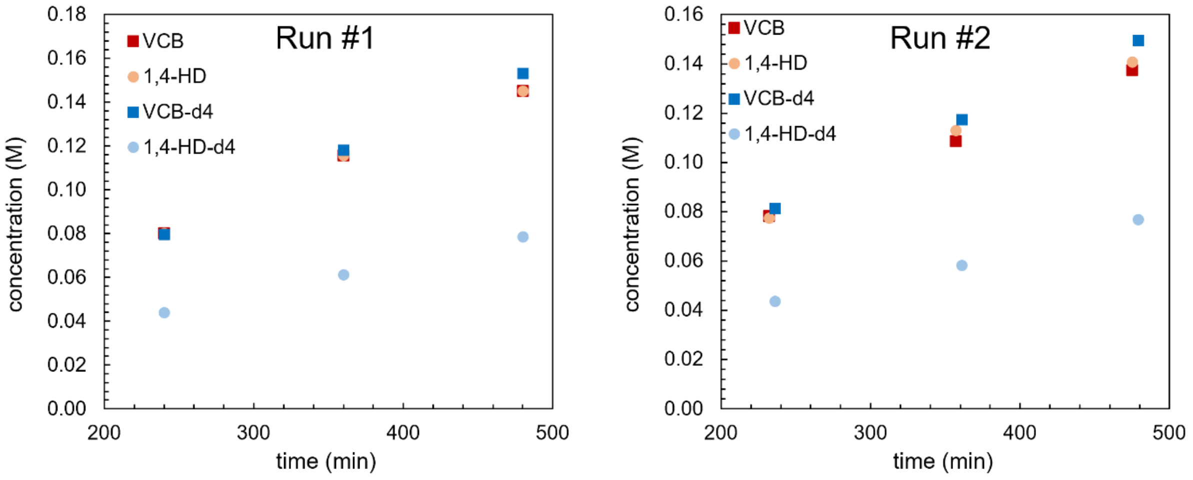

Figure S54. Time courses for the formation of vinylcyclobutane (VCB) and (Z)-hexa-1,4-diene (1,4-HD) with ethylene or ethylene-d_4.

Table S13. Calculations of the H/D kinetic isotope effect for the iron-catalyzed competitive formation of vinylcyclobutane and (*Z*)-hexa-1,4-diene.

Run	VCB	k (10^{-4} s^{-1})	k_H/k_D	Average	Run	1,4-HD	k (10^{-4} s^{-1})	k_H/k_D	Average
#1	d0	2.70	0.88	0.88(1)	#1	d0	2.70	1.86	1.89(1)
	d4	3.07				d4	1.45		
#2	d0	2.44	0.87		#2	d0	2.61	1.92	
	d4	2.80				d4	1.36		

iii. (PDI) to Substrates H/D Scrambling Evidence

At the end of the time course both reaction tubes (with ethylene-d_0 and ethylene-d_4) were opened and the solutions were exposed to air for 24h. After this time the solutions were filtered over a plug of celite and LC-MS were recorded showing presence of the free ligand ($^{Cy}A^{Me}$PDI) in both samples. MS spectra indicates deuterium incorporation in the backbone of the ligand for the reaction run with ethylene-d_4.

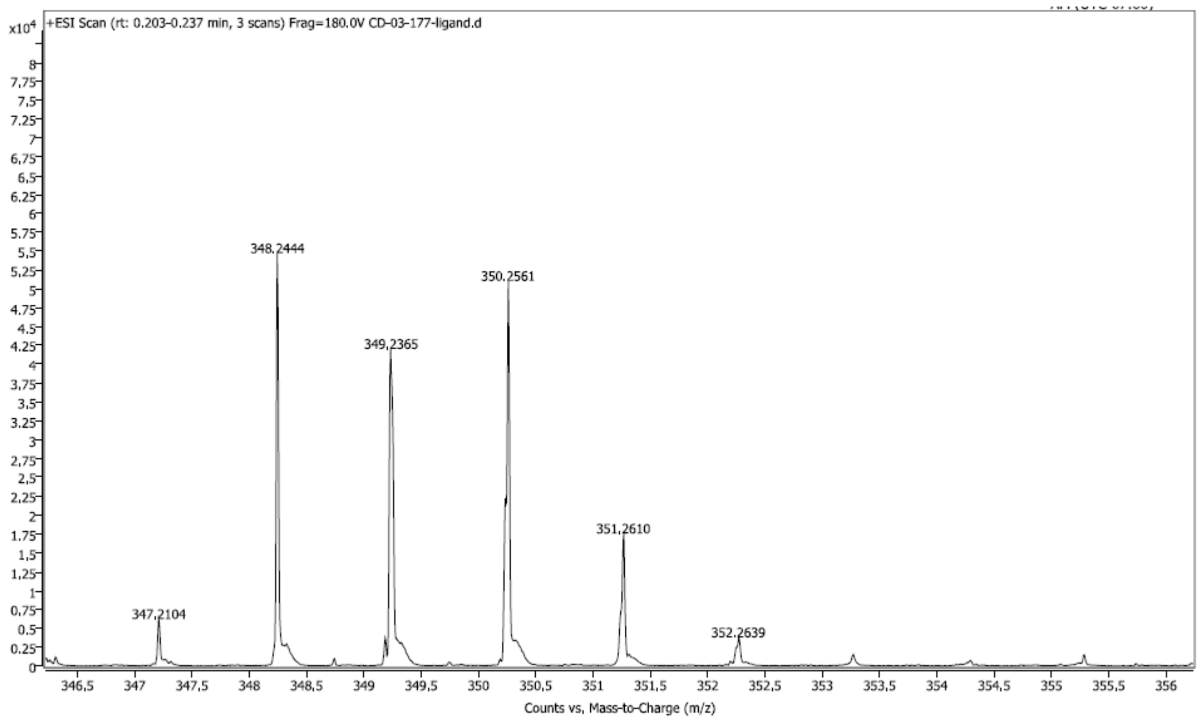

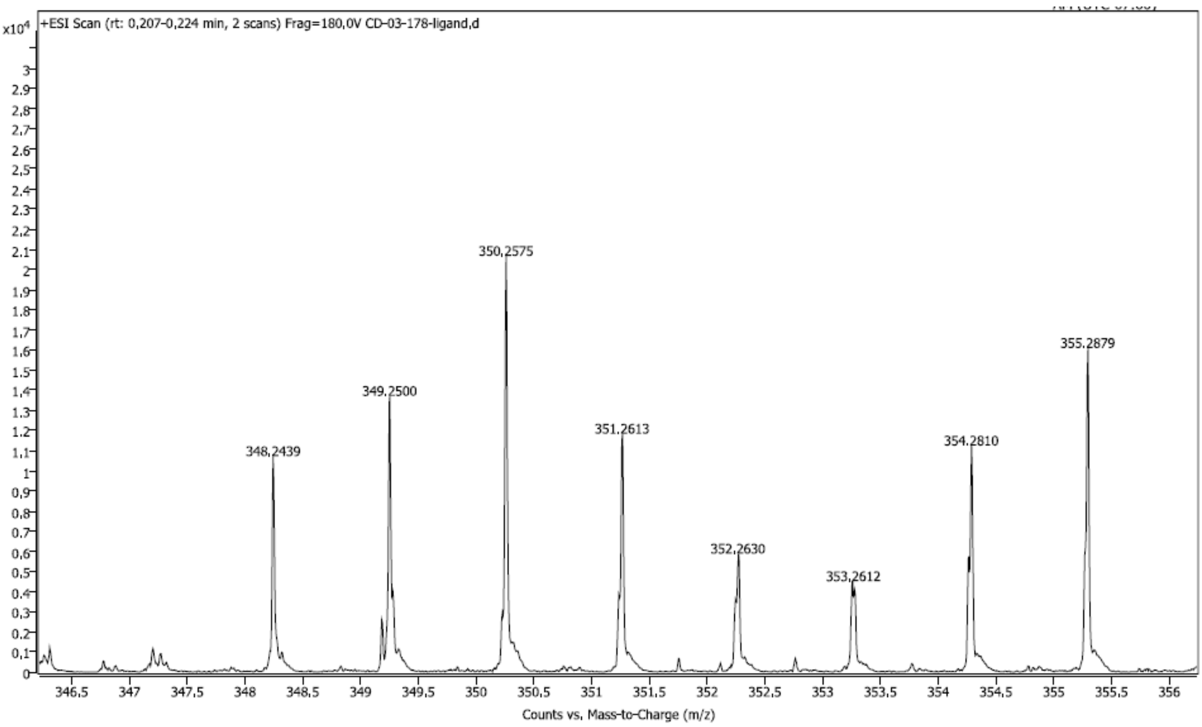

Figure S55. LCMS of ($^{Cy}A^{Me}$PDI) after reaction with butadiene and ethylene-d_0 (top) or ethylene-d_4 (bottom).

iii. ^{13}C-labeled ethylene experiment to study the C-C reductive elimination step

In a nitrogen-filled glovebox, a solution of [Fe] precatalyst (0.022 mmol) in 500mg of benzene-d_6 was transferred to a J. Young tube. The tube was sealed, removed from the glovebox and frozen in liquid dinitrogen. The head-space was evacuated and butadiene (0.44 mmol) followed by ^{13}C-labeled ethylene (0.44 mmol) were added by vacuum transfer via a calibrated bulb. The tube was sealed under static vacuum, thawed and mixed by inversion for 10 minutes at room temperature. Time-course of the reaction were measured by 1H and 13C{1H} NMR spectroscopy with precatalysts [(MePDI)Fe(N_2)]$_2$(μ-N_2) (Figure S56 and S57) and **1-CH₃** (Figure S58 and S59). Conversion of butadiene were calculated by ratio analysis of the peaks at 6.23 ppm (C_4H_6, 2H) and 5.88 ppm (VCB, 1H or VCB, 1H + 1,4-HD, 1H)). ^{13}C incorporation of the gamma-position of the in-situ formed metallacycles were monitored during these time-courses. The volatiles of the reaction were isolated from the reaction mixture by vacuum transfer into a J. Young tube. The tube containing the reaction mixture was placed in a room temperature water bath during the vacuum transfer. The volatiles of the reaction were then analyzed by 13C{1H} NMR and stacked to the time-course spectra.

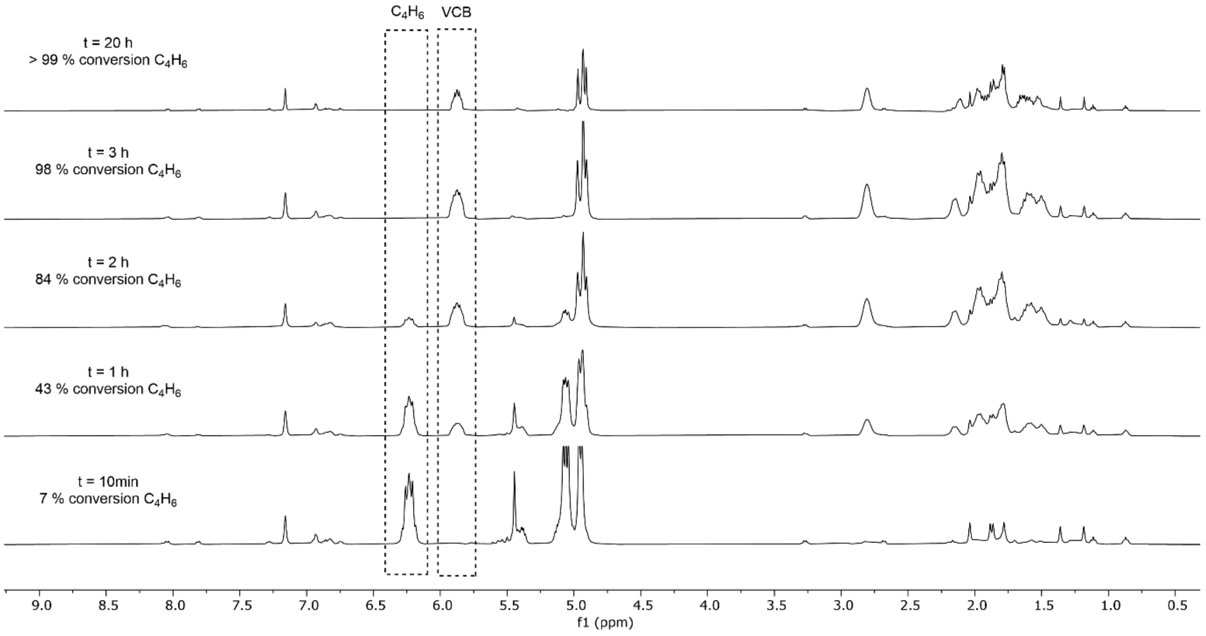

Figure S56. ^{1}H NMR (400 MHz, benzene-d_6) spectrum of the time-course with [Fe] = ½ [(MePDI)Fe(N_2)]$_2$(μ-N_2).

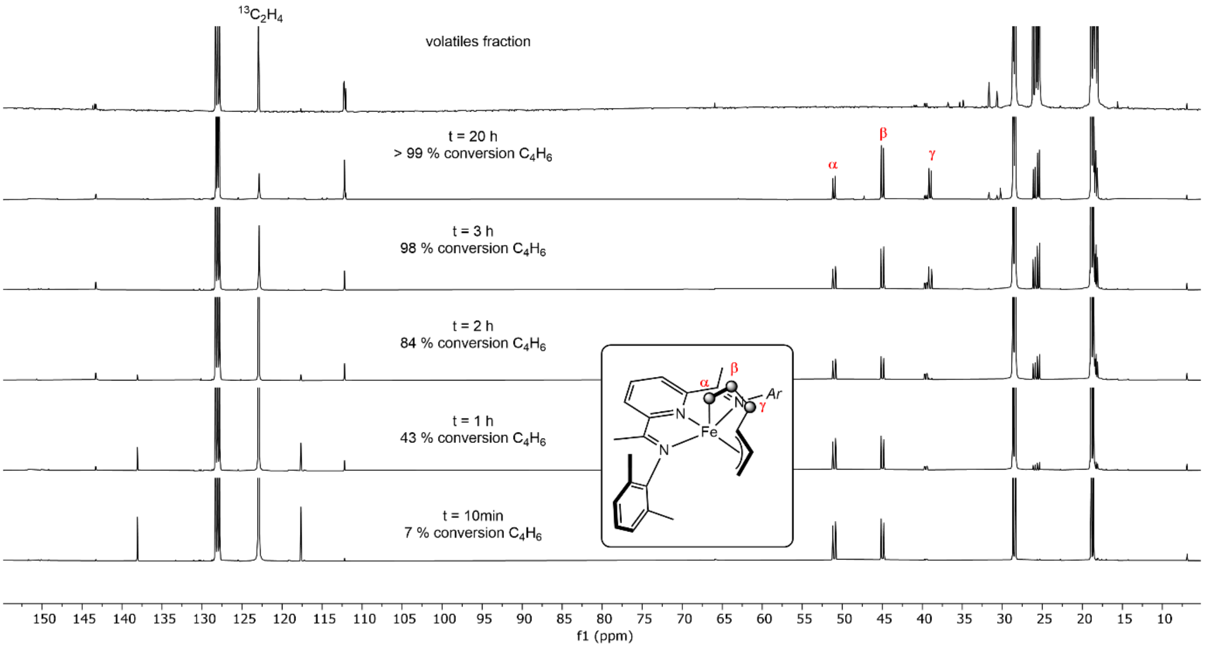

Figure S57. ^{13}C NMR (101 MHz, benzene-d_6) spectra stacking of the time-course with [Fe] = ½ [(MePDI)Fe(N_2)]$_2$(μ-N_2). Assignment of the signals corresponding to the ^{13}C-labeled carbons of the metallacycle are given.

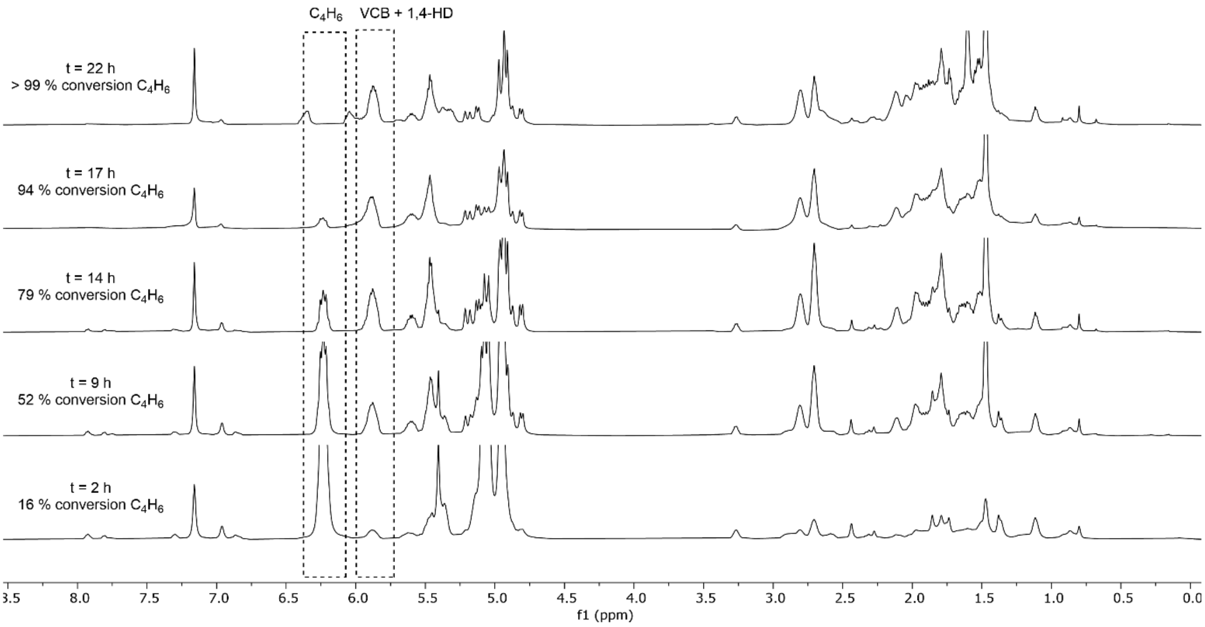

Figure S58. ^{1}H NMR (400 MHz, benzene-d_6) spectrum of the time-course with [Fe] = **1-CH$_3$**.

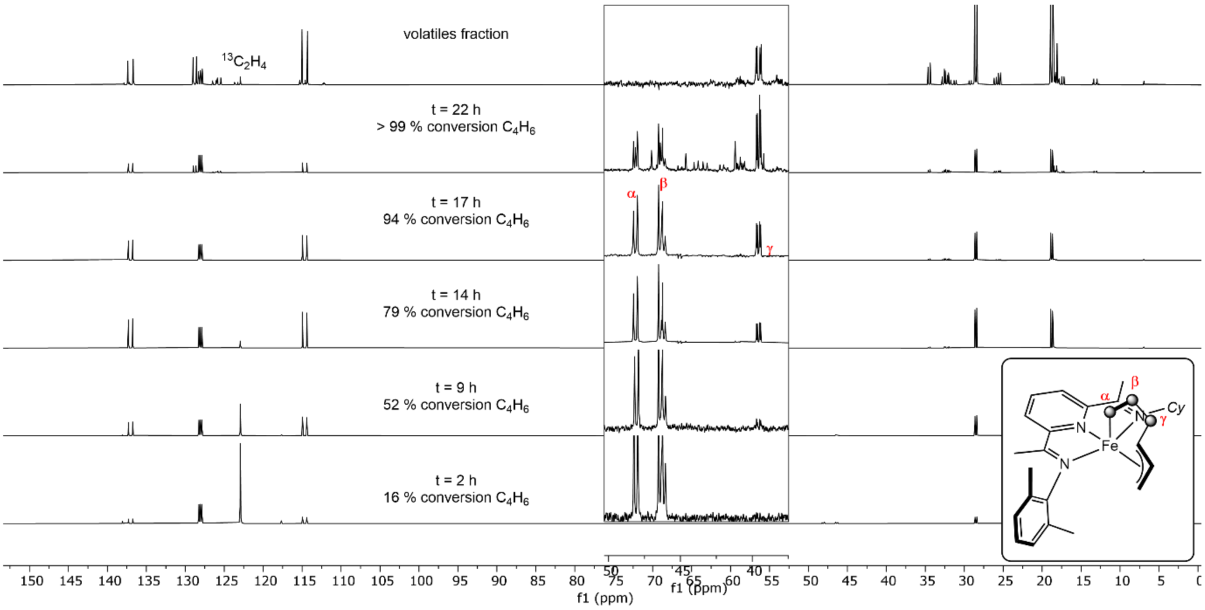

Figure S59. ^{13}C NMR (101 MHz, benzene-d_6) spectra stacking of the time-course with [Fe] = **1-CH$_3$**. Assignment of the signals corresponding to the ^{13}C-labeled carbons of the metallacycle are given.

VII. Computational Analyses

i. Computational Details

All DFT calculations were performed with the ORCA package in the gas phase if not otherwise specify.[viii] Geometry optimizations and single-point calculations were carried out at the B3LYP[ix,x,xi] or TPSSh[xii,xiii] level of DFT. The B3LYP hybrid functional often outperforms pure gradient-corrected functionals in the accurate representation of transition metal complexes, especially those involving significant metal–ligand covalency.[xiv] The TPSSh hybrid meta-GGA functional has given accurate representation of 1st-row transition metal complexes involved into spin-cross-over (SCO) events.[xv,xvi] Alrichs' all-electron Gaussian basis sets were employed for all calculations with B3LYP level of DFT,[xvii,xviii,xix] wherein the triple-ζ basis set def2-TZVP, which includes one set of polarization functions, was used to describe metal atoms and all atoms directly coordinated to a metal center. The double-ζ basis set def2-SV(P), which includes one set of polarizing *d*-functionals on all non-hydrogen atoms, was used for all other atoms. Auxiliary basis sets were chosen to match the orbital basis.[xx,xxi,xxii] For the TPSSh functional results, calculations with the triple-ζ basis set def2-TZVP for all atoms were done. The RIJCOSX approximation was used to accelerate the calculations.[xxiii,xxiv,xxv] the For the single point calculations at the TPSSh, level Scalar relativistic effects were included using the Douglas–Kroll–Hess Hamiltonian (DKH)[xxvi,xxvii,xxviii,xxix], dispersion corrections (D3BJ) were applied and the conductor-like polarizable continuum (CPCM) model was used to describe solvation effects. Benzene (ε = 2.28) was chosen as solvent. Throughout this manuscript, computational results are described using the broken symmetry approach introduced by Ginsberg[xxx] and Noodleman *et al*.[xxxi] Because several broken symmetry solutions are spin-unrestricted Kohn-Sham equations may be obtained, the general notation for broken symmetry (*m,n*)[xxxii] has been adopted, where *m* (*n*) denotes the number of spin-up (spin-down) electrons at the two interacting fragments.[xxxiii] Representations of canonical orbitals and the corresponding spin density plots were generated with the program *Chimera*.[xxxiv]

ii. ($^{Cy}A^{Me}$PDI)FeCH$_3$ (1-CH$_3$)

Geometry constraint of the Fe-N bond lengths were applied to match the experimental structure data and the spectroscopic Mössbauer parameters.

Input File for Geometry and Frequency Analysis

```
! UKS B3LYP RIJCOSX def2-SVP def2/J Normalprint SlowConv TightSCF TightOpt UCO
NumFreq Grid6 NoFinalGrid

%output
Print[P_Basis]2
Print[P_MOs]1
end

%pal nprocs 16
        end

%SCF BrokenSym 4,1
         MaxIter 5000
    TolE 1e-7
    TolErr 1e-6
   end

%geom Constraints
        { B 0 2 2.114 C }
        { B 0 1 2.131 C }
        end
        end

%basis NewGTO 26 "def2-TZVP(-f)" end
          NewGTO 7 "def2-TZVP(-f)" end
     NewAuxGTO 26 "def2/J" end
          NewAuxGTO 7 "def2/J" end
    end

*xyz 0 4
Fe1     9.9066420000    3.4385950000    8.9612110000
N2     10.3201020000    5.2493440000   10.0050310000 NewGTO "def2-TZVP(-f)" end
NewAuxGTO "def2/J" end
N3      9.7846850000    2.7258140000    6.9748310000 NewGTO "def2-TZVP(-f)" end
NewAuxGTO "def2/J" end
N4     11.5332150000    4.1576260000    8.0796260000 NewGTO "def2-TZVP(-f)" end
NewAuxGTO "def2/J" end
C5      9.1512990000    2.0244250000   10.2427580000
C6      9.5745980000    5.0068720000   12.3558040000
C7      8.6802850000    5.6191830000   13.4344670000
C8      7.2352070000    5.7188620000   12.9606470000
C9      7.1392860000    6.5012600000   11.6493380000
C10     8.0307540000    5.8849930000   10.5728540000
C11     9.4764630000    5.8161670000   11.0626430000
C12    10.3248580000   -0.0696170000    7.5239620000
```

```
C13     7.3952540000     3.6184910000     5.6597120000
C14     9.0293150000     0.3971320000     6.9102280000
C15     8.0068710000    -0.5118420000     6.6563500000
C16     6.8131440000    -0.1099630000     6.0803620000
C17     6.6248820000     1.2167120000     5.7415560000
C18     7.6099680000     2.1676140000     5.9996070000
C19     8.8114080000     1.7491220000     6.5980980000
C20     12.2138090000     6.6760930000     10.7786410000
C21     11.0899720000     2.1866000000     4.9247550000
C22     11.5535900000     5.6733740000     9.8685650000
C23     12.2347570000     5.1621650000     8.6873720000
C24     13.3899050000     5.6650670000     8.1197310000 NewGTO "def2-TZVP(-f)" end
NewAuxGTO "def2/J" end
C25     13.7848910000     5.2054380000     6.8536090000
C26     12.9954110000     4.2822240000     6.1796260000
C27     11.8513730000     3.7744960000     6.8031600000
C28     10.8794190000     2.8764400000     6.2409630000
H29     9.8291130000     1.7688760000     10.8727160000
H30     8.3981520000     2.3891690000     10.7135840000
H31     8.8730320000     1.2545340000     9.7416990000
H32     9.3016100000     4.0915930000     12.1880890000
H33     10.4943850000     4.9962080000     12.6637240000
H34     9.0071100000     6.5041240000     13.6598180000
H35     8.7201400000     5.0720270000     14.2344270000
H36     6.8759760000     4.8269680000     12.8313120000
H37     6.7027990000     6.1611500000     13.6400190000
H38     7.4078000000     7.4204470000     11.8023550000
H39     6.2190940000     6.5042740000     11.3425260000
H40     7.7159870000     4.9920860000     10.3606780000
H41     7.9852050000     6.4207970000     9.7656710000
H42     9.7833290000     6.7287170000     11.2458930000
H43     11.0432270000     0.0756690000     6.9040210000
H44     10.4930660000     0.4234520000     8.3301460000
H45     10.2631530000    -1.0057020000     7.7283710000
H46     7.4021440000     4.1405070000     6.4656240000
H47     8.0978350000     3.9187300000     5.0784250000
H48     6.5488080000     3.7224420000     5.2186840000
H49     8.1274930000    -1.4070660000     6.8777260000
H50     6.1396780000    -0.7316170000     5.9218290000
H51     5.8298970000     1.4787000000     5.3358410000
H52     12.1478240000     6.3752850000     11.6878830000
H53     13.1387250000     6.7665950000     10.5379200000
H54     11.7755950000     7.5257340000     10.6901380000
H55     10.2864980000     2.2418030000     4.4021560000
H56     11.8092440000     2.6112790000     4.4519150000
H57     11.3086700000     1.2643200000     5.0784610000
H58     13.8989740000     6.2996240000     8.5704320000
H59     14.5711930000     5.5178590000     6.4677660000
H60     13.2257040000     4.0041610000     5.3224310000


# NewGTO "def2-TZVP(-f)" end NewAuxGTO "def2/J" end
*

%plots format cube
```

```
dim1 100 dim2 100 dim3 100
SpinDens("file_name.cube");
End
```

<u>Sample Input File for Mössbauer Parameter Calculation</u>

```
! UKS B3LYP Direct defgrid2 TIGHTSCF SlowConv def2-SVP UCO PAL8

%basis      NewGTO 26 "CP(PPP)" end
            NewGTO 7 "def2-TZVP(-f)" end
            end

%scf   MaxIter 500
     TolE 1e-7
     TolErr 1e-6
    end

*xyz 0 1
xyz coordinates here
*
%eprnmr nuclei = all Fe {rho, fgrad} end
```

■ Calculated Thermochemical Properties for 1-CH_3 calculated at (298.15K)

```
Total enthalpy                 ...  -2359.93551119 Eh
Total entropy correction       ...     -0.07726737 Eh    -48.49 kcal/mol
------------------------------------------------------------------
Final Gibbs free enthalpy      ...  -2360.01277856 Eh
```

■ Optimized Coordinates for 1-CH_3

Fe	9.970027	3.404823	8.982162
N	10.286529	5.285843	9.932293
N	9.872997	2.653983	7.008379
N	11.621462	4.129489	8.096040
C	9.442324	2.046586	10.435954
C	9.669565	5.241944	12.367564
C	8.669302	5.755066	13.414153
C	7.216698	5.481175	13.003642
C	6.913902	6.039307	11.605678
C	7.922275	5.551411	10.555885
C	9.380664	5.817213	10.965726
C	10.050479	-0.251855	7.480375
C	7.649989	3.876567	5.694623
C	8.829349	0.417323	6.893682
C	7.688570	-0.345381	6.596394
C	6.559517	0.230878	6.016582
C	6.554899	1.596767	5.723361
C	7.661916	2.402220	6.018069
C	8.800464	1.803015	6.612272
C	12.135571	6.826564	10.647186
C	11.267666	1.983067	5.036676
C	11.489437	5.785887	9.767683

C	12.229511	5.224874	8.631110
C	13.375637	5.741241	8.042386
C	13.852114	5.175709	6.848094
C	13.125999	4.152910	6.231753
C	11.968987	3.664890	6.855918
C	10.995117	2.746352	6.306905
H	10.235361	1.941974	11.199591
H	8.516287	2.324110	10.973774
H	9.270724	1.043096	10.008846
H	9.621132	4.142555	12.308286
H	10.697088	5.498365	12.672874
H	8.808621	6.844159	13.552533
H	8.886577	5.289327	14.390507
H	7.036824	4.390282	13.010786
H	6.522893	5.914824	13.743899
H	6.924379	7.145056	11.644330
H	5.895322	5.752408	11.293031
H	7.798347	4.465794	10.404514
H	7.726448	6.030845	9.582422
H	9.510391	6.915192	11.019123
H	10.714505	-0.637267	6.685646
H	10.642152	0.438082	8.096727
H	9.765753	-1.110114	8.107919
H	7.834352	4.491219	6.590812
H	8.443501	4.142412	4.977201
H	6.686203	4.178049	5.257348
H	7.692435	-1.413003	6.836921
H	5.683165	-0.382768	5.792187
H	5.676068	2.054482	5.259444
H	12.710172	6.339173	11.454931
H	12.840258	7.452505	10.082289
H	11.402571	7.491235	11.123671
H	10.349222	1.541360	4.626997
H	11.705452	2.639727	4.268944
H	11.986591	1.162680	5.208801
H	13.888144	6.590256	8.475864
H	14.747728	5.582337	6.373189
H	13.443187	3.759215	5.263838

■ Mössbauer parameters for 1-CH_3

$\rho(0)$= 11816.336670088 x 10^{-3} → 0.53 mm/s
ΔE_Q = 2.199 mm/s

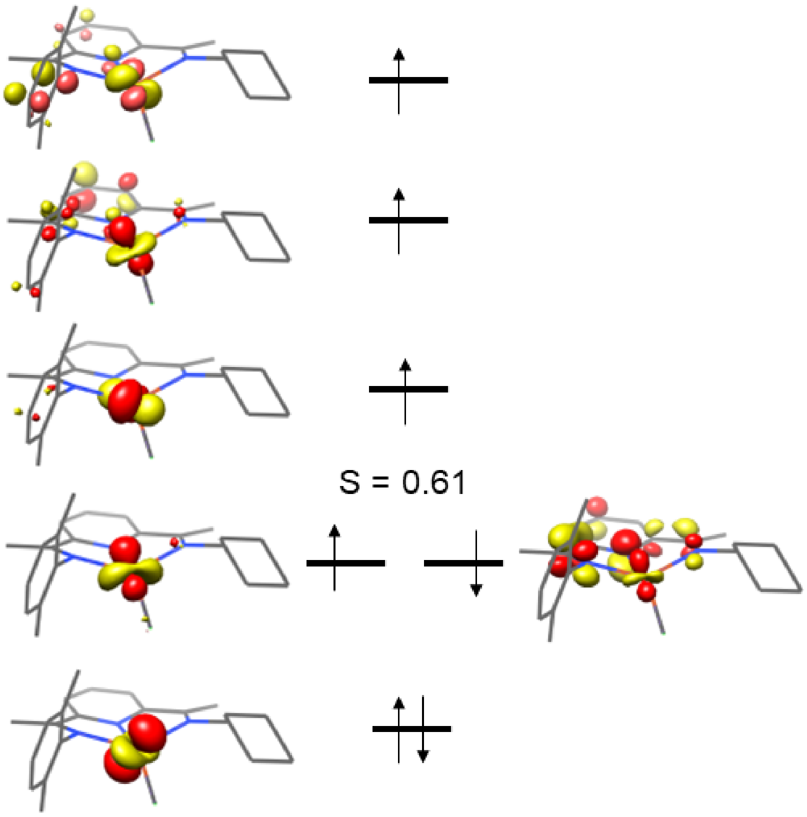

Figure S60. Qualitative molecular orbital diagram of **1-CH_3** BS(4,1) calculation depicting localized orbitals using the B3LYP functional.

iii. ($^{Cy}A^{Me}$(Me/Et)PDI)Fe(N_2)$_2$ ((3-N_2)$_2$)

Input File for Geometry and Frequency Analysis.

```
! UKS B3LYP RIJCOSX def2-SVP def2/J Normalprint SlowConv TightSCF TightOpt UCO
NumFreq defgrid2

%pal nprocs 16
        end

%SCF  MaxIter 5000
         TolE 1e-7
        TolErr 1e-6
        end


%basis NewGTO 26 "def2-TZVP(-f)" end
          NewGTO 7 "def2-TZVP(-f)" end
     NewAuxGTO 26 "def2/J" end
          NewAuxGTO 7 "def2/J" end
    end

*xyz 0 1
xyz coordinates here
*
```

```
%plots format cube
        dim1 100 dim2 100 dim3 100
        SpinDens("file_name.cube");
        end
```

■ Calculated Thermochemical Properties for (3-N$_2$)$_2$ calculated at (298.15K)

Total enthalpy ... -2578.33890394 Eh
Total entropy correction ... -0.08443543 Eh -52.98 kcal/mol

Final Gibbs free energy ... -2578.42333937 Eh

■ Optimized Coordinates for (3-N$_2$)$_2$

Fe	3.710536	3.318622	13.445948
N	4.402505	1.644481	12.992964
N	1.980883	2.802080	13.752528
N	3.777994	5.117826	13.811552
N	3.370283	4.067985	11.598057
N	4.487814	3.279057	15.286120
N	4.824222	0.641073	12.788989
N	0.906391	2.602371	13.933191
C	3.179837	5.380741	11.575759
C	3.390070	6.024979	12.846556
C	3.265541	7.377332	13.167905
C	3.521137	7.813409	14.477271
C	3.909844	6.883848	15.447370
C	4.043080	5.534817	15.104805
C	4.475327	4.447522	15.930029
C	2.801740	6.175840	10.349763
C	4.899015	4.650408	17.368240
C	6.336279	5.164070	17.565939
C	3.242250	3.321418	10.333059
C	2.271411	2.132010	10.433589
C	2.162909	1.393776	9.090558
C	3.537098	0.946430	8.575167
C	4.506997	2.131003	8.474037
C	4.624648	2.884208	9.807134
C	5.033105	2.120205	15.931262
C	4.222857	1.305440	16.754709
C	4.796231	0.165627	17.338884
C	6.130503	-0.170865	17.112746
C	6.914666	0.638108	16.290668
C	6.387677	1.789587	15.687127
C	2.770740	1.630281	17.009341
C	7.255897	2.637688	14.788754
H	2.969234	8.098770	12.404935
H	3.419430	8.869584	14.734057
H	4.114159	7.208684	16.469596
H	3.558373	6.088048	9.552540
H	2.704882	7.241977	10.587698
H	1.838933	5.847412	9.924573
H	4.779854	3.706729	17.919682

H	4.199172	5.365892	17.831798
H	7.076868	4.423721	17.229346
H	6.527953	5.363730	18.632867
H	6.514493	6.097644	17.009162
H	2.817505	3.986809	9.565488
H	1.281091	2.494371	10.754571
H	2.612367	1.427800	11.207407
H	1.692407	2.057858	8.341072
H	1.490426	0.526130	9.197797
H	3.955906	0.191027	9.265474
H	3.435530	0.450212	7.595003
H	4.151735	2.825373	7.689358
H	5.504269	1.786959	8.151439
H	5.265784	3.774481	9.693820
H	5.115708	2.242499	10.554834
H	4.177130	-0.465454	17.983250
H	6.558582	-1.063970	17.575564
H	7.960424	0.375957	16.105895
H	2.369853	1.008646	17.823570
H	2.154942	1.444770	16.115950
H	2.617735	2.686158	17.279294
H	7.366258	3.665750	15.168128
H	6.822340	2.733542	13.781545
H	8.261487	2.202126	14.694297

Mössbauer parameters for (3-N_2)$_2$

ρ(0)= 11816.636426565 x 10^{-3} → 0.42 mm/s
ΔE_Q = -0.665 mm/s

iv. ($^{Cy}A^{Me}$PDI)Fe(η^1,η^3-C_6H_{10})

Sample Input File for Geometry and Frequency Analysis

```
!UKS Opt TightOpt TightSCF TPSSh def2-TZVP RIJCOSX SARC/J UNO UCO Freq

%MaxCore 3000

%SCF
	MaxIter 5000
end

%pal nprocs 8 end

*xyz 0 1
xyz coordinates here
*
```

Sample Input File for Single Point Electronic Energy Calculation with Optimized Geometry.

```
! UKS TPSSh D3BJ DKH-def2-TZVPP RIJCOSX Normalprint TightSCF UCO UNO
CPCM(benzene)

%pal nprocs 8
```

```
        end

%SCF
        MaxIter 5000
   end

%MaxCore 3000


*xyz 0 1
xyz coordinates here
*

%plots format cube
        dim1 100 dim2 100 dim3 100
        SpinDens("file_name.cube");
        end
```

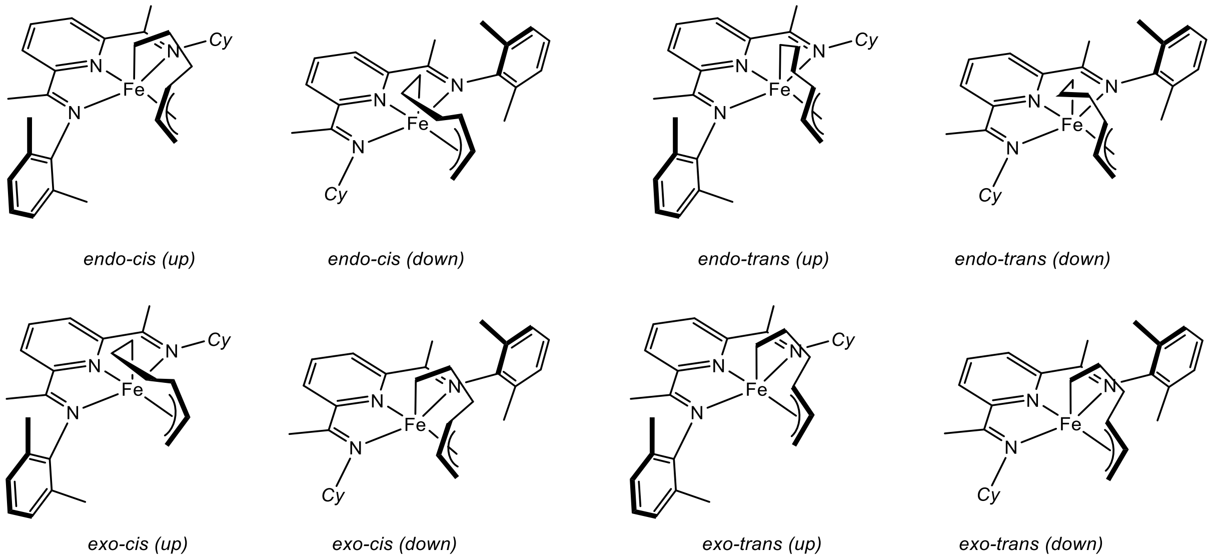

Figure S61. Possible isomers and enantiomers calculated for ($^{Cy}A^{Me}$PDI)Fe(η^1,η^3-C_6H_{10}) complexes.

Table S14. Calculated ($^{Cy}A^{Me}$PDI)Fe(η^1,η^3-C_6H_{10}) energies and Mössbauer parameters.

**Complex**	imaginary frequencies	Electronic energy [Ha]	G-E(el) [Ha]	Final Gibbs free energy [Ha]	Relative ΔG [kcal.mol^{-1}]	δ [mm/s]	$\lvert\Delta E_Q\rvert$ [mm/s]
trans endo up	0	-2555.6797	0.5623	-2555.1174	3.36	0.28	1.27
trans endo down	0	-2555.6855	0.5628	-2555.1227	0.00	0.29	1.07
trans exo up	0	-2555.6811	0.5626	-2555.1185	2.64	0.31	0.95
trans exo down	1	-2555.6837	0.5639	-2555.1198	1.83	0.28	1.40
cis endo up	0	-2555.6700	0.5630	-2555.1070	9.88	0.31	0.85
cis endo down	0	-2555.6699	0.5629	-2555.1070	9.85	0.31	0.85
cis exo up	0	-2555.6612	0.5630	-2555.0983	15.35	0.33	1.17
cis exo down	0	-2555.6603	0.5619	-2555.0984	15.25	0.34	1.20

Optimized Coordinates trans endo up

Fe	5.523398	4.266248	10.831499
N	4.330381	3.634844	9.315558
N	6.697420	5.572789	11.793281
N	6.273786	5.147249	9.421670
C	3.152252	1.405596	9.032711
C	1.866823	0.669184	9.428196
C	0.626593	1.352754	8.845598
C	0.558126	2.823723	9.266871
C	1.825873	3.584659	8.858923
C	3.062873	2.885447	9.449818
C	5.071904	7.914932	11.911375
C	8.113789	4.544317	14.218025
C	5.675231	7.309163	13.152501
C	5.432132	7.898192	14.393127
C	6.020028	7.406690	15.550899
C	6.878227	6.320484	15.468028
C	7.139254	5.689661	14.248993
C	6.509552	6.172016	13.089816
C	4.164780	3.633990	6.771885
C	8.562150	7.222285	11.500253
C	4.762984	3.973050	8.113064
C	5.919619	4.814147	8.138051
C	6.630217	5.306435	7.047868
C	7.742042	6.122542	7.252746
C	8.127991	6.424713	8.553052
C	7.389898	5.932556	9.628546
C	7.568057	6.209379	11.010621
C	3.868320	4.687147	12.099233
C	4.706361	3.700781	12.674523
C	4.962677	2.506963	12.008919
C	6.063946	1.532862	12.364669
C	7.365897	2.049015	11.737962
C	7.023949	2.896411	10.517987
H	2.862425	2.862588	10.521644
H	7.371576	5.950218	16.361159
H	3.307984	1.304319	7.955503
H	4.020958	0.948321	9.514684
H	1.784288	0.648488	10.521955
H	1.920379	-0.372609	9.097580
H	-0.278442	0.826667	9.164448
H	0.658434	1.289853	7.750312
H	-0.319987	3.306029	8.826600
H	0.435432	2.880139	10.355541
H	1.791958	4.613841	9.228640
H	1.871931	3.638041	7.769679
H	5.792285	8.564069	11.401374
H	4.765844	7.145842	11.199182
H	4.207364	8.529318	12.170912
H	8.822506	4.630718	15.044397
H	7.603315	3.582731	14.324688
H	8.672480	4.514464	13.281920

H	4.776281	8.761624	14.441916
H	5.823818	7.877593	16.507569
H	3.477505	2.796612	6.804850
H	3.631133	4.494310	6.354099
H	4.963364	3.376128	6.072592
H	8.882255	7.004820	12.519218
H	9.443276	7.229857	10.856190
H	8.143939	8.235137	11.496824
H	6.318147	5.052946	6.042919
H	8.302797	6.506558	6.409710
H	8.996804	7.043921	8.738064
H	3.792537	5.638118	12.611817
H	2.976861	4.405121	11.553888
H	4.185093	2.101691	11.380279
H	5.340920	3.984930	13.507989
H	6.156888	1.402478	13.449375
H	5.807744	0.551296	11.955224
H	7.893076	2.668704	12.461853
H	8.049818	1.223194	11.489967
H	7.904836	3.455286	10.182150
H	6.679180	2.276826	9.681810

Optimized Coordinates trans endo down

Fe	5.090214	4.876161	11.071092
N	6.157310	5.130813	9.610365
N	6.511425	6.067247	11.844727
N	4.285818	3.527270	9.790432
C	7.349222	6.584061	10.953099
C	7.132380	6.103612	9.631556
C	7.814685	6.466087	8.471638
C	7.498469	5.853969	7.264483
C	6.479648	4.903471	7.235448
C	5.810330	4.566623	8.408537
C	4.751332	3.614524	8.557788
C	8.486768	7.508268	11.278165
C	4.348579	2.818000	7.343192
C	6.849806	6.293633	13.222333
C	7.771376	5.424049	13.837563
C	8.030272	5.582361	15.199991
C	7.425533	6.592941	15.935356
C	6.573119	7.484702	15.298572
C	6.281040	7.360475	13.939177
C	8.524407	4.378594	13.053386
C	5.443810	8.410605	13.258050
C	3.305776	2.471879	10.128891
C	1.888864	2.708269	9.575035
C	0.915213	1.680248	10.163833
C	1.394232	0.246071	9.920618
C	2.804549	0.033806	10.478547
C	3.799966	1.035347	9.880879
C	2.610796	6.702286	10.953868
C	3.987027	6.449306	10.319538
C	2.639637	6.200737	12.399983
C	3.305363	4.859005	12.297886

C	4.424770	4.463679	13.018968
C	5.241480	3.385865	12.606925
H	8.587103	7.223408	8.519725
H	8.024169	6.122765	6.356717
H	6.204165	4.427325	6.303204
H	8.325090	8.026480	12.222393
H	8.606319	8.255080	10.490236
H	9.433245	6.961909	11.356013
H	5.126889	2.094474	7.078576
H	4.228976	3.492136	6.491422
H	3.416723	2.279843	7.467992
H	7.630035	6.694631	16.995252
H	6.125014	8.300070	15.857802
H	7.960172	4.039595	12.184989
H	8.757862	3.517907	13.684065
H	9.477090	4.785001	12.695039
H	4.678848	8.800981	13.932920
H	4.964172	8.027873	12.359826
H	6.072088	9.257000	12.956530
H	3.194085	2.528602	11.212437
H	1.867488	2.639870	8.484402
H	1.571488	3.724034	9.826983
H	0.821860	1.848934	11.243832
H	-0.081088	1.828751	9.736291
H	1.395988	0.041378	8.842286
H	0.697607	-0.465702	10.374047
H	3.144113	-0.987373	10.279877
H	2.781589	0.151260	11.569007
H	4.786091	0.916825	10.339956
H	3.918843	0.826563	8.815772
H	8.722705	4.900536	15.683671
H	1.853806	6.124459	10.410388
H	2.300188	7.754545	10.885363
H	1.633106	6.133994	12.833620
H	3.227296	6.867099	13.035078
H	4.655162	7.309158	10.446835
H	3.889323	6.274837	9.241890
H	4.815345	2.487087	12.182490
H	6.165468	3.224976	13.147430
H	4.828735	5.161604	13.744046
H	2.713994	4.119288	11.775706

Optimized Coordinates trans exo up

Fe	5.325096	4.494793	10.791813
N	6.190699	5.266068	9.379298
N	6.638661	5.766968	11.756512
N	4.320410	3.651381	9.262332
C	7.513205	6.343657	10.948939
C	7.306863	6.049315	9.570310
C	8.060931	6.516556	8.496429
C	7.687120	6.196399	7.196771
C	6.566607	5.391147	7.000842
C	5.844725	4.923555	8.094094
C	4.729883	4.026195	8.065586

C	8.603868	7.281565	11.383748
C	4.170951	3.626271	6.724182
C	6.632946	6.238261	13.112798
C	7.271539	5.509952	14.131211
C	7.169523	5.960978	15.448421
C	6.472156	7.119462	15.759234
C	5.897786	7.863887	14.737691
C	5.980032	7.454116	13.406353
C	8.131729	4.315678	13.816043
C	5.421141	8.349012	12.327153
C	3.283925	2.599574	9.382041
C	1.854274	3.116574	9.150032
C	0.832951	2.039830	9.535159
C	1.097967	0.722857	8.800341
C	2.528499	0.232632	9.044338
C	3.559250	1.288316	8.623280
C	6.412226	1.817308	11.897449
C	6.599245	2.865683	10.777366
C	5.047614	2.013480	12.571183
C	4.821312	3.501397	12.620766
C	3.706188	4.131107	12.074136
C	3.703256	5.508335	11.778015
H	8.931594	7.132576	8.683770
H	8.261074	6.556068	6.352049
H	6.266681	5.109853	5.999456
H	8.362580	8.318518	11.128040
H	9.538822	7.031513	10.876189
H	8.769272	7.229022	12.458466
H	4.765779	2.833387	6.259686
H	4.194722	4.488448	6.054375
H	3.141703	3.287064	6.782531
H	6.390528	7.449953	16.788612
H	5.376962	8.787911	14.968352
H	7.921314	3.929277	12.821670
H	7.984467	3.518610	14.548402
H	9.190769	4.595684	13.849158
H	4.534064	8.874058	12.688071
H	5.157558	7.793375	11.427636
H	6.156729	9.111029	12.046742
H	1.705869	3.400093	8.103534
H	1.701171	4.021944	9.743707
H	0.886933	1.863510	10.616663
H	-0.179908	2.397679	9.326616
H	0.945499	0.870540	7.723464
H	0.377957	-0.037513	9.118169
H	2.710889	-0.698054	8.498406
H	2.654423	0.002253	10.109387
H	4.572125	0.948648	8.856925
H	3.502075	1.414930	7.541465
H	7.655071	5.391822	16.235033
H	7.190058	1.941635	12.656360
H	6.523370	0.790637	11.523842
H	7.613481	3.282597	10.791542
H	6.459675	2.415323	9.790375
H	5.014301	1.550680	13.566309

H	4.259102	1.539470	11.978547
H	5.448370	4.055405	13.310568
H	2.927157	3.515331	11.643739
H	4.162165	6.200731	12.467396
H	2.880786	5.911105	11.196024
H	3.325356	2.300001	10.426965

Optimized Coordinates trans exo down

Fe	4.911644	4.951025	10.841783
N	4.155678	3.745984	9.399915
N	6.422051	5.893049	11.813938
N	6.061837	5.305120	9.469552
C	3.851789	1.252885	9.052354
C	2.996281	0.073117	9.528699
C	1.533016	0.244569	9.111770
C	0.964385	1.568281	9.630700
C	1.804481	2.767244	9.171960
C	3.271337	2.572532	9.593748
C	5.528733	8.220989	13.337600
C	8.081622	3.856004	12.992784
C	6.201965	7.041358	13.988243
C	6.420730	7.033965	15.367183
C	7.115489	5.999052	15.977908
C	7.629671	4.968039	15.201856
C	7.435519	4.936357	13.820408
C	6.679160	5.963309	13.223122
C	4.185299	3.427984	6.871820
C	8.554529	7.194437	11.485151
C	4.619976	4.014598	8.190100
C	5.711520	4.937842	8.191124
C	6.442388	5.378581	7.092783
C	7.556335	6.196998	7.277909
C	7.904942	6.577111	8.568657
C	7.142203	6.138903	9.649031
C	7.353045	6.418936	11.023877
C	4.734249	3.437636	12.309753
C	3.536221	4.189720	12.216459
C	3.569684	5.549928	12.492764
C	2.445041	6.486204	12.118572
C	2.376782	6.506271	10.591997
C	3.810712	6.496864	10.078279
H	3.240282	2.422896	10.670673
H	8.198942	4.170456	15.668529
H	3.884091	1.242880	7.961234
H	4.881569	1.149493	9.406953
H	3.054047	-0.001282	10.621695
H	3.400411	-0.861888	9.128431
H	0.933190	-0.593236	9.480438
H	1.464045	0.223663	8.016590
H	-0.071084	1.695112	9.300201
H	0.943198	1.542835	10.727361
H	1.419595	3.689513	9.613958
H	1.712078	2.879051	8.088018
H	6.270122	8.977270	13.055586

H	5.001151	7.929824	12.430171
H	4.824565	8.694937	14.024591
H	9.012420	4.221573	12.544547
H	8.332162	2.995122	13.615606
H	7.435751	3.532565	12.174541
H	6.040711	7.858471	15.962172
H	7.267814	6.002446	17.051359
H	3.213789	2.947810	6.919192
H	4.120066	4.228503	6.130767
H	4.911369	2.698418	6.498321
H	8.580743	7.288361	12.569076
H	9.480446	6.710407	11.159577
H	8.546923	8.200474	11.054081
H	6.148818	5.073990	6.096158
H	8.136970	6.533757	6.428179
H	8.762986	7.214240	8.745287
H	4.735216	2.394266	12.011499
H	5.420166	3.676184	13.113348
H	4.284481	5.890774	13.229652
H	2.652020	3.789273	11.732717
H	1.494012	6.188283	12.577999
H	2.673101	7.488395	12.491376
H	1.857225	5.610031	10.239538
H	1.797015	7.364436	10.220623
H	3.846669	6.402999	8.986754
H	4.329903	7.423779	10.353374

Optimized Coordinates cis endo up

Fe	5.159642	4.732753	11.179603
N	6.016123	5.300482	9.661049
N	6.543885	5.863347	11.988694
N	3.962537	3.842648	9.630556
C	7.441298	6.370129	11.140201
C	7.182124	6.033878	9.788874
C	7.950634	6.364315	8.670660
C	7.569421	5.929776	7.411485
C	6.423326	5.139947	7.292428
C	5.674840	4.830064	8.419876
C	4.467103	4.044708	8.439866
C	8.589880	7.245508	11.551203
C	3.925406	3.594006	7.105518
C	6.682955	6.291980	13.354254
C	7.424016	5.529559	14.273247
C	7.512455	5.975762	15.593421
C	6.896562	7.151581	16.000427
C	6.193212	7.910139	15.074615
C	6.082410	7.503572	13.744349
C	8.166755	4.291365	13.850399
C	5.348013	8.368333	12.753187
C	2.679400	3.117127	9.770144
C	1.468771	3.790571	9.095201
C	0.178349	3.083123	9.525727
C	0.230755	1.584912	9.212744
C	1.448798	0.926275	9.866832

C	2.754769	1.612529	9.448859
C	3.236787	5.492550	11.796473
C	6.201473	2.959419	11.083671
C	5.655405	1.854843	12.013976
C	3.964496	4.995097	12.891437
C	4.338664	2.334408	12.639108
C	4.613383	3.762044	13.027411
H	8.846793	6.958401	8.797582
H	8.158492	6.180150	6.538139
H	6.117441	4.769405	6.322882
H	8.292787	8.298198	11.615936
H	9.402726	7.177524	10.827224
H	8.977870	6.959134	12.529012
H	4.725697	3.127405	6.526212
H	3.567142	4.454985	6.532468
H	3.115917	2.880194	7.184477
H	6.969136	7.477417	17.031982
H	5.716648	8.836022	15.380796
H	7.534712	3.626669	13.262902
H	8.537398	3.752131	14.724049
H	9.028781	4.543839	13.223236
H	4.604304	8.986361	13.260092
H	4.856364	7.767349	11.987801
H	6.038858	9.044626	12.237589
H	2.453792	3.160182	10.836753
H	1.545633	3.760621	8.006363
H	1.440409	4.846333	9.380110
H	0.033328	3.220604	10.604290
H	-0.680551	3.547049	9.031233
H	0.281411	1.443301	8.125555
H	-0.688533	1.096752	9.550546
H	1.492294	-0.136581	9.610361
H	1.346051	0.982583	10.957620
H	3.603703	1.174887	9.979231
H	2.926481	1.438712	8.383852
H	8.080092	5.386183	16.306211
H	5.528961	0.895759	11.495472
H	2.515302	4.900923	11.259790
H	6.145060	2.663075	10.032693
H	3.015969	6.554943	11.797351
H	3.518535	2.241981	11.932666
H	7.250620	3.187763	11.292730
H	6.369759	1.666648	12.824728
H	4.346464	5.756835	13.562735
H	4.065388	1.736768	13.521018
H	5.372996	3.801137	13.807279

Optimized Coordinates cis endo down

Fe	5.167845	4.816295	11.011748
N	5.993231	5.380551	9.474523
N	6.585757	5.923159	11.789786
N	3.924880	3.943802	9.491260
C	7.466479	6.427008	10.921513
C	7.169506	6.102740	9.575263

C	7.911961	6.428767	8.437860
C	7.495171	6.001024	7.187953
C	6.338948	5.222071	7.095844
C	5.616335	4.917273	8.240950
C	4.402333	4.142814	8.289109
C	8.636524	7.279457	11.319083
C	3.826358	3.696306	6.967718
C	6.777602	6.335621	13.154066
C	7.543499	5.555671	14.037516
C	7.688566	5.989038	15.356755
C	7.103716	7.168676	15.797360
C	6.373900	7.943697	14.906376
C	6.207170	7.550530	13.577789
C	8.247812	4.308317	13.577775
C	5.446976	8.432741	12.622065
C	2.639252	3.229048	9.661090
C	1.418274	3.912077	9.015011
C	0.132949	3.213534	9.474384
C	0.167362	1.715342	9.158732
C	1.394762	1.047233	9.785039
C	2.695936	1.724089	9.337930
C	3.269768	5.598009	11.674711
C	4.017134	5.092696	12.752818
C	4.350152	2.427935	12.492653
C	4.653116	3.851612	12.874530
C	5.644014	1.931166	11.834001
C	6.181002	3.027164	10.888318
H	8.817152	7.013357	8.541814
H	8.064534	6.248288	6.300766
H	6.005451	4.856443	6.133581
H	8.329304	8.303444	11.556507
H	9.367012	7.331028	10.512019
H	9.133863	6.881522	12.205240
H	4.607910	3.216702	6.373571
H	3.468597	4.560710	6.399607
H	3.008611	2.994444	7.065619
H	7.220572	7.484404	16.827994
H	5.920500	8.872194	15.238704
H	7.571449	3.643097	13.042388
H	8.672237	3.774556	14.429982
H	9.066036	4.546309	12.889659
H	4.726639	9.051849	13.160531
H	4.924350	7.844692	11.867203
H	6.125362	9.109117	12.089987
H	2.439825	3.274452	10.732760
H	1.469653	3.883022	7.924722
H	1.404124	4.967682	9.301531
H	0.013384	3.350777	10.556092
H	-0.733585	3.684253	9.000051
H	0.193141	1.574785	8.070529
H	-0.747834	1.233416	9.516014
H	1.424606	-0.015780	9.527311
H	1.317111	1.103867	10.877869
H	3.553427	1.280132	9.848921
H	2.842368	1.549681	8.269217

H	8.275801	5.386106	16.041973
H	3.062756	6.663186	11.678820
H	3.511120	2.346417	11.807438
H	2.528469	5.015137	11.155606
H	4.091838	1.833293	13.381054
H	4.426029	5.850426	13.412698
H	5.431412	3.880196	13.636122
H	5.492437	0.972765	11.320898
H	6.376435	1.734435	12.626531
H	7.238321	3.239934	11.069233
H	6.092332	2.731222	9.839565

Optimized Coordinates cis exo up

Fe	5.069232	4.680785	10.990505
N	5.971223	5.385449	9.545140
N	6.748193	5.997224	11.912423
N	3.972398	3.915552	9.572418
C	7.534059	6.432414	10.962878
C	7.169799	6.055174	9.625126
C	7.902657	6.396686	8.495069
C	7.432442	6.060094	7.227586
C	6.238752	5.358910	7.136802
C	5.533978	5.011306	8.288543
C	4.361358	4.209206	8.335984
C	8.729566	7.323661	11.178854
C	3.734854	3.774024	7.035787
C	6.947687	6.558774	13.217000
C	7.725143	5.897969	14.184705
C	7.805998	6.442466	15.468489
C	7.158198	7.624995	15.792899
C	6.453747	8.306848	14.809296
C	6.351874	7.804603	13.512318
C	8.549232	4.681014	13.852384
C	5.679487	8.636019	12.448935
C	2.817531	3.009577	9.773680
C	1.453920	3.648769	9.452122
C	0.322157	2.725677	9.919184
C	0.455266	1.325989	9.311923
C	1.821301	0.711079	9.632373
C	2.968768	1.609292	9.153023
C	3.456094	4.598298	12.345747
C	3.883587	5.930957	12.283950
C	4.263741	3.488824	12.603564
C	5.491630	3.256456	13.437225
C	6.379807	2.432505	12.497189
C	6.431697	3.155836	11.135289
H	8.836453	6.931678	8.609272
H	7.991507	6.324286	6.338927
H	5.857117	5.055860	6.170113
H	8.572539	8.304491	10.720817
H	9.614622	6.883436	10.712097
H	8.932843	7.465885	12.237976
H	4.290649	2.944580	6.585172

H	3.752860	4.603784	6.325616
H	2.702189	3.461861	7.141437
H	7.220487	8.025716	16.798374
H	5.978198	9.254602	15.041197
H	8.317448	4.292810	12.863387
H	8.391367	3.884407	14.583115
H	9.614196	4.936449	13.874959
H	4.895710	9.257700	12.886458
H	5.245156	8.021975	11.660996
H	6.406156	9.309590	11.980186
H	2.796362	2.819993	10.842967
H	1.340293	3.840480	8.382004
H	1.389892	4.620270	9.950441
H	0.348629	2.646262	11.013088
H	-0.646091	3.164466	9.659444
H	0.333677	1.390726	8.223040
H	-0.346252	0.677843	9.679422
H	1.910242	-0.278688	9.174161
H	1.903809	0.564462	10.716455
H	3.933346	1.191960	9.454872
H	2.959883	1.643008	8.062191
H	8.399431	5.926313	16.216824
H	3.190454	6.666685	11.889912
H	2.498117	4.387697	11.881589
H	3.801924	2.537968	12.338999
H	4.595252	6.307060	13.003659
H	5.251984	2.715149	14.364606
H	5.968754	4.193941	13.720110
H	6.286412	2.464568	10.300061
H	5.908663	1.450164	12.369974
H	7.372953	2.240931	12.919648
H	7.392383	3.657541	10.977224

Optimized Coordinates cis exo down

Fe	5.136771	4.438336	11.047748
N	6.162091	4.994643	9.625998
N	6.568224	5.836764	11.957140
N	4.016112	3.798350	9.572875
C	7.527428	6.159571	11.113123
C	7.356697	5.658970	9.782737
C	8.217620	5.870607	8.713036
C	7.864812	5.428461	7.438923
C	6.647682	4.777858	7.271224
C	5.810326	4.559724	8.363097
C	4.546635	3.899816	8.360910
C	8.682390	7.066698	11.441702
C	3.978501	3.390799	7.062344
C	6.566566	6.579939	13.187134
C	7.179189	6.076891	14.352294
C	7.089725	6.817416	15.531302
C	6.432241	8.039711	15.569521
C	5.899325	8.558292	14.399326
C	5.973801	7.857966	13.193669
C	7.985885	4.802280	14.356426

C	5.472612	8.529713	11.938866
C	2.658042	3.249150	9.766214
C	1.547268	4.164743	9.216727
C	0.177047	3.664221	9.686434
C	-0.055267	2.204416	9.285906
C	1.069514	1.303054	9.803380
C	2.443490	1.789683	9.325232
C	6.473041	2.938614	11.602579
C	5.158499	2.510121	11.872942
C	3.623011	5.554529	11.926063
C	4.240945	3.210828	12.653527
C	3.571259	5.375306	13.465986
C	4.397894	4.130634	13.828362
H	9.153543	6.390731	8.871906
H	8.522715	5.594541	6.595235
H	6.349369	4.430327	6.290182
H	8.867198	7.103933	12.513922
H	8.485219	8.088539	11.101672
H	9.590635	6.724740	10.942223
H	4.332804	2.375818	6.851126
H	4.299288	4.028962	6.237595
H	2.893332	3.369455	7.053074
H	6.362761	8.595222	16.497906
H	5.421472	9.533093	14.407171
H	8.563791	4.687435	13.436808
H	7.357595	3.912514	14.458151
H	8.681247	4.807728	15.198159
H	4.508400	9.010983	12.118362
H	5.360998	7.833172	11.110688
H	6.171541	9.315128	11.629374
H	2.527043	3.256816	10.847309
H	1.561030	4.192323	8.123580
H	1.723815	5.185812	9.561761
H	0.116323	3.753620	10.777933
H	-0.611302	4.302197	9.275090
H	-0.101250	2.132349	8.191637
H	-1.021670	1.857018	9.664231
H	0.909354	0.270024	9.479468
H	1.050104	1.296050	10.900071
H	3.233295	1.157404	9.740207
H	2.495835	1.691070	8.238553
H	7.560512	6.427059	16.427980
H	7.019096	2.424195	10.818656
H	4.743896	1.757800	11.208594
H	7.088441	3.387882	12.367651
H	3.785851	6.594129	11.638774
H	2.666431	5.257647	11.491602
H	3.942991	6.250270	14.002880
H	3.213015	2.872835	12.535857
H	5.440185	4.403438	13.970185
H	2.531577	5.236459	13.789970
H	4.055554	3.656399	14.759180

[i] Pangborn, A. B.; Giardello, M. A.; Grubbs, R. H.; Rosen, R. K.; Timmers, F. J. Safe and Convenient Procedure for Solvent Purification. *Organometallics* **1996**, *15*, 1518–1520.

[ii] Russell, S. K.; Lobkovsky, E.; Chirik, P. J. Iron-Catalyzed Intermolecular [2π + 2π] Cycloaddition. *J. Am. Chem. Soc.* **2011**, *133* (23), 8858– 8861.

[iii] Bianchini, C.; Mantovani, G.; Meli, A.; Migliacci, F.; Zanobini, F.; Laschi, F.; Sommazzi, A. Oligomerisation of Ethylene to Linear a-Olefins by new C_s- and C_1-Symmetric [2,6-Bis(imino)pyridyl]iron and -cobalt Dichloride Complexes. *Eur. J. Inorg. Chem.* **2003**, *2003*, 1620– 1631.

[iv] Russell, S. K.; Lobkovsky, E.; Chirik, P. J. Iron-Catalyzed Intermolecular [2π + 2π] Cycloaddition. *J. Am. Chem. Soc.* **2011**, *133* (23), 8858– 8861.

[v] Ion Prisecaru, WMOSS4 Mössbauer Spectral Analysis Software, www.wmoss.org, 2009–2016.

[vi] Bart, S. C.; Lobkovsky, E.; Chirik, P. J. Preparation and Molecular and Electronic Structures of Iron(0) Dinitrogen and Silane Complexes and Their Application to Catalytic Hydrogenation and Hydrosilation. *J. Am. Chem. Soc.* **2004**, *126*, 13794–13807.

[vii] Russell, S. K.; Lobkovsky, E.; Chirik, P. J. Iron-Catalyzed Intermolecular [2π + 2π] Cycloaddition. *J. Am. Chem. Soc.* **2011**, *133* (23), 8858– 8861.

[viii] Neese, F. *ORCA: an ab initio, DFT and Semiempirical Electronic Structure Package*, Version 2.8, Revision 2287; Institut für Physikalische und Theoretische Chemie, Universität Bonn: Bonn, Germany, 2010

[ix] Perdew, J. P. Density-functional approximation for the correlation energy of the inhomogeneous electron gas. *Phys. Rev. B* **1986**, *33*, 8822–8824.

[x] Perdew, J. P. Erratum: Density-functional approximation for the correlation energy of the inhomogeneous electron gas. *Phys. Rev. B* **1986**, *34*, 7406.

[xi] Lee, C. T.; Yang, W. T.; Parr, R. G. Development of the Colle-Salvetti correlation-energy formula into a functional of the electron density. *Phys. Rev. B* **1988**, *37*, 785–789.

[xii] Staroverov, V. N.; Scuseria, G. E.; Tao, J. M.; Perdew, J. P. Comparative assessment of a new nonempirical density functional: Molecules and hydrogen-bonded complexes. *J. Chem. Phys.* **2003**, *119*, 12129– 12137.

[xiii] Tao, J. M.; Perdew, J. P.; Staroverov, V. N.; Scuseria, G. E. Climbing the density functional ladder: Nonempirical meta-generalized gradient approximation designed for molecules and solids. *Phys. Rev. Lett.* **2003**, *91*, 146401.

[xiv] Neese, F.; Solomon, E. I. In *Magnetism: From Molecules to Materials;* Miller, J. S.; Drillon, M., Eds.; Wiley: New York, 2002; Vol. 4, p 345.

[xv] Kepp, K. P. Theoretical Study of Spin Crossover in 30 Iron Complexes. *Inorg. Chem.* **2016**, *55*, 2717– 2727.

[xvi] Cirera, J.; Via-Nadal, M.; Ruiz, E. Benchmarking Density Functional Methods for Calculation of State Energies of First Row Spin-Crossover Molecules. *Inorg. Chem.* **2018**, *57*, 14097– 14105.

[xvii] Schäfer, A.; Horn, H.; Ahlrichs, R. Fully optimized contracted Gaussian basis sets for atoms Li to Kr. *J. Chem. Phys.* **1992**, *97*, 2571–2577.

[xviii] Schäfer, A.; Huber, C.; Ahlrichs, R. Fully optimized contracted Gaussian basis sets of triple zeta valence quality for atoms Li to Kr. *J. Chem. Phys.* **1994**, *100*, 5829–5835.

[xix] Weigend, F.; Ahlrichs, R. Balanced basis sets of split valence, triple zeta valence and quadruple zeta valence quality for H to Rn: Design and assessment of accuracy. *Phys. Chem. Chem. Phys.* **2005**, *7*, 3297–3305.

[xx] Eichkorn, K.; Weigend, F.; Treutler, O.; Ahlrichs, R. Auxiliary basis sets for main row atoms and transition metals and their use to approximate Coulomb potentials. *Theor. Chem. Acc.* **1997**, *97*, 119–124.

[xxi] Eichkorn, K.; Treutler, O.; Öhm, H.; Häser, m.; Ahlrichs, R. Auxiliary basis sets to approximate Coulomb potentials. *Chem. Phys. Lett.* **1995**, *240*, 283–289.

[xxii] Eichkorn, K.; Treutler, O.; Öhm, H.; Häser, M.; Ahlrichs, R. Auxiliary basis sets to approximate Coulomb potentials. *Chem. Phys. Lett.* **1995**, *242*, 652–660.

[xxiii] Neese, F.; Wennmohs, F.; Hansen, A.; Becker, U. Efficient, Approximate and parallel Hartree–Fock and hybrid DFT calculations. A ‘chain-of-spheres’ algorithm for the Hartree–Fock exchange. *Chem. Phys.* **2009**, *356*, 98–109.

[xxiv] Kossmann, S.; Neese, F. Comparison of two efficient approximate Hartee–Fock approaches. *Chem. Phys. Lett.* **2009**, *481*, 240–243.

[xxv] Neese, F. An improvement of the resolution of the identity approximation for the formation of the Coulomb matrix. *J. Comput. Chem.* **2003**, *24*, 1740–1747.

[xxvi] Douglas, M.; Kroll, N. M. Quantum electrodynamical corrections to fine-structure of Helium. *Ann. Phys.* **1974**, *82*, 89– 15.

[xxvii] Hess, B. A. Applicability of the no-pair equation with free-particle projection operators to atomic and molecular-structure calculations. *Phys. Rev. A: At., Mol., Opt. Phys.* **1985**, *32*, 756– 763.

[xxviii] Hess, B. A. Relativistic electronic-structure calculations employing a 2-component no-pair formalism with external-field projection operators. *Phys. Rev. A: At., Mol., Opt. Phys.* **1986**, *33*, 3742– 3748.

[xxix] Jansen, G.; Hess, B. A. Revision of the Douglas-Kroll transformation. *Phys. Rev. A: At., Mol., Opt. Phys.* **1989**, *39*, 6016– 6017.

[xxx] Ginsberg, A. P. Magnetic exchange in transition metal complexes. 12. Calculation of cluster exchange coupling constants with the Xα-scattered wave method. *J. Am. Chem. Soc.* **1980**, *102*, 111–117.

[xxxi] Noodleman, L.; Peng, C. Y.; Case, D. A.; Mouesca, J. M. Orbital interactions, electron delocalization and spin coupling in iron-sulfur clusters. *Coord. Chem. Rev.* **1995**, *144*, 199–244.

[xxxii] Kirchner, B.; Wennmohs, F.; Ye, S.; Neese, F. Theoretical bioinorganic chemistry: The electronic structure makes a difference. *Curr. Opin. Chem. Biol.* **2007**, *11*, 134–141.

[xxxiii] Neese, F. *J.* Definition of corresponding orbitals and the diradical character in broken symmetry DFT calculations on spin coupled systems. *Phys. Chem. Solids* **2004**, *65*, 781–785.

[xxxiv] Pettersen, E. F.; Goddard, T. D.; Huang, C. C.; Couch, G. S.; Greenblatt, D. M.; Meng, E. C.; Ferrin, T. E. UCSF Chimera—A visualization system for exploratory research and analysis. *J. Comput. Chem.* **2004**, *25*, 1605–1612.
